# Light-driven, heterogeneous organocatalysts for C–C bond formation toward valuable perfluoroalkylated intermediates

**DOI:** 10.1126/sciadv.abc9923

**Published:** 2020-11-11

**Authors:** Giacomo Filippini, Francesco Longobardo, Luke Forster, Alejandro Criado, Graziano Di Carmine, Lucia Nasi, Carmine D’Agostino, Michele Melchionna, Paolo Fornasiero, Maurizio Prato

**Affiliations:** 1 Department of Chemical and Pharmaceutical Sciences, INSTM, University of Trieste, Via L. Giorgieri 1, 34127 Trieste, Italy.; 2 Department of Chemical Engineering and Analytical Science, The University of Manchester, Oxford Road, Manchester M13 9PL, UK.; 3 CIC biomaGUNE, Paseo de Miramón 182, 20009 Donostia, San Sebastián, Spain.; 4 IMEM-CNR Institute of Materials for Electronics and Magnetism, Parco Area delle Scienze 37/A, I-43124 Parma, Italy.; 5 ICCOM-CNR Trieste Associate Unit, University of Trieste, via L. Giorgieri 1, 34127 Trieste, Italy.; 6 Ikerbasque, Basque Foundation for Science, 48013 Bilbao, Spain.

## Abstract

The favorable exploitation of carbon nitride (CN) materials in photocatalysis for organic synthesis requires the appropriate fine-tuning of the CN structure. Here, we present a deep investigation of the structure/activity relationship of CN in the photocatalytic perfluoroalkylation of organic compounds. Four types of CN bearing subtle structural differences were studied via conventional characterization techniques and innovative nuclear magnetic resonance (NMR) experiments, correlating the different structures with the fundamental mechanistic nexus and especially highlighting the importance of the halogen bond strength between the reagent and the catalyst surface. The optimum catalyst exhibited an excellent performance, with a very wide reaction scope, and could prominently trigger the model reaction using natural sunlight. The work lays a platform for establishing a new approach in the development of heterogeneous photocatalysts for organic synthesis related to medical, agricultural, and material chemistry.

## Teaser

In-depth analysis of structure/activity relationship aids rational design of modified carbon nitride for organophotocatalysis.

## INTRODUCTION

There is increasing pressure on industry for a rapid switch to new sustainable synthetic schemes to access chemicals of widespread use. In this context, heterogeneous photocatalysis by readily available, metal-free catalysts has a formidable appeal. In contrast with homogeneous systems, heterogeneous catalysts can be easily recycled; moreover, the metal-free nature avoids some typical drawbacks of metal-based catalysis such as: (i) high cost of the metal, (ii) poor compatibility with particular reaction environments, and (iii) decrease in activity over time due to nanoparticle aggregation. Graphitic carbon nitride (g-CN) is an ideal semiconductor nanomaterial candidate, with a relatively narrow bandgap, extensively used in photocatalytic applications related to energy, such as H_2_ production, water oxidation, and CO_2_ reduction (*1*–*3*). It is a stable material that can be prepared by simple and scalable procedures, and properties and reactivity can be fine-tuned by carrying out structural modifications (*4*). For instance, protonation and doping with heteroatoms other than N can adjust the electronic band edges and increase exposed surface area (*5*–*7*), while intercalation of metal ions can improve charge carriers mobility, decrease the bandgap, and provide the material with additional catalytic sites (*8*, *9*). In recent years, use of g-CN has been extended to photocatalytic organic synthesis for preparing industry-relevant compounds. While mainly used in photocatalytic oxidation of small molecules (*10*, *11*), there are also notable examples of C–C and C-heteroatom bond formation reactions (*12*–*15*), including the functionalization of arenes and heteroarenes with fluoroalkylated groups (*16*), which are important compounds in medicinal chemistry, agrochemistry, and material science (*17*–*19*). The use of g-CN circumvents that of catalysts such as Ru/Ir/Cu metal complexes (*20*–*22*), which are associated with high cost, toxicity, and nonrecyclability, or that of organic dye-based catalysts, which require very high catalyst loadings (*23*–*25*). Hence, the development of stable and cost-effective visible-light photocatalysts for C–C bond formation toward fluoroalkylated compounds is highly demanded. An in-depth analysis of the mechanistic features of the reaction and the consequent structure/activity relationship will establish the platform for the rational design of new and more effective material-based catalysts, for a range of different reactions. Here, diversely modified CN catalysts were prepared and investigated in the visible-light photocatalytic perfluoroalkylation of electron-rich organic substrates (**1**) by using simple perfluoroalkyl iodides (**2**) as the radical sources under mild operative conditions. The choice of **2** as model substrates lies in their high tendency to form reactive fluorinated radicals upon photo-induced single-electron transfer processes (*26*). The postsynthetic modifications afforded materials with fine-tuned properties, and a robust correlation between the structural features and the catalytic behavior was established by the combination of detailed characterization, advanced nuclear magnetic resonance (NMR) spectroscopy, and production rates. Moreover, the catalysis could be extended to many substrates, covering a wide reaction scope, including arenes and unsaturated aliphatic molecules. New insights were acquired into the specific and most relevant interactions between the CN materials and the used fluorinated substrates (**2**), which modulate kinetics and thermodynamics of the photocatalytic reaction. Last, the best-performing photocatalyst, with a very low loading, was successfully tested under Sun exposure at ambient temperature, demonstrating the excellent catalytic competence under real operative conditions.

## RESULTS

**Fig. 1:**
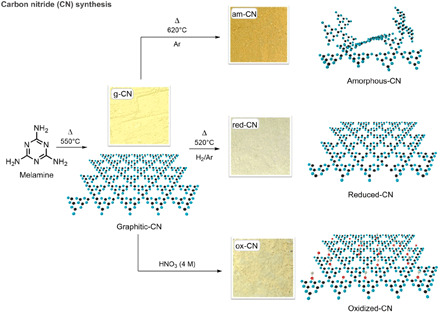
Sketch of synthesis and structure of the different CN materials. Graphical sketch of the synthesis of the various CN structures, with the associated photograph of the as-obtained powdery material. Standard thermal conditions applied to melamine lead to synthesis of g-CN, where the morphology is typically described by melem units connected in-plane; oxidative treatment presumably introduces small amounts of oxygenated functional groups on the surface (ox-CN), while reductive treatment partly removes N atoms, creating planar vacancies (red-CN); higher-temperature thermal treatment under inert atmosphere generates partially amorphous structure by misalignment of CN planar domains (am-CN). Note that the graphic rendering of the structure is only an idealized depiction used for the benefit of discussion. Real CN structures are much more complex. Photo credit: Francesco Longobardo, University of Trieste, Italy.

Three structural and chemical variations were introduced into the pristine g-CN, adopting a reduction protocol (red-CN), a mild oxidation protocol (ox-CN), and a thermal amorphization protocol (am-CN; Fig. 1). The chosen modifications introduce a specific feature in each material that was expected to reflect in a diverse interaction with the substrate, as described in detail below.

**Fig. 2:**
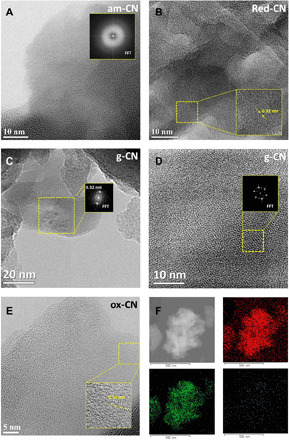
High-resolution microscopy of the four samples. (**A**) Representative HRTEM image of am-CN. Inset: FFT of a selected area showing the pattern of a typical amorphous material. (**B**) Representative HRTEM image of red-CN. Inset: High magnification of a selected area, showing the crystal lattice fringes, where a 0.32-nm interlayer spacing is measured. (**C**) Representative HRTEM of g-CN with the FFT (inset) showing spots assigned to the expected 0.32-nm interlayer spacing and (**D**) high magnification of a selected area of (C) with the FFT showing the intralayer XRD pattern with a 0.68-nm spacing. (**E**) Representative HRTEM image of ox-CN with the inset showing the lattice fringes with the 0.32-nm interlayer spacing and (**F**) EDX elemental mapping of ox-CN: carbon (red), nitrogen (green), and oxygen (blue).

**Fig. 3:**
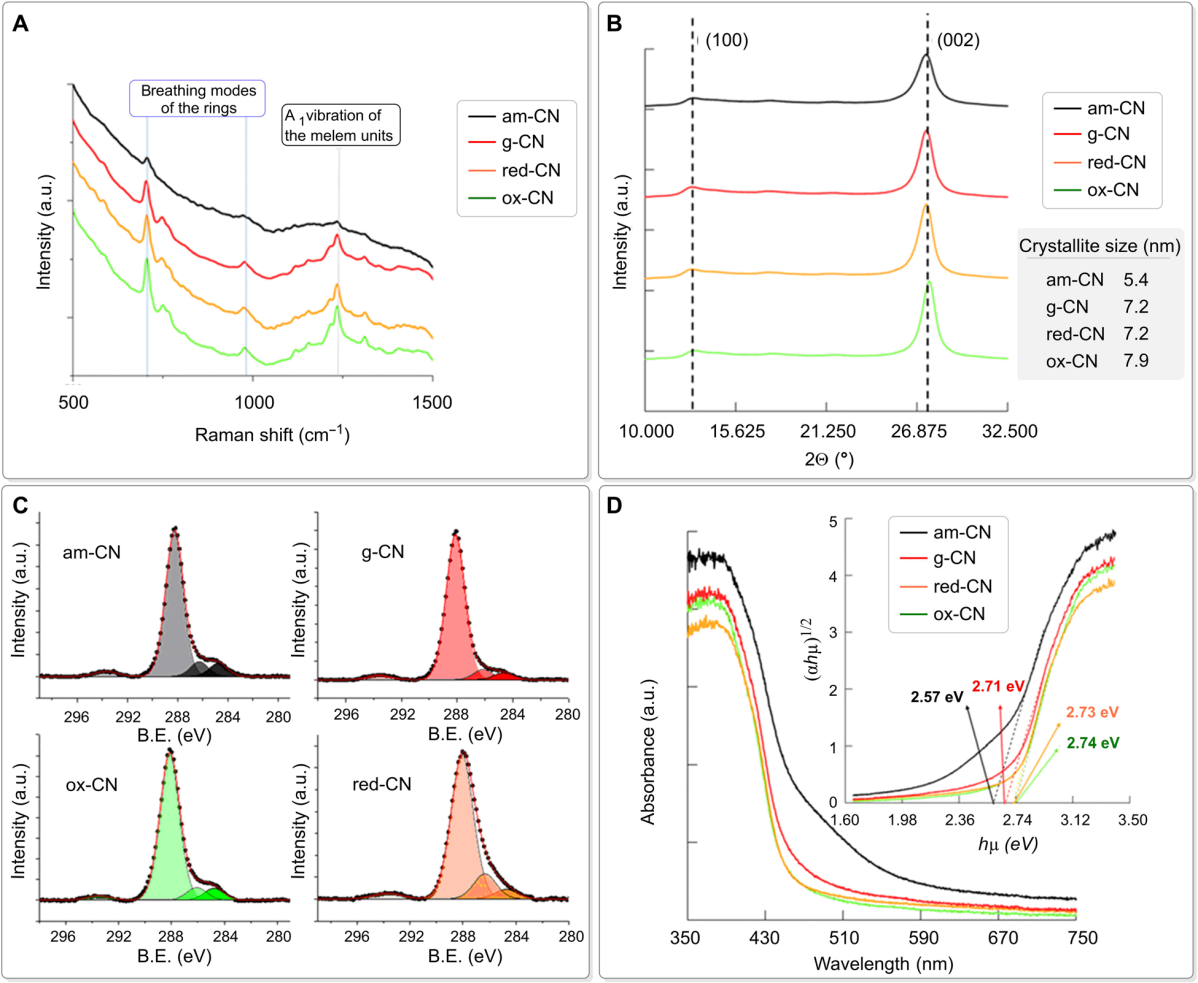
Physical characterization. Near-infrared (NIR) Raman spectra (**A**) and XRD diffractogram (**B**) of the four materials. Crystallite sizes reported in the table of (B) were calculated by applying the Scherrer equation to the (002) reflection. (**C**) High-resolution XPS spectra in the C1s binding energy (B.E.) range. a.u., arbitrary units. (**D**) DRS spectra and the corresponding band gap (inset).

Microscopic analysis through transmission electron microscopy (TEM) revealed that the three modifications result in slight morphological alterations, with the smooth sheet-like geometry of g-CN being partially scrambled (fig. S1). A visual inspection of the materials reveals a change in the color depending on the specific treatment, consequent to the structural change and, in turn, a change in the electronic properties of each CN (Fig. 1). In particular, H_2_/Ar high-temperature treatment (red-CN) has been reported to lead to defective structure introducing superficial nitrogen vacancies (*27*) confirmed here by x-ray photoelectron spectroscopy (XPS; vide infra). The absence of N atoms not only creates additional defects in the structure but also decreases the overall polarization of the C atoms on the surface of the catalyst. In contrast, the oxidation protocol (ox-CN) introduces additional amounts of oxygen atoms, as confirmed by XPS, presumably through formation of oxygenated functional groups on the surface, similarly to oxidation of other carbon nanostructures (*28*, *29*). In principle, the presence of these functional groups can be noninnocent, contributing to direct interaction with the substrate; alternatively, O atoms inserted onto the CN surface can behave as dopants and tune the electronic properties (*30*). Higher-temperature treatment under inert atmosphere (am-CN) is able to disrupt hydrogen bonding of the NH/NH_2_ groups, causing misalignment between strands of polymeric melon units with consequent partial amorphization and particle size reduction (am-CN) (*31*). High-resolution TEM (HRTEM) shows differences in the thermally treated materials (am-CN and red-CN) as compared with the conventional g-CN. In particular, the crystalline domains in the latter are more clearly identified and present in high density, with the lattice fringes being confirmed by fast Fourier transform (FFT) from which an interplanar spacing of 0.32 nm is seen in agreement with literature (Fig. 2C) (*32*). In contrast, for am-CN, the crystal fringes are not easily detected, with the observed dominant FFT pattern being associated to an amorphous material (Fig. 2A). Moreover, the FFT of g-CN reveals the order of the in-plane geometry, namely, the intralayer spacing of 0.68 nm is appreciated as observed in other reports (Fig. 2D) (*32*, *33*). This feature is not found in am-CN, presumably as the order is partly broken. For red-CN, which was treated under milder conditions than am-CN, we could observe some crystalline domains, although much less densely distributed in comparison with g-CN (Fig. 2B). Last, the most interesting feature for ox-CN, where the qualitative crystalline domains distribution look intermediate between am-CN and red-CN based on HRTEM inspection (Fig. 2E), is gained by energy-dispersive x-ray (EDX) spectroscopy elemental mapping, which shows that this is the only sample where oxygen is present in substantial percentage throughout the material (Fig. 2F). Two very sensitive techniques to confirm the disruption of the long-range order (“amorphization”) are x-ray powder diffraction (XRD) and Raman spectroscopy as also reported by Kang *et al.* previously (*31*). In particular, it can be observed for am-CN that the strong 27.2° peak ascribed to the (002) plane of the interlayer stacking of the conjugated aromatic system (*33*), associated with the crystallinity of the material, is broader and less intense as compared to the other three samples, in agreement with an increased amorphous nature (Fig. 3B). The calculated crystallite size (Fig. 3B, inset table) is therefore smaller (5.4 nm), implying a reduction in the number of layers in am-CN. Consistently, the intensity of the (100) reflection at 12.7°, which is associated to the intralayer spacing (also observed in the HRTEM), decreases from g-CN to am-CN, further corroborating the hypothesis of the partial rupture of the strands of heptazines. In agreement with this hypothesis, Raman spectroscopy (Fig. 3A) reveals the different profiles for the four materials. It is known that Raman spectroscopy of CN materials under visible excitation is problematic due to the strong fluorescence in this wavelength range (*34*). However, use of near-infrared (NIR) excitation provides an informative Raman pattern. NIR-Raman spectra of the four samples show the characteristic peaks at ~705 and 990 cm^−1^, due to breathing modes of the rings, and ~1200 cm^−1^, due to the A1 vibration of the melem units (Fig. 3A) (*35*, *36*). The lower peak intensity pattern in am-CN, as compared to the other three materials, is in line with the increased amorphous character as observed also in the XRD. Further evidence is lastly provided by the thermogravimetric analysis (TGA), where the combustion temperature is decreased by about 50°C in all treated CN with respect to that of the pristine material (fig. S2), implying a lesser crystallinity.

XPS shows that for all materials, the two main components C and N are in similar atomic ratios (see table S1), with red-CN bearing a lower content of N, as expected for a H_2_-treated CN material (*27*). In addition, the increment of C–N component (286.2 eV) of red-CN as compared to the other samples reflects the reduction process. All C1s spectra could be deconvoluted in four components, 284.8, 286.2, 288.2, and 293.5 eV, assigned to C–C, C–N, C═N–C, and π-π* species, respectively (*37*). Amorphization by higher-temperature treatment is reflected into the lower contribution of the aromatic component (288.2 eV) with respect to the other species, as compared to g-CN (Fig. 3C). The anticipated increase in oxygen content is lastly confirmed in ox-CN, where O atomic % reaches 5.3%. For all materials, high-resolution spectrum of the N1s core level could be deconvoluted into three different peaks, including the most intense component at 398.8 eV assigned to C═N–C nitrogen atoms in the sp^2^-hybridized nitrogen, a component N–(C)_3_ originating from the three-coordinated nitrogen atoms at 400.7 eV, and a minor contribution at around 404.5 eV, whose attribution is controversial and, in some cases, has been related to a N–N bonding (fig. S3) (*37*). XPS analysis also confirms the absence of metal species in detectable amounts. This is an important aspect in relation to the recent debate on the true “metal-free” nature of carbon nanostructures, which may contain metal impurities (*38*). While XPS provides information on the superficial element composition, the metal-free nature of the bulk catalysts was further proved by inductively coupled plasma optical emission spectrometry (ICP-OES) where metals were below the limit of detection. The materials were also characterized by elemental analysis to evaluate the differences in the bulk (table S2). As expected, the composition of all samples remains very similar to each other and in agreement with values reported for g-CN prepared from melamine precursor, where the C:N atomic ratio is ~0.66 due to residual uncondensed melamine (*32*, *39*, *40*). This confirms that the structural modifications are mainly involving the surface of the materials, which are those relevant for the catalysis as it is where adsorption of reagents occurs.

The bandgap results are affected by the postmodification of each sample, as observed by ultraviolet-visible (UV-vis) diffuse reflectance spectroscopy (DRS) analysis, which shows a different absorbance profile of am-CN, whose bandgap is reduced to 2.57 eV as compared to g-CN (2.71 eV). The oxidative and reductive treatments, in contrast, cause smaller variations, suggesting a lower disruption of the long-range order (2.73 eV for red-CN and 2.74 eV for ox-CN). It is also observed that the absorption profile for am-CN also present a broad peak at lower energy (larger wavelength), which could be associated to the presence of intragap states, as also previously suggested (Fig. 3D) (*41*). The valence band (VB) edge for the four materials was calculated by the analysis of the XPS profile in the low binding energy range (fig. S4), with values in the range expected from previous literature (*42*). Compared to the g-CN (VB = 1.83 eV), the thermal treatment carried out for preparing am-CN and red-CN decreases the energy of the VB, while the oxidative treatment increases it (fig. S5). As a result, the calculated conduction band (CB) edges for am-CN and red-CN are localized at more negative energies (−0.92 and −1.28 eV; fig. S5), thus being more efficient for reduction of the substrates, based on the reported redox potential ranges of the perfluoroalkyl iodides [about −0.76 V versus Reversible Hydrogen Electrode (RHE)] (*20*). In any case, the CBs of all samples are adequate for performing the reduction step on the fluorinated compounds, while it is insufficient for other nonfluorinated substrates that were tested (see below), whose redox potentials lie at significantly more negative energies, thus requiring future development of the CN catalysts, with ad hoc engineering of the band structure by alternative synthetic methodologies.

Structural distortions are accompanied by relatively small changes in surface area, with the Brunauer-Emmett-Teller (BET) surface area remaining in the range between 7 and 19 m^2^ g^−1^, namely, relatively low as expected for CN materials (fig. S6) (*43*). However, although in all cases, the isotherms and the *t*-plot analysis indicate presence of meso/macropores, the pore size distribution is affected, with the average pore diameter increasing in the order g-CN (maximum of distribution, 65 nm) < red-CN (maximum of distribution, 73 nm) < ox-CN (maximum of distribution, 78 nm) < am-CN (maximum of distribution, 122 nm; fig. S7). Larger pores in am-CN presumably minimize mass diffusion restrictions, contributing to increase the activity of this catalyst.

As a primary goal of the investigation, the relationship between the structure of the material and its photocatalytic activity was thoroughly investigated on a model reaction, to reveal the key parameters influencing activity. For this reason, 1,3,5-trimethoxy-benzene (**1a**) as the model electron-rich organic molecule and perfluorobutyl iodide (**2a**) as the fluorinated precursor were chosen (Table 1). Optimization of conditions was carried out with the pristine photocatalyst (g-CN), under 450-nm blue monochromatic irradiation at ambient temperature. Under the standard conditions (Table 1, entry 1), the studied transformation proceeds quantitatively to afford **3a** (yield, >99%) after 24 hours and with complete product selectivity. In contrast, deviations from standard conditions severely affect the reaction. In particular, absence of catalyst (Table 1, entry 2), absence of light (Table 1, entry 3), presence of oxygen (Table 1, entry 4), and presence of (2,2,6,6-tetramethylpiperidin-1-*yl*)oxyl (Table 1, entry 5) prevent the transformation, confirming both the photocatalytic and radical nature of the process. Other deviations from standard conditions such as the change of irradiation wavelength, as well as changes of base, reaction stoichiometry, and solvent, are also detrimental (Table 1, entries 6 to 11). The photocatalytic activity of the four different prepared materials was then screened. Reducing the amount of g-CN to half [0.35% (w/v)] results in a notable drop of yield (Table 1, entry 12). Disappointingly, the use of ox-CN leads to the formation of the product **3a** in very poor chemical yield (Table 1, entry 13). On the other hand, both red-CN and am-CN provide very high yield, 90 and 99%, respectively (Table 1, entries 14 and 15). The best reaction conditions were thus found as shown in entry 16 of Table 1. The amorphous catalyst is highly performant even at lower loadings, reaching a 89% yield at 0.125% (w/v), which is a very low loading as compared to typical reported conditions (Table 1, entry 17) (*16*).

**Table 1:** Catalytic tests on the model reaction. Optimization studies and control experiments. Reactions were performed on 0.1 mmol scale. TEMPO, (2,2,6,6-tetramethylpiperidin-1-*yl*)oxyl. Photo credit: Giacomo Filippini, University of Trieste, Italy. 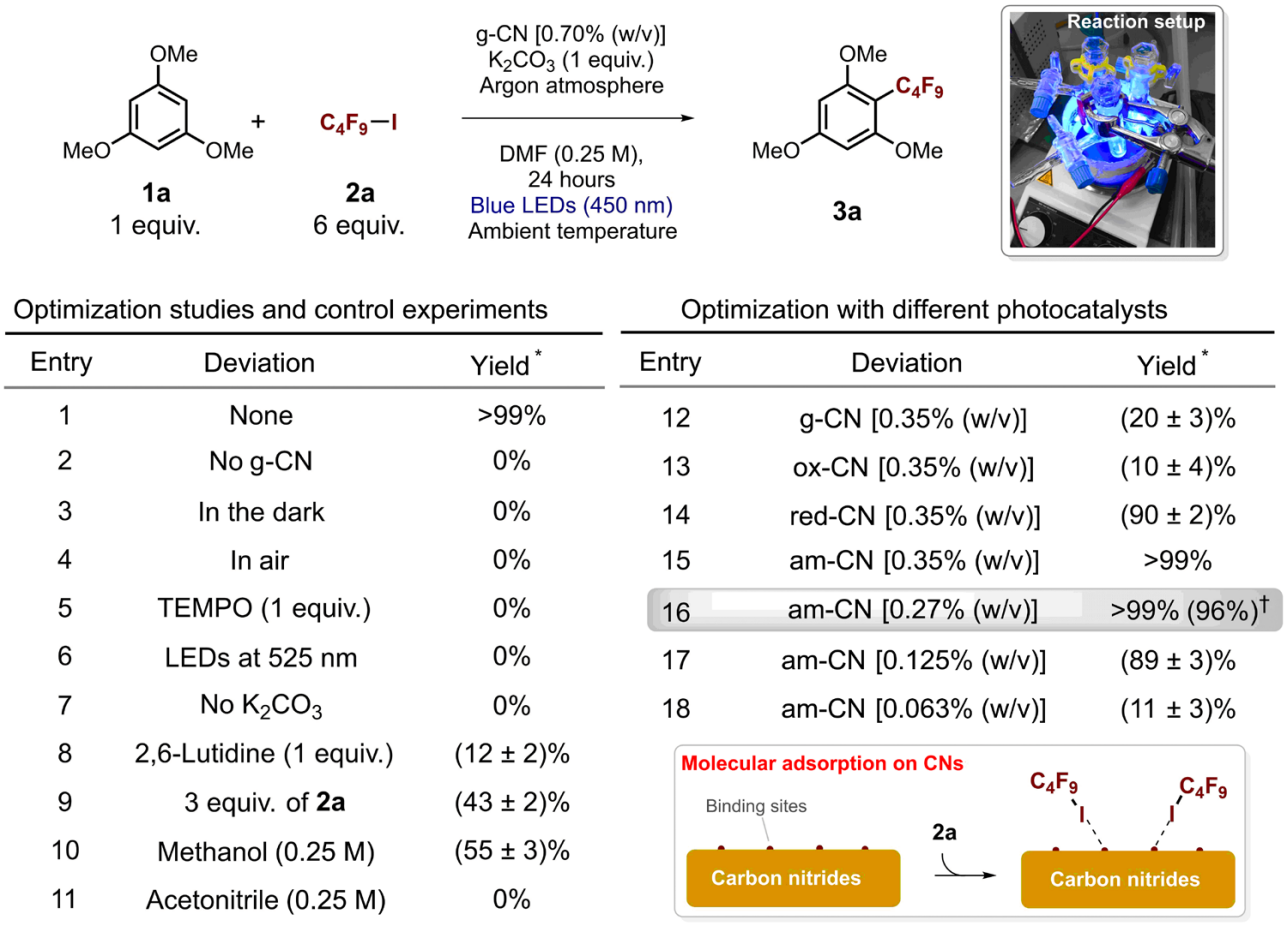

*Yield determined by ^1^H-NMR spectroscopy using 1,1,2-trichloroethene as the internal standard.

†Isolated yield.

The rates of product formation were calculated for the product **3a** with the four catalysts. Following very recent proposed guidelines for reporting the activity of photocatalysts (*44*), the rates were also calculated per surface area of the four catalytic materials (table S3), to evaluate the contribution of the geometric effect. It can be seen that over the 24-hour reaction time, if normalized by the surface area, the red-CN catalyst is apparently more efficient, hinting that intrinsically, such a catalyst is more active. However, it must be noted that red-CN after 24 hours is unable to convert totally the reagent, while am-CN does. If normalized per mass of catalyst, am-CN is the most efficient.

**Fig. 4:**
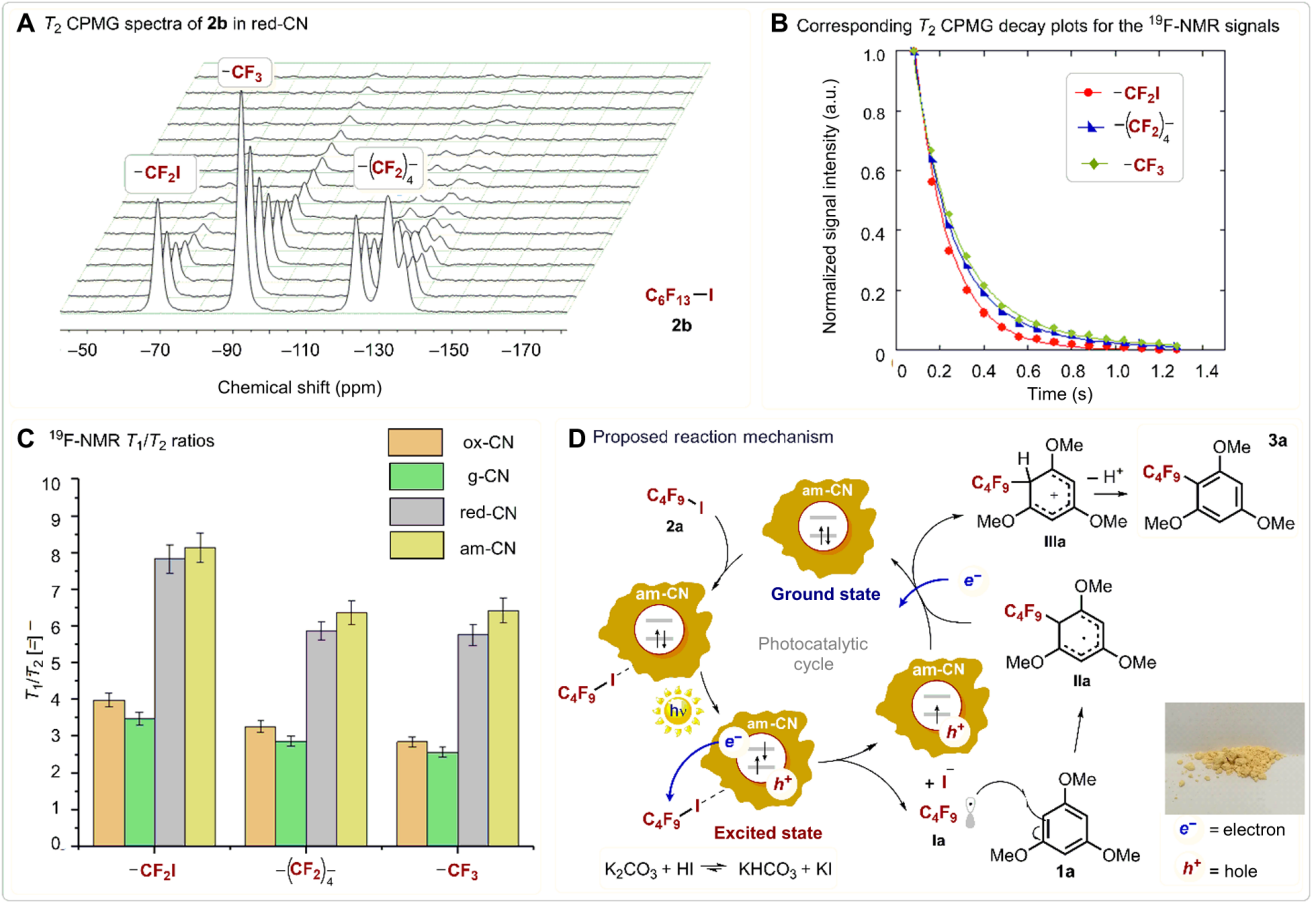
NMR investigation and proposed mechanism. (**A**) *T*_2_ CPMG spectra of perfluorohexyl iodide in red-CN. The CF_2_I resonance is at approximately −69 parts per million (ppm), that of the CF_3_ at approximately −92 ppm, and the (CF_2_)_4_ peaks in the range of −120 to −145 ppm. (**B**) Corresponding *T*_2_ CPMG decay plots for the NMR signals of CF_2_I, (CF_2_)_4_ and CF_3_ fitted using a single CPMG exponential decay. (**C**) ^19^F NMR *T*_1_/*T*_2_ ratio of the different moieties of perfluorohexyl iodide in the various CN-based photocatalytic materials used in this work. (**D**) Proposed reaction mechanism that drives the photocatalytic perfluorobutylation of **1a**. The perfluorinated substrate binds the catalyst surface via iodine halogen bond. After photoexcitation and charge separation in the semiconducting catalyst, excited electrons are injected from the catalyst to the substrate, forming the radical **Ia**. The radical then attacks the aromatic molecule, leading to the cascade reaction generating the final product. Photo credit: Francesco Longobardo, University of Trieste, Italy.

A plausible mechanism that drives the catalytic reaction by the am-CN is proposed on the basis of previous findings and supported by NMR studies (see below). Figure 4D shows the catalytic cycle, where before photo-induced charge separation in the semiconductor, the binding of **2a** occurs presumably via halogen bonding with the N atoms of the photocatalyst (*45*). This step is of high importance, as it markedly affects the rate of the injection of the photo-excited electron into the C_4_F_9_I reagent and, thus, also the formation of the ∙C_4_F_9_ radical (**Ia**). Lastly, the as-formed open-shell species **Ia** attacks the aromatic ring of the trimethoxy-benzene (**2a**), and the catalysis eventually evolves to the final desired product (**3a**). We also evaluated the apparent quantum yield (AQY) of reaction producing **3a** by am-CN, to gain insights into the possibility of a chain-propagating mechanism, for which the quantum yield is expected to be >1, as discussed in recent reports on photocatalysis mechanisms (*46*). As the AQY is of 0.016 mmol (**3a**) per millimole of photons, therefore <1, the reaction does not presumably occur via a chain-propagation process, although this cannot be totally ruled out as the AQY < 1 may also derive from a highly inefficient initiation step (*46*).

Assessment of the iodine halogen bond strength and its effect on reactivity was investigated by ^19^F NMR *T*_1_/*T*_2_ relaxation measurements using perfluorohexyl iodide (**2b**) as our probe molecule (we chose a longer-alkyl chain substrate due to the lower volatility than **2a**). The *T*_1_/*T*_2_ ratio has been shown to be a robust indicator of strength of surface interactions of liquids confined in porous catalysts (*47*). It has previously been demonstrated that these measurements allow the quantification of the strength of hydrogen bonding of liquids confined in porous catalysts, as reported by some of us who studied the behavior of alcohols inside porous silica, using ^1^H NMR relaxation (*48*, *49*). Prompted by these results, in this work, we have extended the methodology to probe the strength of surface interactions of the perfluoroalkyl iodide reagent over the different catalysts screened for the reactions, and exploiting ^19^F NMR. *T*_1_ was measured using the inversion recovery pulse sequence, while *T*_2_ was measured using the Carr-Purcell-Meiboom-Gill (CPMG) pulse sequence. Figure 4A contains a typical dataset, which shows the *T*_2_ CPMG decay of the perfluoroalkyl iodide compound inside the red-CN sample. Figure 4B shows the *T*_2_ CPMG decay of CF_2_I, (CF_2_)_4_ and CF_3_, from which it can be seen that within the same molecule, the various groups have different *T*_2_ CPMG decay rates, with the resonance of the CF_2_I having a significantly faster decay compared to the (CF_2_)_4_ and CF_3_.

## DISCUSSION

Two important observations can be drawn from the data in table S4 and Fig. 4C. First, in all cases, the *T*_1_/*T*_2_ of the CF_2_I moiety of perfluorohexyl iodide is higher compared to those of the (CF_2_)_4_ and CF_3_ within the same molecule. In particular, the following trend, consistent across all materials, can be observed

$$T_{1}/T_{2}[{\text{CF}}_{2}I]>T_{1}/T_{2}[{\left({\text{CF}}_{2}\right)}_{4}]≃T_{1}/T_{2}[{\text{CF}}_{3}]$$

This indicates that the strength of surface interaction of the CF_2_I is greater compared to the rest of the molecule, and groups further away from this moiety exhibit a weaker interaction. The presence of van der Waals dipole-dipole interactions with the surface of CN does not explain this trend as these forces are present for all moieties and should be even stronger for fluorine-richer moieties, such as (CF_2_)_4_ and CF_3_. This suggests that it is the iodine atom that differentiates the CF_2_I moiety, especially if it is considered that the strength of halogen bonding is the highest for iodine as compared with the other halogens (*50*). Hence, the current results and the previously published work (*48*) strongly support the hypothesis that the ^19^F *T*_1_/*T*_2_ trend observed in this work can be ascribed to the ability of the CF_2_I to form halogen bonding with the solid surface of the catalyst, giving exclusive new insights into adsorbate/adsorbent interactions in these materials. Further insights into the relaxation behavior can be drawn by calculation of surface relaxivities (see the Supplementary Materials for details), that provide more evidence on the catalytic trend.

Second, by looking at the *T*_1_/*T*_2_ of the CF_2_I group of perfluorohexyl iodide in different catalysts, the following trend can be identified

$$T_{1}/T_{2}[\text{am-CN}]>T_{1}/T_{2}[\text{red-CN}]>T_{1}/T_{2}[\text{ox-CN}]≃T_{1}/T_{2}[\text{g-CN}]$$

The trend exactly parallels with the reactivity trend in Table 1, indicating that materials with a higher surface affinity for the fluorinated substrate are also those with the highest activity. This strongly suggests that the ability of the perfluoroalkyl iodide to form halogen bonding with the solid material is a critical factor determining reactivity and support the reaction mechanisms proposed in Fig. 4C, highlighting the importance of the binding of the perfluoroalkyl iodide to the catalyst surface. ^1^H NMR *T*_1_/*T*_2_ relaxation measurements using the reaction solvent dimethylformamide (DMF) were carried out to evaluate the contribution of the solvent affinity to the reaction (figs. S8 and S9). From these data, it is possible to identify a clear trend in *T*_1_/*T*_2_ ratio value for DMF imbibed within the pores of the various photocatalysts

$$T_{1}/T_{2}[\text{ox-CN}]>T_{1}/T_{2}[\text{g-CN}]>T_{1}/T_{2}[\text{red-CN}]>T_{1}/T_{2}[\text{am-CN}]$$

As noted, the trend is inverse to that of activity, which suggests that when the DMF solvent has a lower surface interaction with the catalyst surface, it can be easily displaced by the reagent, hence preventing the blocking of catalytically active sites (figs. S8 and S9 and table S5; also see discussion therein). The advanced NMR studies therefore shed important new light on the structure/activity relationship in CN materials, as connected with the microstructure of the specific sample. In particular, the NMR results indicate that the stronger surface affinity of the perfluoroalkyl iodide for the catalyst relative to that of the solvent is critical for controlling the access to the active sites of the substrate. This has been previously observed for liquid-phase oxidation over heterogeneous catalysts (*51*).

As mentioned above, other parameters are, however, important for defining the overall catalytic performance. In particular, the reduced bandgap of am-CN resulting in its better absorption at 450 nm could contribute significantly. Quantitatively discerning the contribution of the different parameters is extremely complicated. However, to confirm that optical properties are not the unique cause of the higher performance of am-CN, catalytic experiments were carried out on the four materials with a higher-energy light-emitting diode (395 nm corresponding to 3.14 eV) on the model reaction (table S6). Instead of a flattening of the activity among the four photocatalysts following saturation of the electron/hole separation process with such a higher-energy light source, we still observed a clear difference in the activity trend, which remains almost unaltered. This rules out a sole effect on the performance due to CB population differences but strongly suggests that other factors are involved, although the reduced bandgap in am-CN does play a role.

**Fig. 5:**
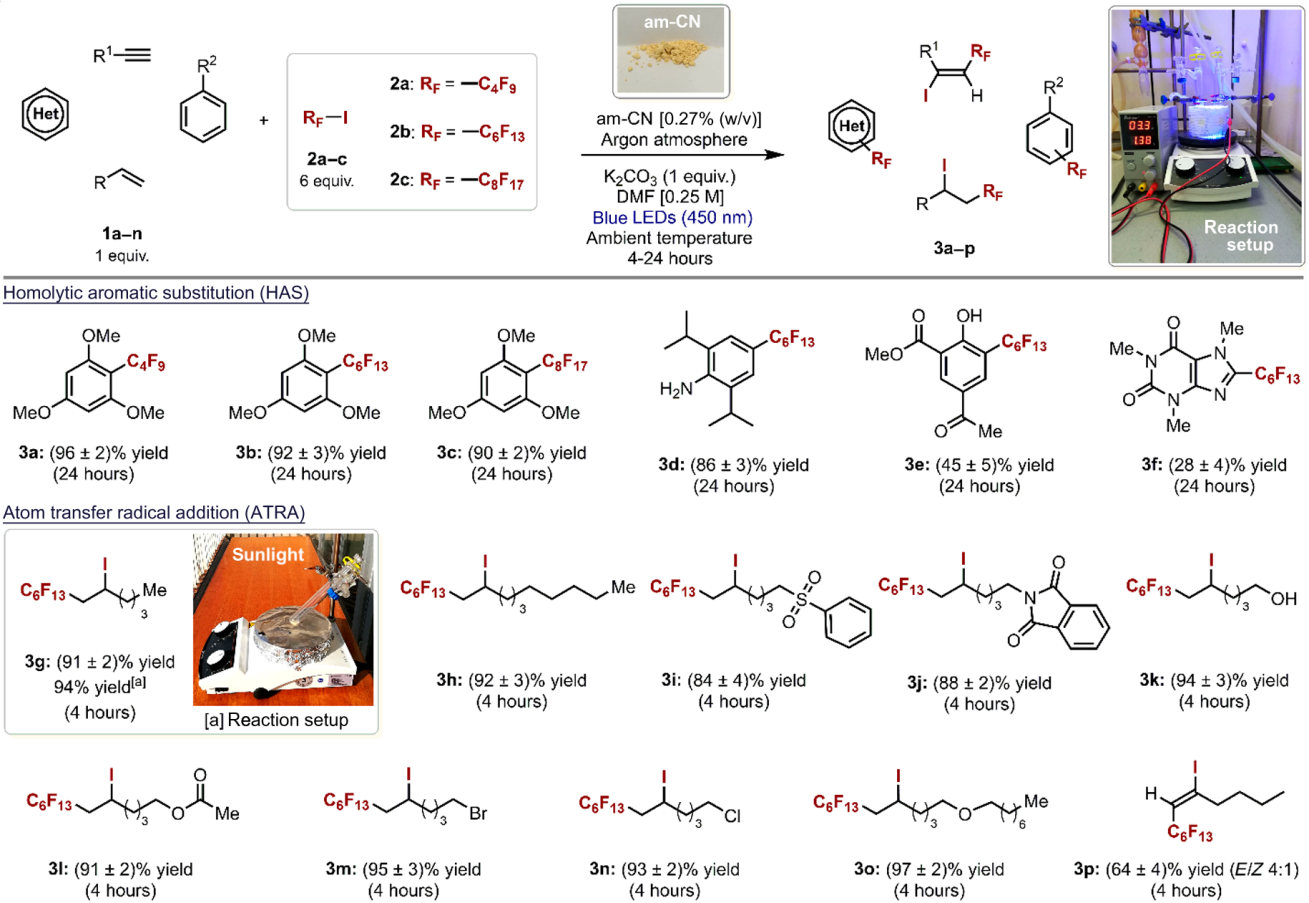
Evaluation of the scope of the photochemical reaction. Survey of the organic compounds and perfluoroalkyl iodides that can participate in the photocatalytic process. Conditions: Reactions are conducted in Schlenk tubes in DMF (0.25 M) on 0.1 mmol scale of **1**, 0.6 mmol of perfluoroalkyl iodide **2**, 0.1 mmol of K_2_CO_3_, and 0.27% (w/v) of am-CN, degassed by four freeze-pump-thaw cycles and irradiated for 4 to 24 hours by a blue light-emitting diode (LED) strip (450 nm). [a] Outdoor experiment and the used setup (from 9:00 to 13:00 of 5 August 2019, in Trieste; see fig. S10). Photo credit: Giacomo Filippini, University of Trieste, Italy.

The scope of the reaction was then investigated using the am-CN [0.27% (w/v)] as photocatalyst under the optimized operative conditions (Fig. 5), and functionalization of a variety of aromatic, heteroaromatic, and unsaturated aliphatic substrates (**1a** to **1n**), even bearing reaction-sensitive functional groups, was successfully achieved, confirming the high tolerance of the photocatalyst (products **3a** to **3p**, isolated yield of up to 97%). Catalytic tests using natural sunlight (product **3g**) also proved successful, and the reaction was completed within 4 hours in analogy with the artificial illumination test on the same alkene substrate (**1e**). This is an essential aspect for future real applications of am-CN as photocatalyst in organic synthesis. The stability of the am-CN photocatalyst was evaluated by recycling the catalyst three times at the end of the reaction for the product **3g**, and we did not observe any substantial decrease in activity, the small differences being due to the physical loss of the am-CN material during separation from the reaction environment (fig. S11).

To the best of our knowledge, the investigated reactions have been exclusively performed with homogeneous photocatalysts, so that our example is the first with a heterogeneous catalyst. Nevertheless, we compared the performance versus homogeneous systems (table S7). Given the large scope of reaction investigated herein, we selected the product **3k** as a representative case study, given that it is the most represented in literature. It can be seen from the rate of production of **3k** that despite the intrinsically general higher activity of homogeneous catalysts, am-CN performs better than most of the reported catalysts. This result, combined with the metal-free nature of am-CN and with the advantages of heterogeneous catalysis, highlights the great appeal of the present material and especially the synthetic approach for fine-tuning activity, which can be subject to future developments.

Last, we explored the generality of the catalytic behavior using a less electron-rich substrate, anisole, and observed a marked decrease in activity (overall yield, 25%) and selectivity (NMR shows presence of a complex mixture of adducts, presumably associated with different regio-isomers or bifunctionalization; table S8). The poor regio-selectivity is expected as it is typically associated with reactions proceeding with the homolytic aromatic substitution (HAS) mechanism (*52*). Other simple molecules such as benzene or naphthalene did not exhibit any reactivity, as also expected on the basis of the absence of strongly electron-donating groups on the substrate (table S8). Moreover, nonterminal alkenes such as cyclohexene displayed a drop of yield (19%) presumably to steric effects, together with a low diastereoselectivity (2:1 trans:cis isomers were formed; table S8). Equally, the reaction with nonperfluorinated hydrocarbons fails to proceed (table S9), as expected from considerations on the band structure of the semiconductor photocatalysts, located at energies suitable to convert reagents with a redox potential within the bandgap. The absence of the fluorine groups implies a notable shift of the redox potential toward much more negative values, out of the reduction ability of the described photocatalysts.

In conclusion, we present an in-depth experimental analysis of the photocatalytic activity for the perfluoroalkylation of electron-rich organic substrates by carbon nitride materials in relation to their different microstructure. A discrimination of the critical parameters that define activity is carried out through a combined set of investigations based on different characterization techniques. In particular, advanced ^19^F NMR provides key quantitative insights into the importance of reagent affinity toward the solid catalyst through formation of halogen bonding, which, in turn, depends on the local structure. This NMR approach could therefore be extended to other types of heterogeneous catalytic reactions involving halogen bonding. Our proof-of-concept study is lastly condensed into a practical exploitation of the best catalyst by using natural solar light for achieving high activity synthesis of a perfluorinated alkyl iodide. While focusing on pefluoroalkyl compounds, the work has the potential to inspire future rational design of CN-based photocatalysts for other organic reactions, provided that the CN catalyst is tailored with the suitable structural and electronic properties.

## MATERIALS AND METHODS

All reactions were set up under an argon atmosphere in Schlenk tubes, unless otherwise stated. Synthesis grade and anhydrous solvents were used as purchased. The catalysts were all grinded in a mortar for a period of 15 min before photocatalytic experiments, after which time thinly powdery samples are obtained. Chromatographic purification of products was accomplished using flash chromatography on silica gel (35 to 70 mesh). For thin-layer chromatography (TLC) analysis throughout this work, Merck precoated TLC plates (silica gel 60 GF254, 0.25 mm) were used, using UV light as the visualizing agent (254 nm), basic aqueous potassium permanganate (KMnO_4_) stain solution or iodine, and heat as developing agents. Organic solutions were concentrated under reduced pressure on a Büchi rotatory evaporator.

TGA was performed on a TGA Q500 (TA Instruments) in air. The samples were equilibrated at 100°C for 20 min, following a ramp of 10°C/min up to 830°C. Raman spectra were acquired using an Invia Renishaw spectrometer equipped with a diode laser at 785 nm. Diffuse reflectance UV-vis spectroscopy was performed with a Thermo Fisher Scientific Evolution 600 spectrophotometer, equipped with the Diffuse Reflectance Accessory Praying Mantis Sampling Kit (Harrick Scientific Products, USA). UV-vis spectra for starting materials were performed with Shimatzu UV-2450. XPS for all nanomaterials was performed with a SPECS Sage HR 100 spectrometer with a nonmonochromatic x-ray source of magnesium with a Kα line of 1253.6-eV energy and 250 W. XPS data were fitted using CasaXPS software. Standard TEM images were carried out on TEM Philips EM208, using an acceleration voltage of 100 kV. HRTEM characterization was performed using a JEOL 2200FS microscope operating at 200 kV, equipped with an EDX and a high-angle annular dark-field detector. To minimize the radiation damage from the electron beam, the HRTEM images were acquired using a very low beam current and low exposure time.

XRD was performed on a Philips X’Pert diffractometer using a monochromatized Cu Kα (λ = 0.154 nm) x-ray source in the range of 20° < 2θ < 100. N_2_ physisorption was performed with a Micrometrics ASAP 2020 analyzer at liquid nitrogen temperature. All the nanomaterials were degassed at 150°C for 12 hours at 10 μmHg. The specific surface area was calculated by applying the BET method equation. Pore size distributions were determined to the adsorption branch of the isotherms with Barrett, Joyner, Halenda (BJH) method equation.

### Materials

Commercial reagents and solvents were purchased at Sigma-Aldrich, Fluka, Alfa Aesar, Fluorochem, and VWR and used as received, without further purification, unless otherwise stated. 1,3,5-Trimethoxybenzene (**1a**), 2,6-diisopropylaniline (**1b**), methyl 5-acetylsalicylate (**1c**), caffeine (**1d**), 1-hexene (**1e**), 1-decen (**1f**), 5-hexen-1-ol (**1i**), 6-bromo-1-hexene (**1k**), 6-chloro-1-hexene (**1l**), 1-hexyne (**1n**), nonafluoro-1-iodobutane (**2a**), perfluorohexyl iodide (**2b**), heptadecafluoro-1-iodooctane (**2c**), 2,6-lutidine, melamine, and potassium carbonate are all commercially available. The preparation of olefins **1g**, **1h**, **1j**, and **1m** is detailed in the Supplementary Materials.

### ^1^H and ^19^F NMR relaxation measurements

NMR experiments were performed on a ^1^H/^19^F Magritek SpinSolve 43-MHz benchtop NMR spectrometer. For ^19^F NMR measurements, a pulse length of 130.4 μs was used, with a pulse amplitude of −6 dB for the 90° pulse and 0 dB for the 180° pulse, a receiver gain of 52, and acquiring 8192 points in the time domain with a dwell time of 50 μs. For ^1^H NMR measurements, a pulse length of 18 μs was used, while the other acquisition parameters were the same as those used for the ^19^F NMR experiments. *T*_1_ was measured using the inversion recovery pulse sequence (*53*), with a repetition time of 5 × *T*_1_, acquiring 16 time delay steps logarithmically spaced and a number of scans varying between 8 and 16 for ^1^H NMR experiments and between 32 and 64 for ^19^F NMR experiments, depending on the signal-to-noise ratio of the sample. *T*_2_ was measured using the CPMG pulse sequence (*53*) using an echo time of 1 ms with 16 steps, using two echoes per step and 16 to 32 scans for ^1^H NMR experiments and 128 to 1024 scans for ^19^F NMR experiments. To prepare solid samples for the NMR experiments, the photocatalyst solid particles were soaked in the liquid under investigation for 24 hours to ensure full saturation of the solid. The saturated solid samples were then transferred to 5-mm NMR tubes. To minimize errors due to evaporation of the liquid, a small amount of pure liquid was dropped onto a filter paper, which was placed under the cap of the NMR tube. The NMR tube was then placed into the magnet and left for approximately 20 min to achieve thermal equilibrium before measurements started.

### Synthesis of nanomaterials and characterization

All nanomaterials were prepared using commercial reagents and solvents were purchased at Sigma-Aldrich and used as received, without further purifications. For the g-CN, the furnace used was a cubic muffle operating under static air atmosphere; the samples were positioned in the middle. For the am-CN and red-CN, the furnace used was tubular, with the cylindrical quartz reactor inserted horizontally. The gas stream was inlet from one side of the reactor and exit to the other, with high-vacuum glass connectors. The gas mixture and flux were set, used, and calibrated by mass flow controllers.

#### g-CN

Ten grams of melamine was transferred in a covered alumina crucible and heated in muffle furnace at 550°C for 300 min with a ramping time of 5°C/min. The final product was milled to have a uniform powder.

#### red-CN

A total of 0.75 g of g-CN was transferred in an alumina boat-shaped crucible and thermally heated in a tubular furnace at 520°C for 120 min with a ramping time of 2°C/min in a mixture of argon (200 ml/min) and hydrogen (30 ml/min) flow.

#### am-CN

A total of 0.75 g of g-CN was transferred in an alumina boat-shaped crucible and thermally heated in a tubular furnace at 620°C for 120 min with a ramping time of 2°C/min in argon flow.

#### ox-CN preparation

A total of 0.75 g of g-CN was transferred in a 600-ml distillation flask, dispersed in 300 ml of a solution (4 M nitric acid), and sonicated for 5 hours. The product was collected by filtration and washed with 250 ml of MilliQ and 250 ml of methanol.

## Supplementary Material

http://advances.sciencemag.org/cgi/content/full/6/46/eabc9923/DC1

Adobe PDF - abc9923_SM.pdf

Light-driven, heterogeneous organocatalysts for C–C bond formation toward valuable perfluoroalkylated intermediates

## SUPPLEMENTARY MATERIALS

Supplementary material for this article is available at http://advances.sciencemag.org/cgi/content/full/6/46/eabc9923/DC1
