# Supplementary material for: Light-driven, heterogeneous organocatalysts for C–C bond formation toward valuable perfluoroalkylated intermediates
Source: Sci Adv. 2020 Nov 11;6(46):eabc9923. doi: 10.1126/sciadv.abc9923 (PMC7673726; doi:10.1126/sciadv.abc9923)
Supplement: http://advances.sciencemag.org/cgi/content/full/6/46/eabc9923/DC1 [file supp_6_46_eabc9923__index.html]

Science Advances | Science AdvancesAAASSearchScience AdvancesMenu

## Supplementary Materials

# Light-driven, heterogeneous organocatalysts for C–C bond formation toward valuable perfluoroalkylated intermediates

Giacomo Filippini, Francesco Longobardo, Luke Forster, Alejandro Criado, Graziano Di Carmine, Lucia Nasi, Carmine D’Agostino, Michele Melchionna, Paolo Fornasiero, Maurizio Prato

Download Supplement

**This PDF file includes:**

- Supplementary Text
- Scheme S1
- Figs. S1 to S12
- Tables S1 to S9
- References

**Files in this Data Supplement:**

- Adobe PDF - abc9923\_SM.pdf
