# Supplementary material for: Light-driven, heterogeneous organocatalysts for C–C bond formation toward valuable perfluoroalkylated intermediates
Source: Sci Adv. 2020 Nov 11;6(46):eabc9923. doi: 10.1126/sciadv.abc9923 (PMC7673726; doi:10.1126/sciadv.abc9923)
Supplement: http://advances.sciencemag.org/cgi/content/full/6/46/eabc9923/DC1 [file supp_6_46_eabc9923__1.pdf]

Science Advances
AAAS

advances.sciencemag.org/cgi/content/full/6/46/eabc9923/DC1

Supplementary Materials for

Light-driven, heterogeneous organocatalysts for novel C–C bond formation toward valuable perfluoroalkylated intermediates

Giacomo Filippini, Francesco Longobardo, Luke Forster, Alejandro Criado, Graziano Di Carmine, Lucia Nasi, Carmine D'Agostino, Michele Melchionna*, Paolo Fornasiero*, Maurizio Prato*

*Corresponding author. Email: melchionnam@units.it (M.M.); pfornasiero@units.it (P.F.); prato@units.it (M.P.)

Published 11 November 2020, *Sci. Adv.* **6**, eabc9923 (2020)
DOI: 10.1126/sciadv.abc9923

This PDF file includes:

Supplementary Text
Scheme S1
Figs. S1 to S12
Tables S1 to S9
References

Synthesis of Alkenes 1g, 1h, 1j and 1m

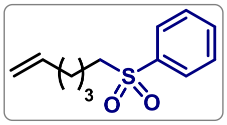

(Hex-5-en-1-ylsulfonyl)benzene (1g). *Prepared according to the literature procedure*.(*54*) In a two neck round-bottomed flask, purged under argon, a mixture of 6-bromo-1-hexene **1k** (260 µL, 2 mmol, 1 equiv.), sodium benzenesulfinate (391 mg, 2.4 mmol, 1.2 equiv.) tetrabutylammonium iodide (74 mg, 0.2 mmol, 0.1 equiv.) in dry DMF (2 mL) was heated up to 60°C and stirred over 5 hours. The reaction was quenched by the addition of brine (5 mL) and then extracted with ethyl acetate (3 x 5 mL). The organic phases were combined and washed with brine (5 mL) and then dried over sodium sulfate. The solvent was removed under reduced pressure and the residue was purified by flash column chromatography (*n*-hexane/ethyl acetate 9:1) to give the corresponding alkene **1g** as a colorless oil (372 mg, 83% yield). The characterization of the compound matches with the data reported in the literature.(*54*) ^{1}H-NMR (400 MHz, $CDCl_3$) δ 7.99 – 7.85 (m, 2H), 7.73 – 7.61 (m, 1H), 7.62 – 7.53 (m, 2H), 5.82 – 5.60 (m, 1H), 5.04 – 4.89 (m, 2H), 3.17 – 3.01 (m, 2H), 2.10 – 1.96 (m, 2H), 1.81 – 1.66 (m, 2H), 1.55 – 1.36 (m, 2H); HRMS calculated for $C_{12}H_{16}O_2S$ (M-Na): 247.0769, found: 247.0766.

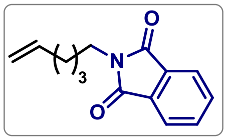

2-(hex-5-en-1-yl)isoindoline-1,3-dione (1h). *Prepared according to the literature procedure*.(*54*) In a two neck round-bottomed flask, purged under argon, a mixture of 6-chloro-1-hexene **1l** (400 µL, 3 mmol, 1 equiv.), potassium phthalimide (610 mg, 3.3 mmol, 1.1 equiv.) and potassium iodide (50 mg, 0.3 mmol, 0.1 equiv.) in dry DMF (5 mL) was heated up to 90°C and stirred overnight. The reaction was quenched by the addition of water (10 mL) and then extracted with dichloromethane (3 x 10 mL). The organic phases were combined and washed with KOH 0.2 M (10 mL), brine (10 mL) and then dried over sodium sulfate. The solvent was removed under reduced pressure and the residue was purified by flash column chromatography (n-hexane/ethyl acetate 9:1) to give the corresponding alkene **1h** as a pale yellow oil (506 mg, 74% yield). The characterization of the compound matches with the data reported in the literature.(*54*) ^{1}H-NMR (400 MHz, $CDCl_3$) δ 7.84 (dd, J = 5.5, 3.0 Hz, 2H), 7.70 (dd, J = 5.4, 3.1 Hz, 2H), 5.78 (ddt, J = 16.9, 10.2, 6.7 Hz, 1H), 5.04 – 4.91 (m, 2H), 3.69 (t, J = 7.3 Hz, 2H), 2.10 (dd, J = 14.3, 7.2 Hz, 2H), 1.75 – 1.65 (m, 2H), 1.44 (dt, J = 15.2, 7.5 Hz, 2H); HRMS calculated for $C_{14}H_{15}NO_2$ (M-Na): 252.1000, found: 252.0992.

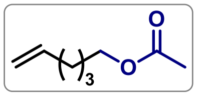

Hex-5-en-1-yl acetate (1j). *Prepared according to the literature procedure*.(*54*) In a two neck round-bottomed flask, purged under argon, a solution of 5-hexen-1-ol **1i** (420 µL, 3.5 mmol, 1 equiv.) and 2,6-lutidine (408 µL, 3.5 mmol, 1 equiv.) in dry THF (10 mL) was stirred at 0°C for 10 minutes. Acetyl bromide (260 µL, 3.5 mmol, 1 equiv.) was added dropwise and the solution was stirred at room temperature over 3 hours. The reaction was quenched by the addition of water (10 mL) and then extracted with diethyl ether (3 x 10 mL). The organic phases were combined and washed with brine (10 mL) and then dried over sodium sulfate. The solvent was removed under reduced pressure to give the corresponding alkene **1j** as a pale yellow oil (141 mg, 28% yield). The characterization of the compound matches with the data reported in the literature.(*54*) ^{1}H-NMR (400 MHz, $CDCl_3$) δ 5.80 (ddt, J = 16.9, 10.2, 6.7 Hz, 1H), 5.08 – 4.87 (m, 2H), 4.06 (t, J = 6.7 Hz, 2H), 2.19 – 1.99 (m, 5H), 1.73 – 1.56 (m, 2H), 1.54 – 1.37 (m, 2H); HRMS calculated for $C_8H_{14}O_2$ (M-Na): 165.0892, found: 165.0885.

1-(hex-5-en-1-yloxy)octane (1m). *Prepared according to the literature procedure*.(*54*) In a two neck round-bottomed flask, purged under argon, a solution of 5-hexen-1-ol **1i** (420 µL, 3.5 mmol, 1 equiv.) and 1-bromooctane (610 µL, 3.5 mmol, 1 equiv.) in dry THF (14 mL) was stirred at 0°C for 10 minutes. Sodium hydride (170 mg, 4.2 mmol, 1.2 equiv.) was added portionwise and the solution was heated up to reflux and stirred overnight. The reaction was quenched by the addition of ammonium chloride (10 mL) and then extracted with ethyl acetate (3 x 10 mL). The organic phases were combined and washed with brine (10 mL) and then dried over sodium sulfate. The solvent was removed under reduced pressure and the residue was purified by flash column chromatography (*n*-hexane/ethyl acetate 95:5) to give the corresponding alkene **1m** as a pale yellow oil (494 mg, 67% yield). The characterization of the compound matches with the data reported in the literature.(*54*) ^{1}H-NMR (400 MHz, $CDCl_3$) δ 5.79 (ddt, J = 16.9, 10.2, 6.7 Hz, 1H), 5.06–4.89 (m, 2H), 3.38 (td, J = 6.6, 4.9 Hz, 4H), 2.12–2.01 (m, 2H), 1.56 (dp, J = 9.6, 6.8 Hz, 4H), 1.49–1.38 (m, 2H), 1.38–1.22 (m, 10H); HRMS calculated for $C_{14}H_{28}O$ (M-Na): 235.2038, found: 235.2032.

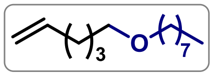

General Procedures for the Photocatalytic Fluoroalkylation of Organic Compounds and Characterization Data

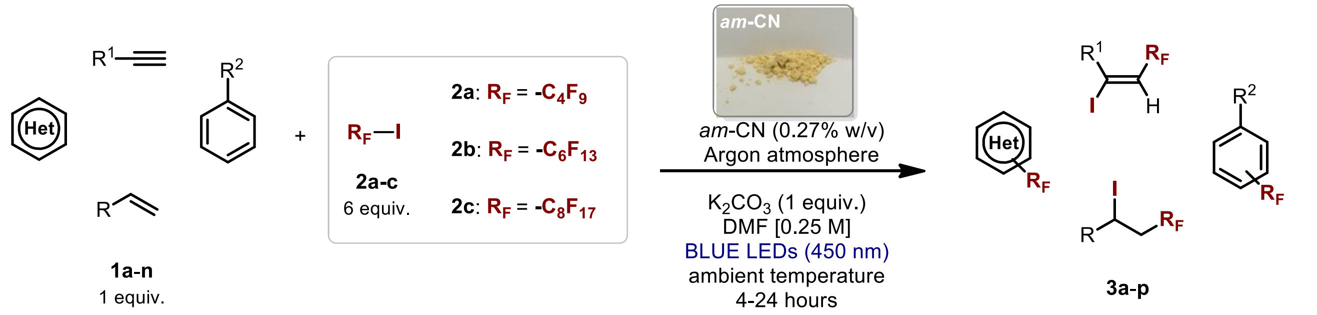

Scheme S1. General scheme for the fluoroalkylation of organic compounds. Photo credit: Francesco Longobardo, University of Trieste (Italy)

A 10 mL Schlenk tube was charged with the appropriate electron-rich organic compound **1** (0.1 mmol, 1 equiv.), perfluoroalkyl iodide **2** (0.6 mmol, 6 equiv.), potassium carbonate (0.1 mmol, 1 equiv.) and *am*-CN (0.27 w/v, 1.4 mg). To this suspension was then added *N*,*N*-dimethylformamide (0.4 mL, $[\mathbf{1}]_0$ = 0.25 M). The reaction mixture was thoroughly degassed via freeze-pump-thaw cycles (x 3) and the schlenk tube was filled with argon and placed in the centre of 8 blue LEDs system at 450 nm (3.5 V and 700 mA controlled by an external power supply). The LEDs were produced and purchased by AddicoreTM (for more details, see: https://www.addicore.com/3W-Royal-Blue-LED-on-Star-Board-Heatsink-p/ad425.htm).
Stirring (400 rpm) was maintained for 24 hours (4 hours for compounds **3g-p**) and then the irradiation was stopped. The reaction crude was diluted with a 5% lithium chloride solution and extracted with ethyl acetate (three times). The organic phase was filtered through sodium sulfate. The solvent was removed under reduced pressure and the residue was purified by column chromatography (eluent: *n*-hexane/ethyl acetate) to give the corresponding fluoroalkyl compound **3**.

Calculation of yields

The production yields listed in Table 1 and referred to product 3a were determined by ^{1}H-NMR spectroscopy in $CDCl_3$ using 1,1,2-trichloroethylene (0.10 mmol, 9 μL) as the internal standard (I.S.). The following formula has been used:

$$Production\ Yield(\%) = \frac{\text{Integration of the NMR signal of 3a } [\delta\ 6.2\ \text{ppm } (s, 2\text{H})] \text{ x } 50}{\text{Integration of the NMR signal of the I. S. } [\delta\ 6.5\ \text{ppm } (s, 1\text{H})]}$$

The isolated yields of products 3a-3p listed in Scheme 1 have been calculated using the following formula:

$$Isolated\ Yield(\%) = \frac{\text{Actual Yield x 100}}{\text{Theretical Yield}}$$

For the calculation of the Apparent Quantum yield (AQY) the following equation was used:

$$AQY = \frac{number\ of\ moles\ of\ product\ formed}{number\ of\ moles\ of\ incident\ photons}$$

The moles of product and emitted photons were calculated after 5 hours of a photocatalytic experiment under typical conditions.

Characterization Data of the reaction products

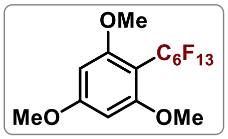

1,3,5-trimethoxy-2-(perfluorobutyl)benzene (3a). Prepared according to the general procedure using 1,3,5-trimethoxybenzene **1a** (0.1 mmol, 17 mg) and nonafluoro-1-iodobutane **2a** (0.6 mmol, 103 μL). The product **3a** was obtained as white solid (37 mg, 96% yield). The characterization of the compound matches with the data reported in the literature.(*55*) ^{1}H-NMR (400 MHz, $CDCl_3$) δ 6.15 (s, 2H), 3.84 (s, 3H), 3.80 (s, 6H); ^{19}F-NMR (376 MHz, $CDCl_3$) δ -80.97 (m, 3F), -102.86 (m, 2F), -123.00 (m, 2F), -126.44 (m, 2F); HRMS calculated for $C_{13}H_{11}F_9O_3$ (M-Na): 409.0462, found: 409.0463.

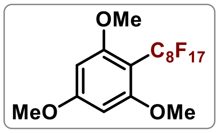

1,3,5-trimethoxy-2-(perfluorohexyl)benzene (3b). Prepared according to the general procedure using 1,3,5-trimethoxybenzene **1a** (0.1 mmol, 17 mg) and perfluorohexyl iodide **2b** (0.6 mmol, 130 μL). The product **3a** was obtained as white solid (45 mg, 92% yield). The characterization of the compound matches with the data reported in the literature.(*56*) ^{1}H-NMR (400 MHz, $CDCl_3$) δ 6.13 (s, 2H), 3.85 (s, 3H), 3.81 (s, 6H); ^{19}F-NMR (376 MHz, $CDCl_3$) δ -80.83 (m, 3F), -102.67 (m, 2F), -122.14 (m, 4F), -122.69 (m, 2F), -126.17 (m, 2F); HRMS calculated for $C_{15}H_{11}F_{13}O_3$ (M-Na): 509.0398, found: 509.0390.

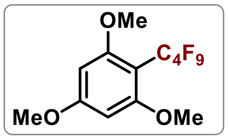

1,3,5-trimethoxy-2-(perfluorooctyl)benzene (3c). Prepared according to the general procedure using 1,3,5-trimethoxybenzene **1a** (0.1 mmol, 17 mg) and heptadecafluoro-1-iodooctane **2c** (0.6 mmol, 158 μL). The product **3c** was obtained as white solid (52 mg, 90% yield). The characterization of the compound matches with the data reported in the literature.(*56*) ^{1}H-NMR (400 MHz, $CDCl_3$) δ 6.15 (s, 2H), 3.84 (s, 3H), 3.80 (s, 6H); ^{19}F-NMR (376 MHz, $CDCl_3$) δ -80.84 (t, *J* = 10.0 Hz, 3F), -102.66 (m, 2F), -121.75 (m, 2F), -122.05 (m, 6F), -122.79 (m, 2F), -126.15 (m, 2F); HRMS calculated for $C_{17}H_{11}F_{17}O_3$ (M-Na): 609.0334, found: 609.0335.

2,6-diisopropyl-4-(perfluorohexyl)aniline (3d). Prepared according to the general procedure using 2,6-diisopropylaniline **1b** (0.1 mmol, 19 µL) and perfluorohexyl iodide **2b** (0.6 mmol, 130 µL). The product **3d** was obtained as white solid (42 mg, 86% yield). The characterization of the compound matches with the data reported in the literature.(*57*) ^{1}H NMR (400 MHz, $CDCl_3$) δ 7.19 (s, 2H), 4.05 (s, 2H), 2.91 (hept, *J* = 6.8 Hz, 2H), 1.29 (d, *J* = 6.8 Hz, 12H) ; ^{19}F NMR (376 MHz, $CDCl_3$) δ -80.85 (m, 3F), -109.41 (m, 2F), -121.57 (m, 2F), -121.89 (m, 2F), -122.85 (m, 2F), -126.17 (m, 2F) ; HRMS calculated for $C_{18}H_{18}F_{13}N$ (M-H): 496.1309, found: 496.1306.

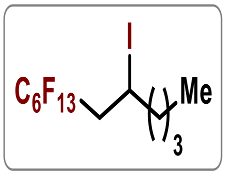

Methyl 5-acetyl-2-hydroxy-3-(perfluorohexyl)benzoate (3e). Prepared according to the general procedure using methyl 5-acetylsalicylate **1c** (0.1 mmol, 19 mg) and perfluorohexyl iodide **2b** (0.6 mmol, 130 µL). The product **3e** was obtained as white solid (23 mg, 45% yield). The characterization of the compound matches with the data reported in the literature.(*58*) ^{1}H NMR (400 MHz, $CDCl_3$) δ 8.68 (s, 1H), 8.31 (s, 1H), 4.04 (s, 3H), 2.61 (s, 3H); ^{19}F NMR (376 MHz, $CDCl_3$) δ -80.95 (t, *J* = 10.0 Hz, 3F), -108.97 (t, *J* = 14.3 Hz, 2F), -121.18 (m, 2F), -121.90 (m, 2F), -122.85 (m, 2F), -126.22 (m, 2F); HRMS calculated for $C_{16}H_8F_{13}O_4$ (M-H): 511.0220, found: 511.0218.

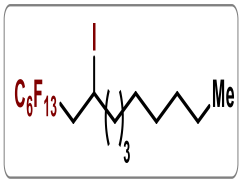

1,3,7-trimethyl-8-(perfluorohexyl)-3,7-dihydro-1*H*-purine-2,6-dione (3f). Prepared according to the general procedure using caffeine **1d** (0.1 mmol, 19 mg) and perfluorohexyl iodide **2b** (0.6 mmol, 130 µL) over 48 hours of irradiation. The product **3f** was obtained as white solid (14 mg, 40% yield). The characterization of the compound matches with the data reported in the literature.(*59*) ^{1}H NMR (400 MHz, $CDCl_3$) δ 4.19 (s, 3H), 3.60 (s, 3H), 3.42 (s, 3H); ^{19}F NMR (376 MHz, $CDCl_3$) δ -80.74 (m, 3F), -108.96 (m, 2F), -121.01 (m, 2F), -121.39 (m, 2F), -122.71 (m, 2F), -126.06 (m, 2F); HRMS calculated for $C_{14}H_{11}F_{13}N_4O_2$ (M-K): 553.0311, found: 553.0526.

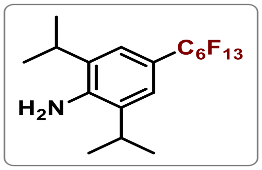

1,1,1,2,2,3,3,4,4,5,5,6,6-tridecafluoro-8-iodododecane (3g). Prepared according to the general procedure using 1-hexene **1e** (0.1 mmol, 13 µL) and perfluorohexyl iodide **2b** (0.6 mmol, 130 µL). The product **3g** was obtained as colorless oil (48 mg, 91% yield). The characterization of the compound matches with the data reported in the literature.(*54*) ^{1}H NMR (400 MHz, $CDCl_3$) δ 4.43 – 4.26 (m, 1H), 3.04 – 2.66 (m, 2H), 1.94 – 1.72 (m, 2H), 1.58 – 1.22 (m, 4H), 0.93 (t, *J* = 7.2 Hz, 3H); ^{19}F NMR (376 MHz, $CDCl_3$) δ -80.86 (m, 3F), -111.92 (m, 1F), -114.69 (m, 1F), -121.84 (m, 2F), -122.91 (m, 2F), -123.69 (m, 2F), -126.19 (m, 2F) ; It was not possible to measure the HRMS (ESI-MS) of compound **3g** due to its poor tendency to ionize.

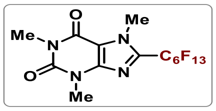

1,1,1,2,2,3,3,4,4,5,5,6,6-tridecafluoro-8-iodohexadecane (3h). Prepared according to the general procedure using 1-decen **1f** (0.1 mmol, 19 µL) and perfluorohexyl iodide **2b** (0.6 mmol, 130 µL). The product **3h** was obtained as colorless oil (54 mg, 83% yield). The characterization of the compound matches with the data reported in the literature.(*54*) ^{1}H-NMR (400 MHz, $CDCl_3$) δ 4.40 – 4.27 (m, 1H), 3.06 – 2.63 (m, 2H), 1.90 – 1.69 (m, 2H), 1.43 – 1.18

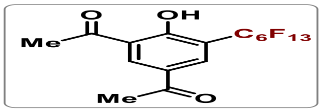

(m, 12H), 0.89 (t, *J* = 6.9 Hz, 3H); ^{19}F NMR (376 MHz, $CDCl_3$) δ -80.85 (m, 3F), -111.80 (m, 1F), -114.70 (m, 1F), -121.82 (m, 2F), -122.90 (m, 2F), -123.71 (m, 2F), -126.18 (m, 2F) ; It was not possible to measure the HRMS (ESI-MS) of compound **3h** due to its poor tendency to ionize.

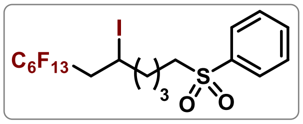

((7,7,8,8,9,9,10,10,11,11,12,12,12-tridecafluoro-5-iodododecyl)sulfonyl)benzene (3i). Prepared according to the general procedure using (hex-5-en-1-ylsulfonyl)benzene **1g** (0.1 mmol, 22 mg) and perfluorohexyl iodide **2b** (0.6 mmol, 130 µL). The product **3i** was obtained as colorless oil (57 mg, 84% yield). The characterization of the compound matches with the data reported in the literature.(*54*) ^{1}H-NMR (400 MHz, $CDCl_3$) δ 7.95–7.88 (m, 2H), 7.71–7.64 (m, 1H), 7.58 (t, *J* = 7.6 Hz, 2H), 4.26 (ddd, *J* = 13.3, 8.5, 5.2 Hz, 1H), 3.15–3.08 (m, 2H), 2.99–2.62 (m, 2H), 1.87–1.70 (m, 4H), 1.71–1.60 (m, 1H), 1.56–1.45 (m, 1H); ^{19}F-NMR (376 MHz, $CDCl_3$) δ -80.80 (m, 3F), -111.52 (m, 1F), -114.69 (m, 1F), -121.80 (m, 2F), -122.85 (m, 2F), -123.61 (m, 2F), -126.09 (m, 2F); HRMS calculated for $C_{18}H_{16}F_{13}IO_2S$ (M-Na): 692.9606, found: 692.9600.

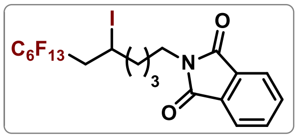

2-(7,7,8,8,9,9,10,10,11,11,12,12,12-tridecafluoro-5-iodododecyl)isoindoline-1,3-dione (3j). Prepared according to the general procedure using 2-(hex-5-en-1-yl)isoindoline-1,3-dione **1h** (0.1 mmol, 23 mg) and perfluorohexyl iodide **2b** (0.6 mmol, 130 µL). The product **3j** was obtained as colorless oil (60 mg, 88% yield). The characterization of the compound matches with the data reported in the literature.(*54*) ^{1}H-NMR (400 MHz, $CDCl_3$) δ 7.84 (dd, *J* = 5.5, 3.0 Hz, 2H), 7.71 (dd, *J* = 5.4, 3.1 Hz, 2H), 4.30 (ddd, *J* = 16.8, 8.3, 5.3 Hz, 1H), 3.71 (t, *J* = 7.2 Hz, 2H), 3.00–2.68 (m, 2H), 1.95–1.39 (m, 6H); ^{19}F-NMR (376 MHz, $CDCl_3$) δ -80.87 (tt, *J* = 10.0, 2.2 Hz, 3F), -111.77 (m, 1F), -114.63 (m, 1F), -121.83 (m, 2F), -122.93 (m, 2F), -123.69 (m, 2F), -126.20 (m, 2F); HRMS calculated for $C_{20}H_{15}F_{13}INO_2$ (M-Na): 697.9837, found: 697.9832.

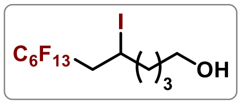

7,7,8,8,9,9,10,10,11,11,12,12,12-tridecafluoro-5-iodododecan-1-ol (3k). Prepared according to the general procedure using 5-hexen-1-ol **1i** (0.1 mmol, 12 µL) and perfluorohexyl iodide **2b** (0.6 mmol, 130 µL). The product **3k** was obtained as colorless oil (52 mg, 94% yield). The characterization of the compound matches with the data reported in the literature.(*54*) ^{1}H-NMR (400 MHz, $CDCl_3$) δ 4.34 (ddd, *J* = 13.4, 8.4, 5.3 Hz, 1H), 3.68 (t, *J* = 6.1 Hz, 2H), 3.03 – 2.68 (m, 2H), 1.93 – 1.75 (m, 2H), 1.71 – 1.44 (m, 4H); 19F-NMR (376 MHz, CDCl3) δ -80.83 (m, 3F), -111.76 (m, 1F), -114.67 (m, 1F), -121.80 (m, 2F), -122.86 (m, 2F), -123.65 (m, 2F), -125.57 – -126.96 (m, 2F); ; HRMS calculated for $C_{12}H_{12}F_{13}IO$ (M-Na): 568.9623, found: 568.9612.

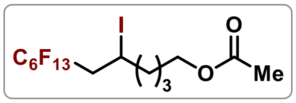

7,7,8,8,9,9,10,10,11,11,12,12,12-tridecafluoro-5-iodododecyl acetate (3l). Prepared according to the general procedure using hex-5-en-1-yl acetate **1j** (0.1 mmol, 14 mg) and perfluorohexyl iodide **2b** (0.6 mmol, 130 µL). The product **3l** was obtained as colorless oil (53 mg, 91% yield). The characterization of the compound matches with the data reported in the literature.(*54*) ^{1}H-NMR (400 MHz, $CDCl_3$) δ 4.32 (ddd, *J* = 13.4, 8.5, 5.1 Hz, 1H), 4.08 (t, *J* = 6.3 Hz, 2H), 3.02–2.68 (m, 2H), 2.05 (s, 3H), 1.91–1.43 (m, 6H); ^{19}F-NMR (376 MHz, $CDCl_3$) δ -80.85 (m, 3F), -111.63 (m, 1F), -114.68 (m,

1F), -121.83 (m, 2F), -122.89 (m, 2F), -123.66 (m, 2F), -126.17 (m, 2F); HRMS calculated for $C_{14}H_{14}F_{13}IO_2$ (M-Na): 610.9728, found: 610.9723.

12-bromo-1,1,1,2,2,3,3,4,4,5,5,6,6-tridecafluoro-8-iodododecane (3m). Prepared according to the general procedure using 6-bromo-1-hexene **1k** (0.1 mmol, 14 µL) and perfluorohexyl iodide **2b** (0.6 mmol, 130 µL). The product **3m** was obtained as colorless oil (58 mg, 95% yield). The characterization of the compound matches with the data reported in the literature.(*54*) ^{1}H-NMR (400 MHz, $CDCl_3$) δ 4.40–4.25 (m, 1H), 3.43 (t, *J* = 6.7 Hz, 1H), 3.27–3.15 (m, 1H), 3.05–2.67 (m, 2H), 2.05–1.59 (m, 6H); ^{19}F-NMR (376 MHz, $CDCl_3$) δ -80.85 (m, 3F), -111.62 (m, 1F), -114.65 (m, 1F), -121.81 (s, 2F), -122.89 (m, 2F), -123.65 (m, 2F), -126.18 (m, 2F); It was not possible to measure the HRMS (ESI-MS) of compound **3m** due to its poor tendency to ionize.

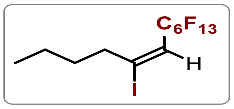

12-chloro-1,1,1,2,2,3,3,4,4,5,5,6,6-tridecafluoro-8-iodododecane (3n). Prepared according to the general procedure using 6-chloro-1-hexene **1l** (0.1 mmol, 13 µL) and perfluorohexyl iodide **2b** (0.6 mmol, 130 µL). The product **3n** was obtained as colorless oil (53 mg, 93% yield). The characterization of the compound matches with the data reported in the literature.(*54*) ^{1}H-NMR (400 MHz, $CDCl_3$) δ 4.33 (ddd, *J* = 13.5, 8.4, 5.2 Hz, 1H), 3.56 (t, *J* = 6.5 Hz, 2H), 3.05–2.65 (m, 2H), 1.92–1.52 (m, 6H); ^{19}F-NMR (376 MHz, $CDCl_3$) δ -80.85 (m, 3F), -111.68 (m, 1F), -114.67 (m, 1F), -121.84 (m, 2F), -122.92 (m, 2F), -123.65 (m, 2F), -126.18 (m, 2F); It was not possible to measure the HRMS (ESI-MS) of compound **3n** due to its poor tendency to ionize.

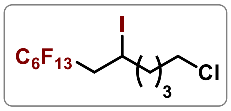

1,1,1,2,2,3,3,4,4,5,5,6,6-tridecafluoro-8-iodo-12-(octyloxy)dodecane (3o). Prepared according to the general procedure using 1-(hex-5-en-1-yloxy)octane **1m** (0.1 mmol, 21 mg) and perfluorohexyl iodide **2b** (0.6 mmol, 130 µL). The product **3o** was obtained as colorless oil (64 mg, 97% yield). The characterization of the compound matches with the data reported in the literature.(*54*) ^{1}H-NMR (400 MHz, $CDCl_3$) δ 4.45–4.24 (m, 1H), 3.51–3.34 (m, 4H), 3.04–2.68 (m, 2H), 1.95–1.74 (m, 2H), 1.71–1.44 (m, 6H), 1.40–1.20 (m, 10H), 0.88 (t, *J* = 6.9 Hz, 3H); ^{19}F-NMR (376 MHz, $CDCl_3$) δ -80.82 (tt, *J* = 9.9, 2.2 Hz, 3F), -111.82 (m, 1F), -114.61 (m, 1F), -121.81 (m, 2F), -122.88 (m, 2F), -123.66 (m, 2F), -126.17 (m, 2F); HRMS calculated for $C_{20}H_{28}F_{13}IO$ (M-Na): 681.0875, found: 681.0870.

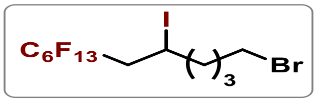

(*E*)-7,7,8,8,9,9,10,10,11,11,12,12,12-tridecafluoro-5-iodododec-5-ene (3p). Prepared according to the general procedure using 1-hexyne **1n** (0.1 mmol, 12 µL) and perfluorohexyl iodide **2b** (0.6 mmol, 130 µL). The product **3p** was obtained as colorless oil (34 mg, 64% yield, 4:1 *E*/*Z*). The characterization of the compound matches with the data reported in the literature.(*54*) (*E*)-isomer: ^{1}H-NMR (400 MHz, $CDCl_3$) δ 6.35 (*t*, *J* = 14.4 Hz, 1H), 2.66 (t, *J* = 7.6 Hz, 2H), 1.59 (m, 2H), 1.38 (m, 2H), 0.97 (t, *J* = 7.3 Hz, 3H). The (*Z*)-isomer appears at 6.16 ppm; . (*E*)-isomer: ^{19}F-NMR (376 MHz, $CDCl_3$) δ -80.82 (t, *J* = 10.4 Hz, 3F), -105.46 (t, *J* = 13 Hz, 2F), -121.70 (m, 2F), -122.86 (m, 2F), -123.30 (m, 2F), -126.17 (m, 2F). The (*Z*)-isomer appears at – 108.50 ppm; It was not possible to measure the HRMS (ESI-MS) of compound **3p** due to its poor tendency to ionize.

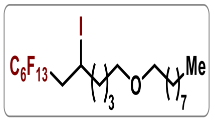

Uv-vis Spectra of starting materials

Photocatalyst, namely *am*-CN, is the only species that can absorb light at 450 nm. We did not observe any ground-state association between **1a** and the radical source **2a**, because their combination does not lead to relevant change of the absorption spectra (Figure S12, black line overlays the red line).

NMR Spectra of the product

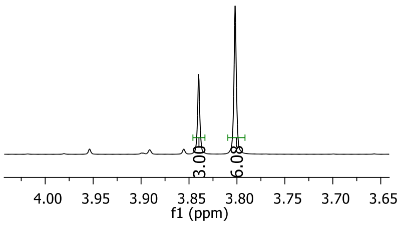

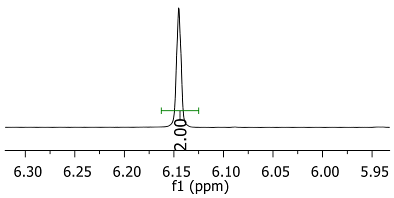

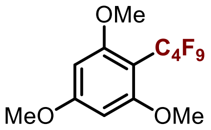

3a

^{1}H NMR (400 MHz, $CDCl_3$)

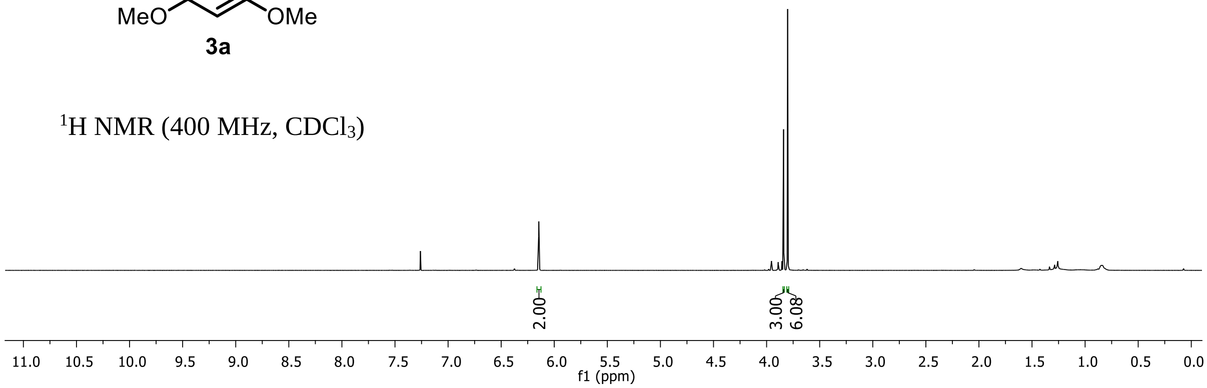

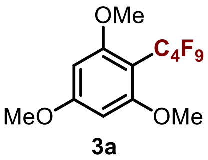

^{19}F NMR (376 MHz, $CDCl_3$)

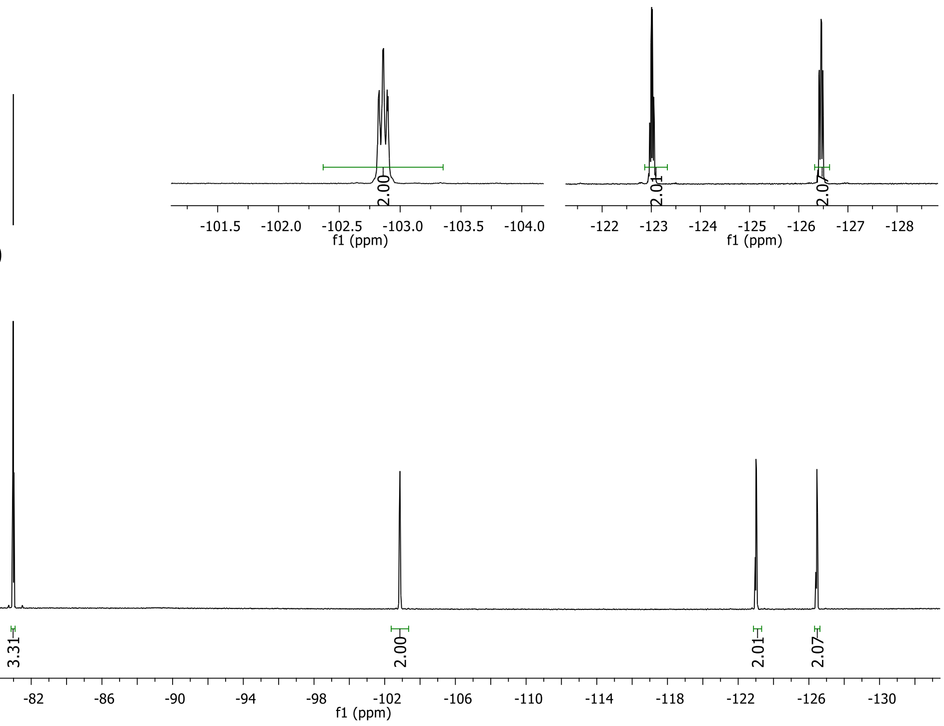

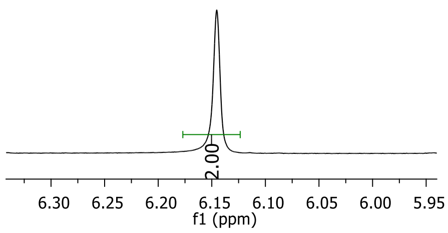

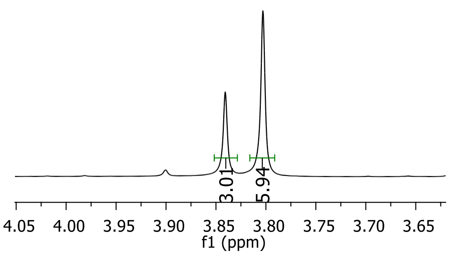

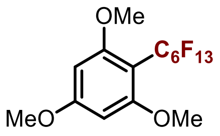

3b

^{1}H NMR (400 MHz, $CDCl_3$)

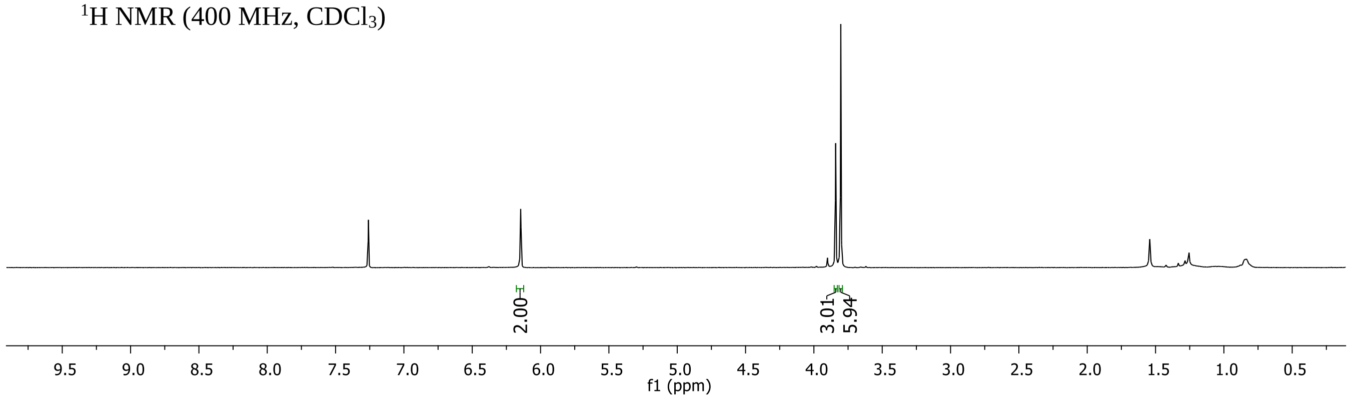

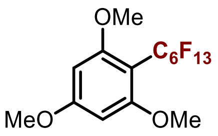

3b

^{19}F NMR (376 MHz, $CDCl_3$)

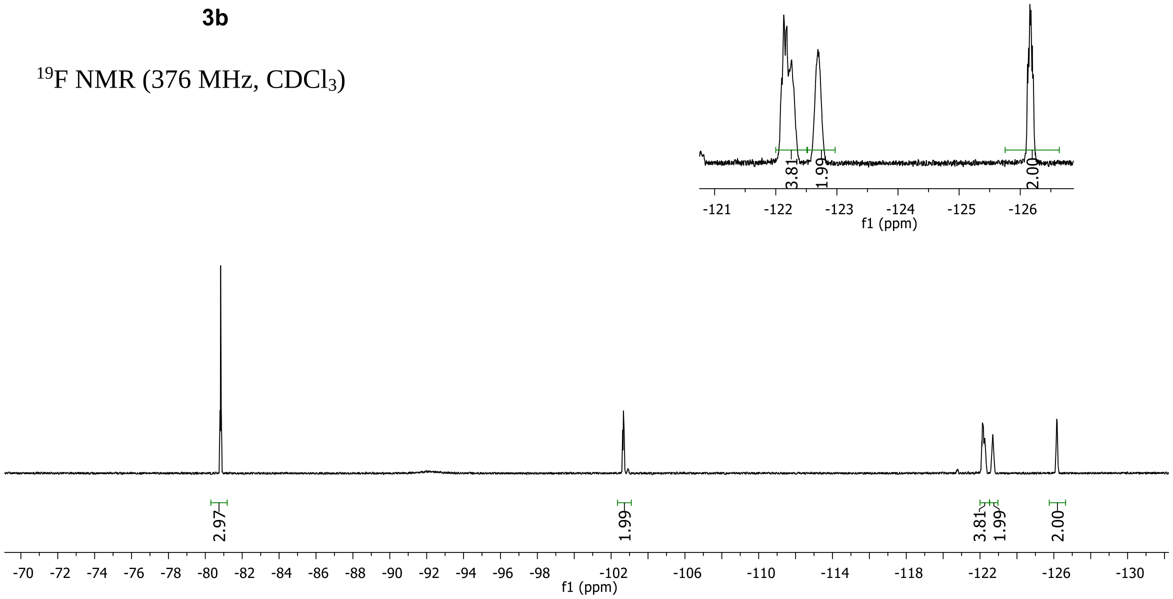

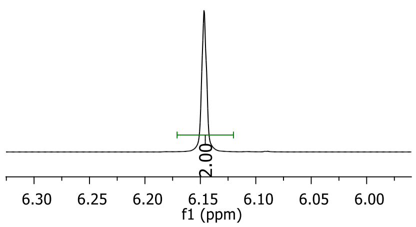

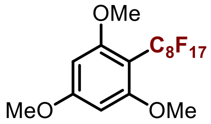

3c

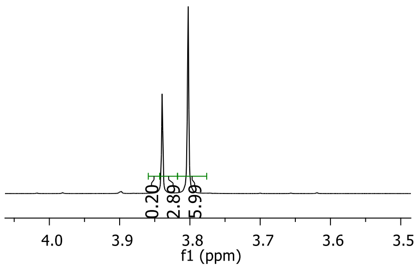

^{1}H NMR (400 MHz, $CDCl_3$)

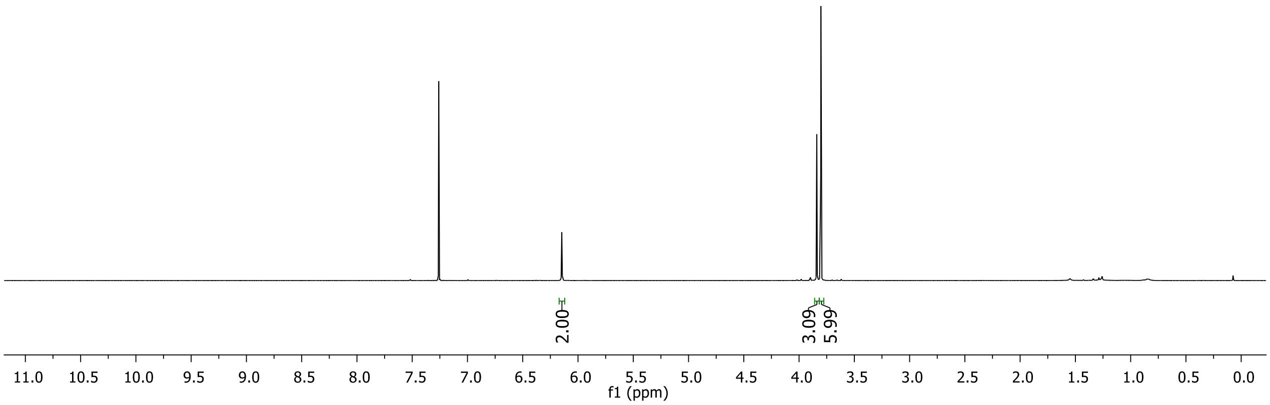

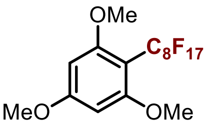

3c

^{19}F NMR (376 MHz, $CDCl_3$)

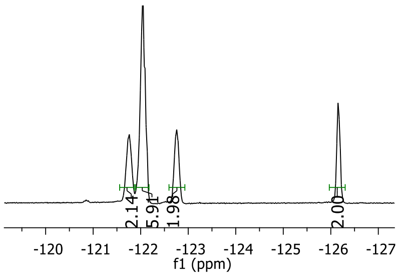

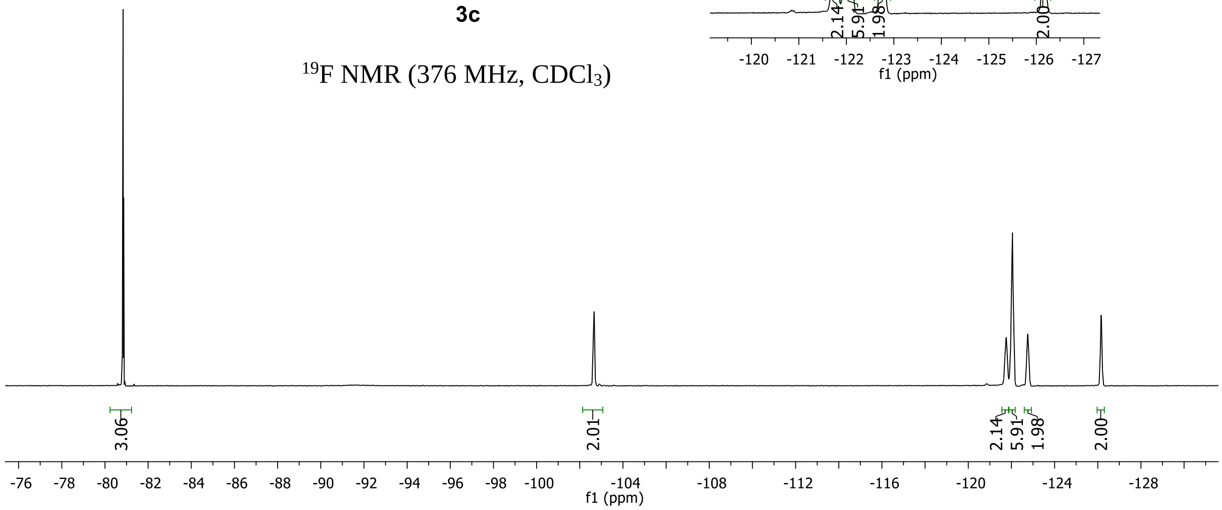

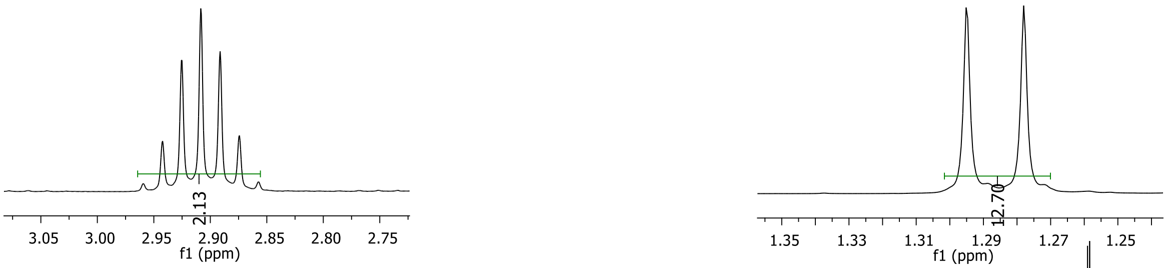

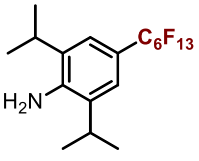

3d

^{1}H NMR (400 MHz, $CDCl_3$)

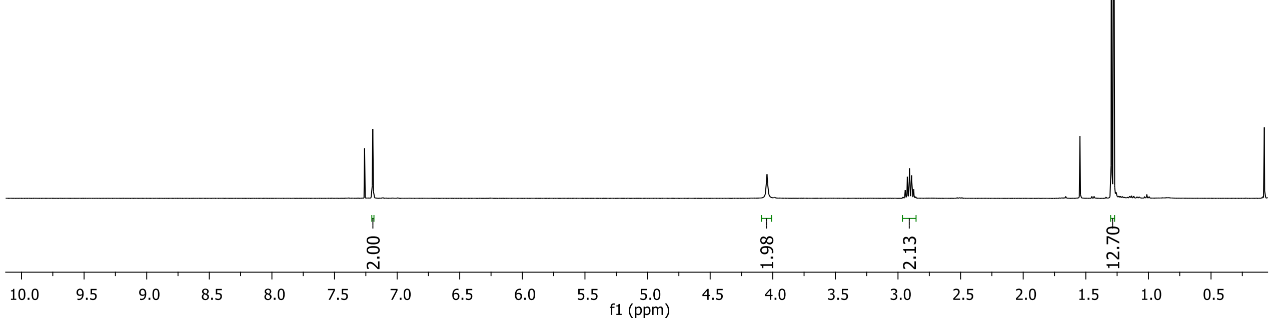

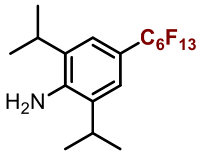

3d

^{19}F NMR (376 MHz, $CDCl_3$)

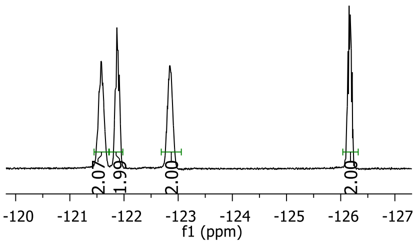

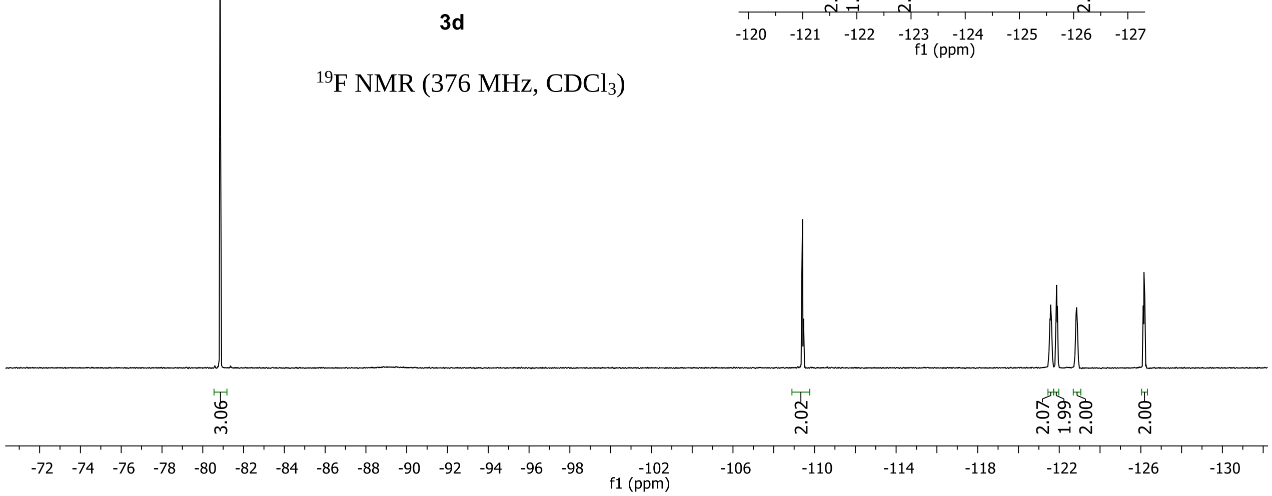

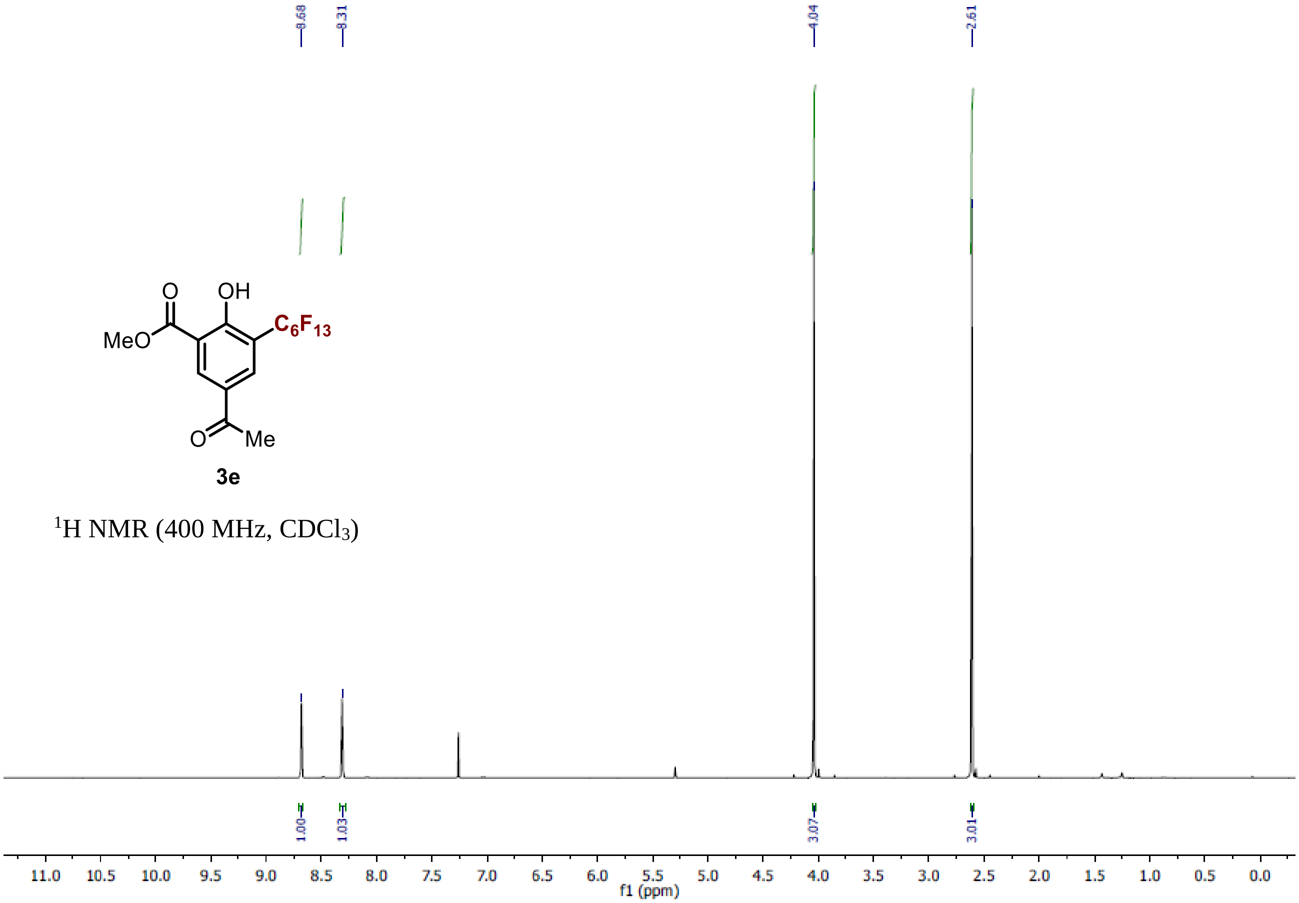
8.68
8.31
4.04
2.61
MeO
O
OH
C_6F_{13}
Me
3e
^{1}H NMR (400 MHz, $CDCl_3$)
1.00
1.03
3.07
3.01
11.0 10.5 10.0 9.5 9.0 8.5 8.0 7.5 7.0 6.5 6.0 5.5 5.0 4.5 4.0 3.5 3.0 2.5 2.0 1.5 1.0 0.5 0.0
f1 (ppm)

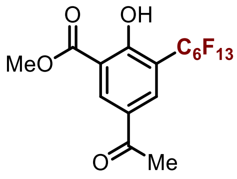

3e

^{19}F NMR
(376 MHz, $CDCl_3$)

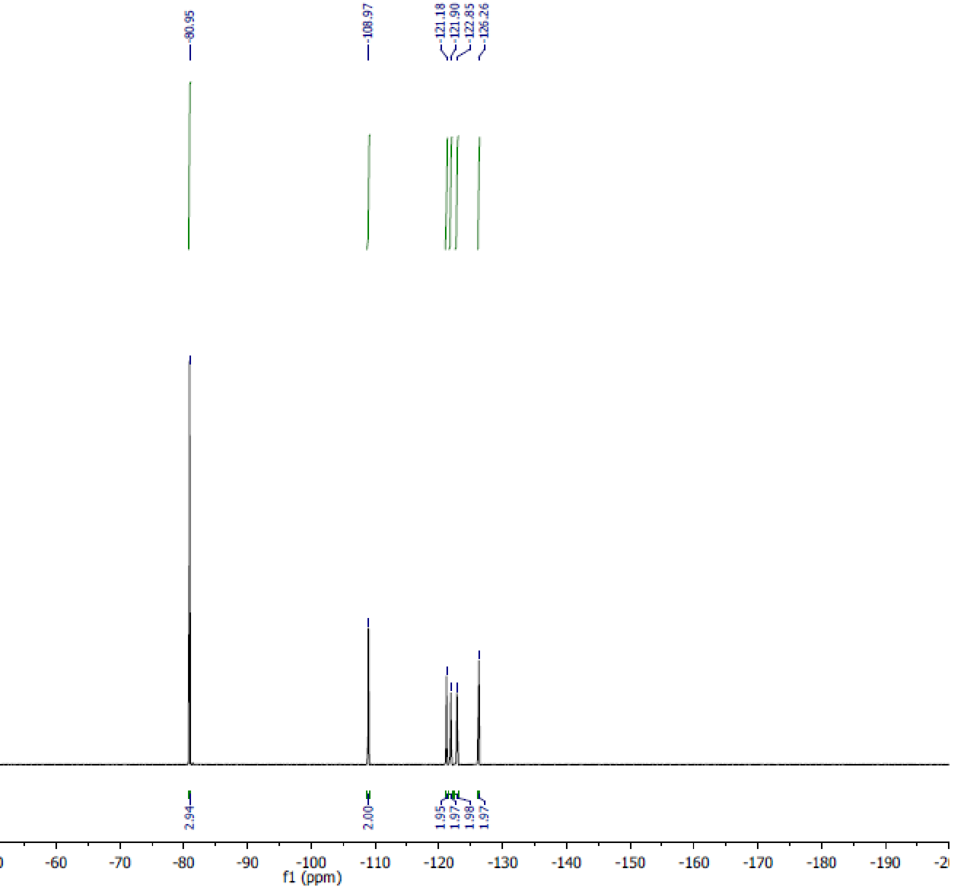

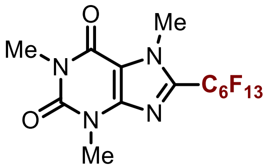

3f

^{1}H NMR (400 MHz, $CDCl_3$)

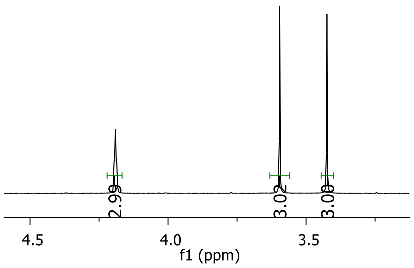

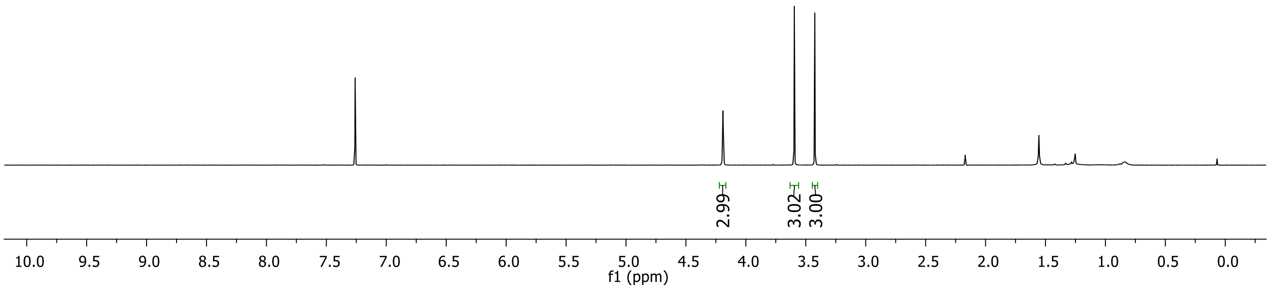

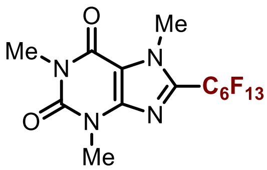

3f

^{19}F NMR
(376 MHz, $CDCl_3$)

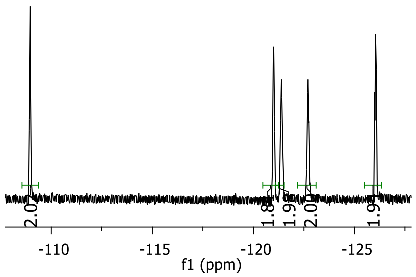

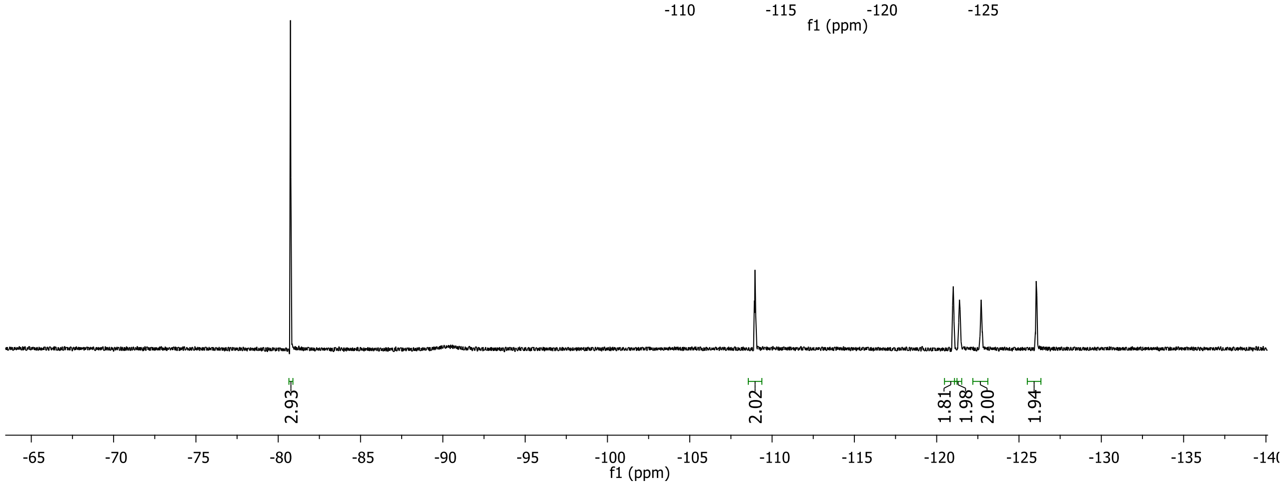

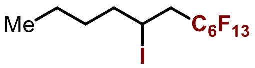

3g

^1H NMR (400 MHz, $CDCl_3$)

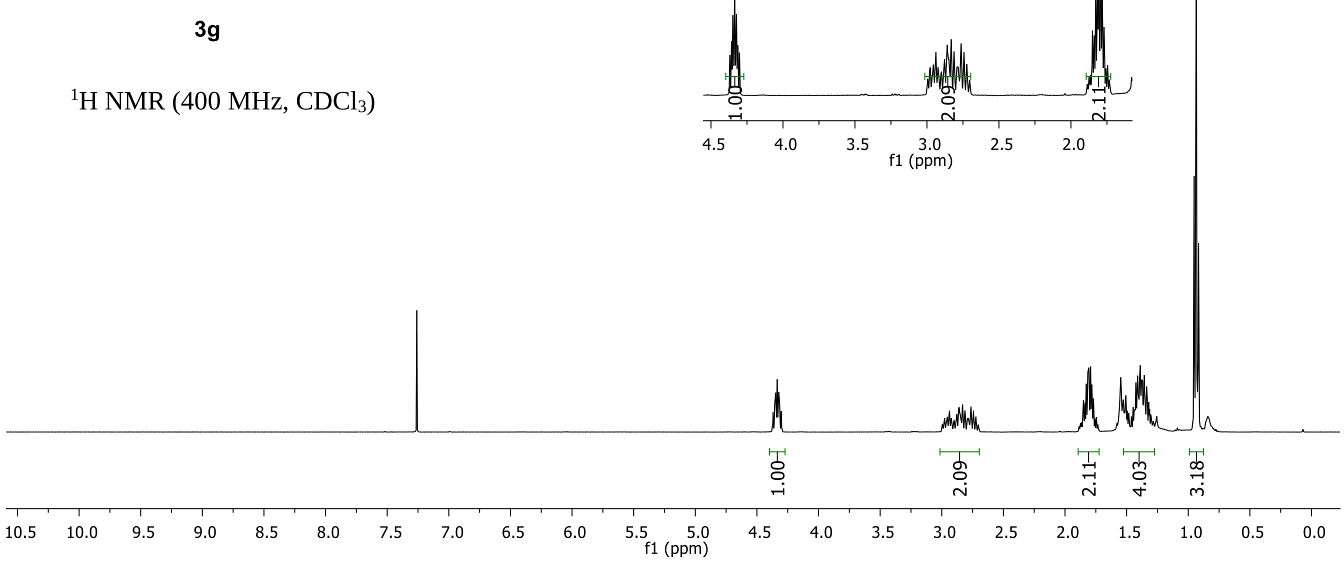

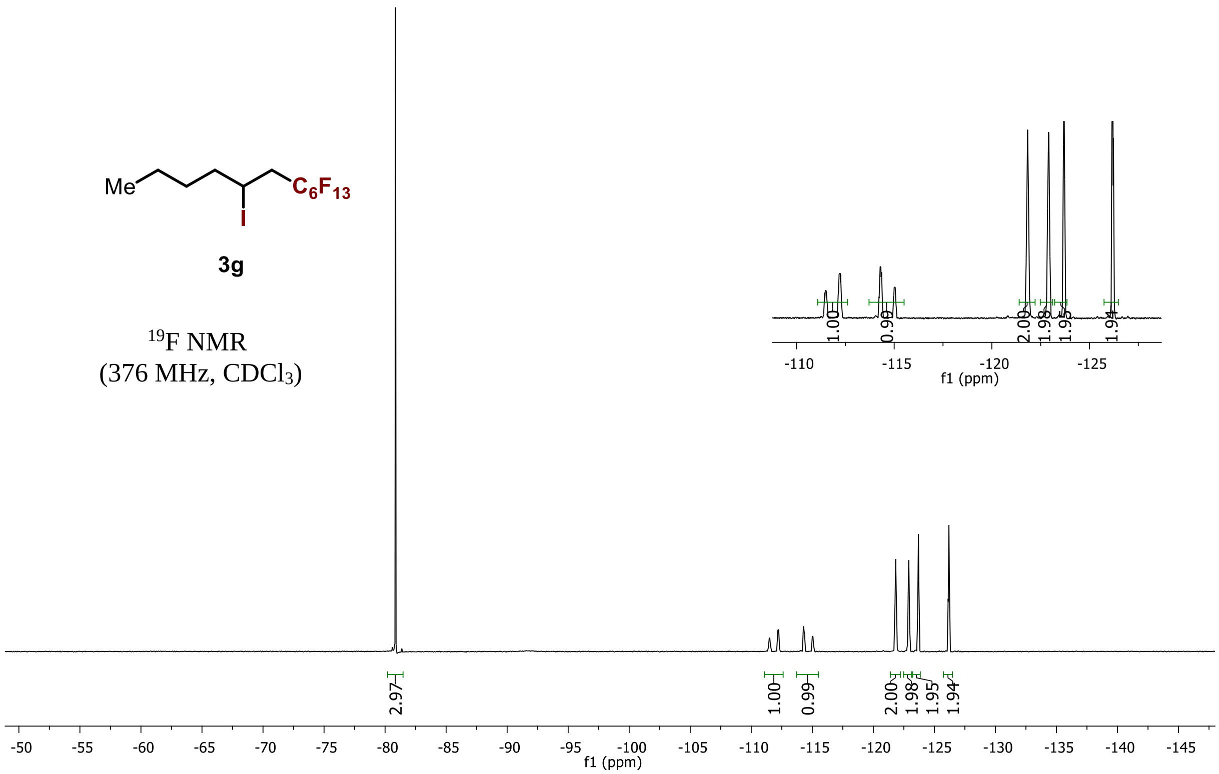

Me
C6F13
I
3g
19F NMR
(376 MHz, CDCl3)
1.00
0.99
2.00
1.98
1.95
1.94
-110
-115
-120
-125
f1 (ppm)
2.97
-50
-55
-60
-65
-70
-75
-80
-85
-90
-95
-100
-105
-110
-115
-120
-125
-130
-135
-140
-145
f1 (ppm)

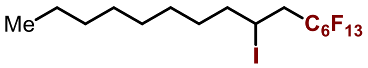

3h

^{1}H NMR (400 MHz, $CDCl_3$)

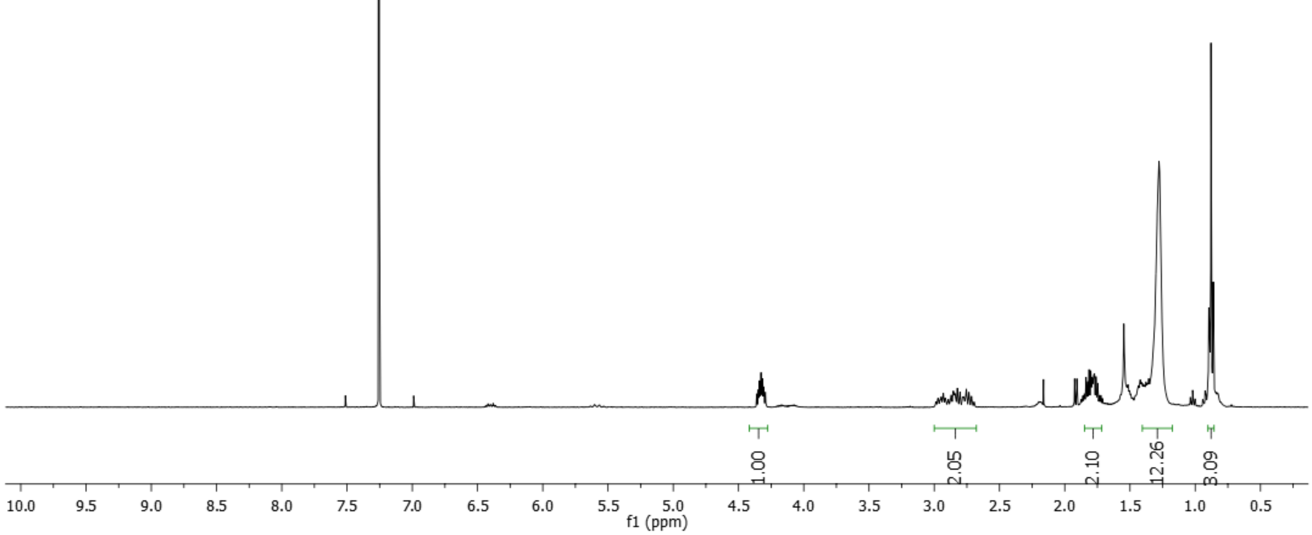

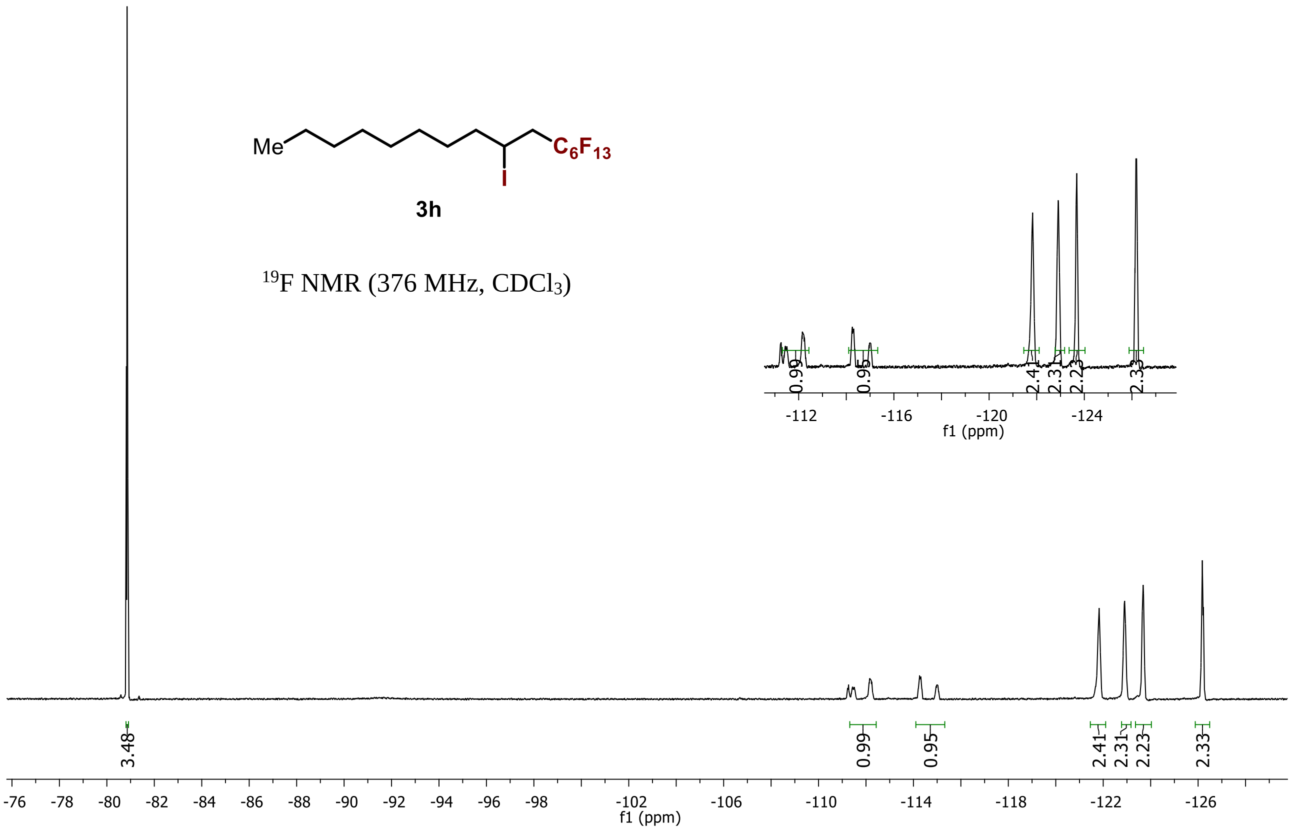

Me
C6F13
I
3h
19F NMR (376 MHz, CDCl3)
0.99
0.95
2.41
2.31
2.23
2.33
-112
-116
-120
-124
f1 (ppm)
3.48
0.99
0.95
2.41
2.31
2.23
2.33
-76
-78
-80
-82
-84
-86
-88
-90
-92
-94
-96
-98
-102
-106
-110
-114
-118
-122
-126
f1 (ppm)

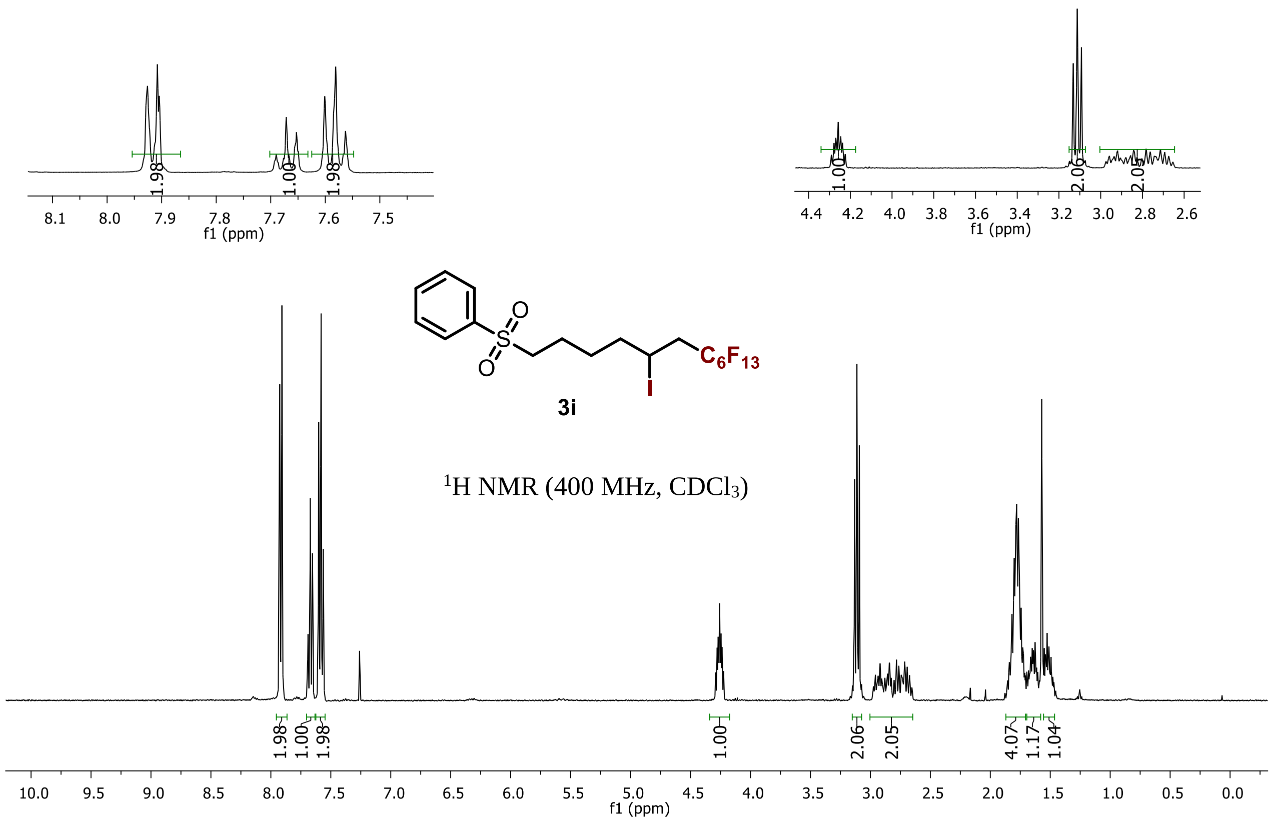

C6F13
I
3i
1H NMR (400 MHz, CDCl3)
f1 (ppm)
1.98
1.00
1.98
2.06
2.05
4.07
1.17
1.04

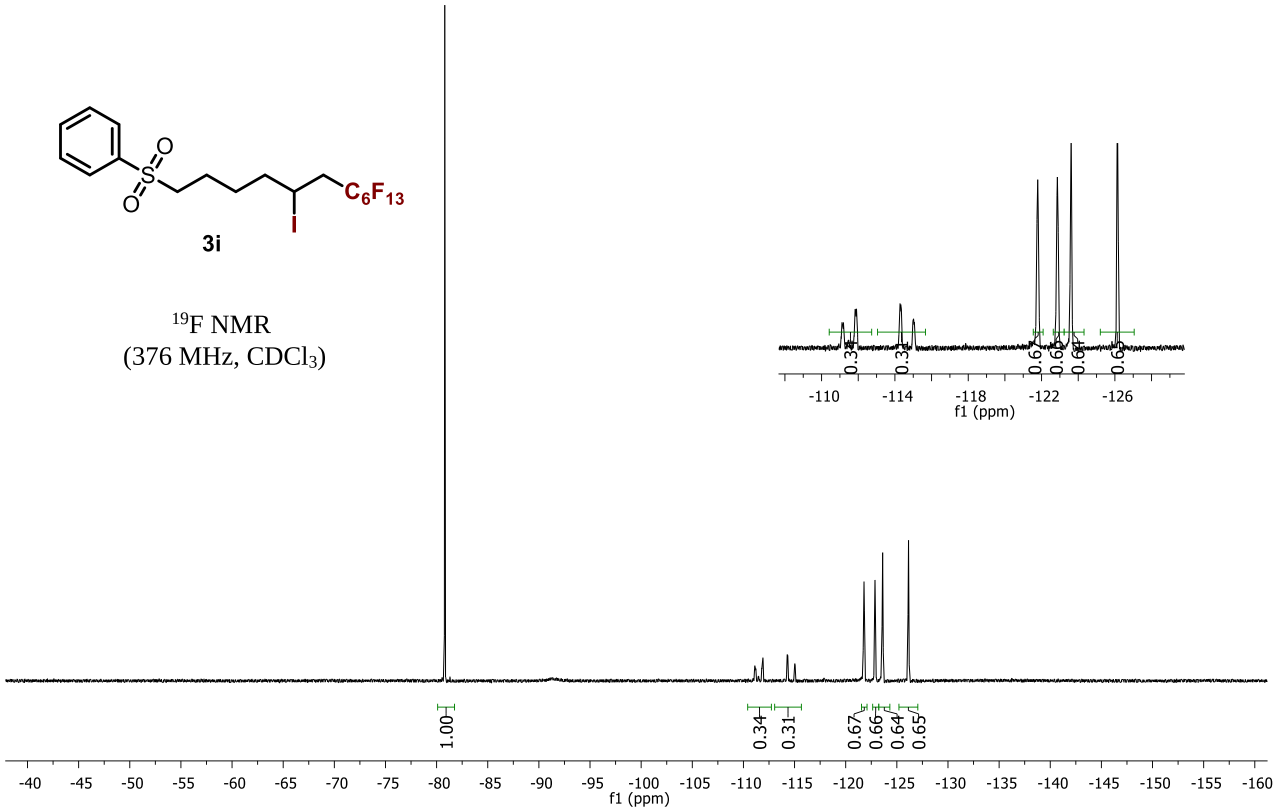
3i
19F NMR
(376 MHz, CDCl3)
1.00
0.34
0.31
0.67
0.66
0.64
0.65
-40 -45 -50 -55 -60 -65 -70 -75 -80 -85 -90 -95 -100 -105 -110 -115 -120 -125 -130 -135 -140 -145 -150 -155 -160
f1 (ppm)
-110 -114 -118 -122 -126
f1 (ppm)

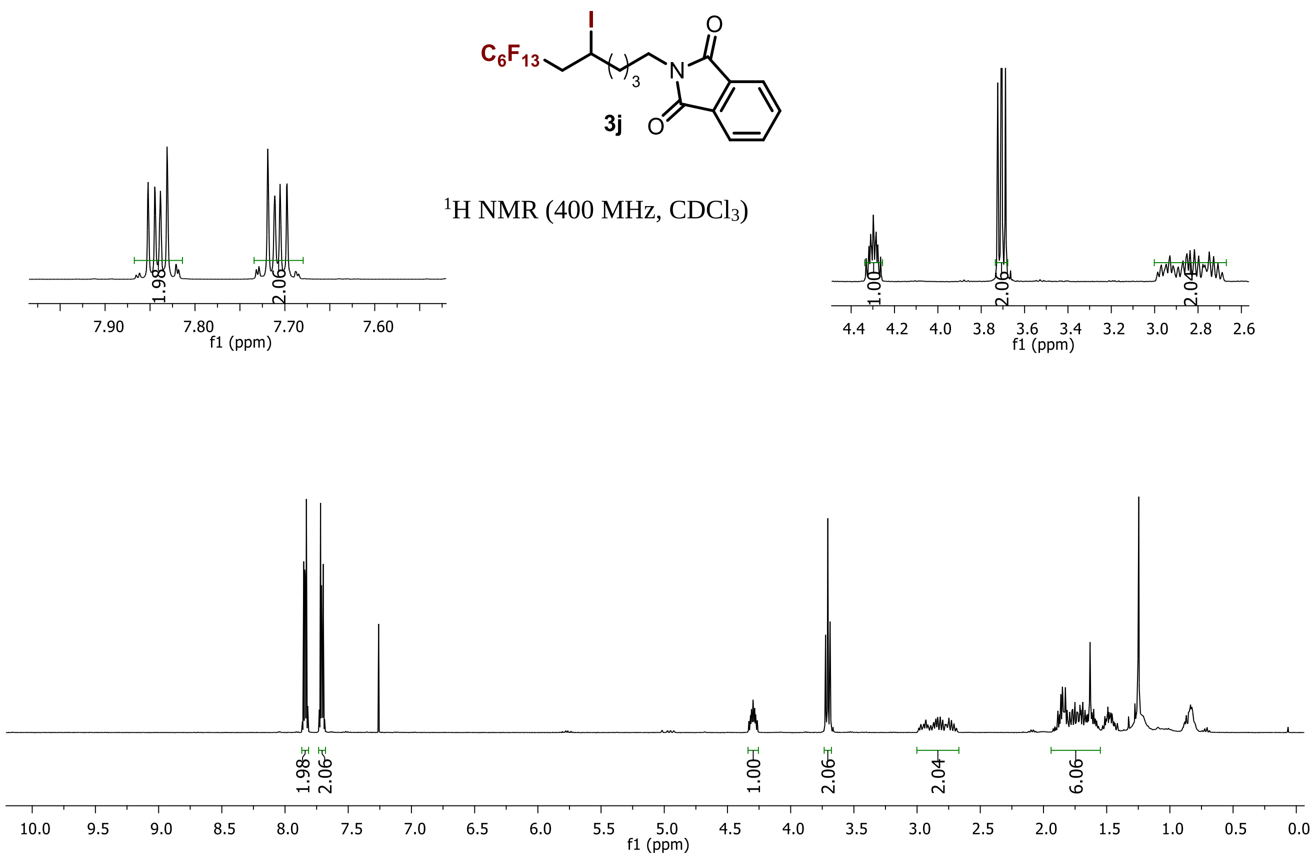

C6F13
I
O
N
O
3
3j
1H NMR (400 MHz, CDCl3)
1.98
2.06
7.90
7.80
7.70
7.60
f1 (ppm)
1.00
2.06
2.04
4.4
4.2
4.0
3.8
3.6
3.4
3.2
3.0
2.8
2.6
f1 (ppm)
1.98
2.06
1.00
2.06
2.04
6.06
10.0
9.5
9.0
8.5
8.0
7.5
7.0
6.5
6.0
5.5
5.0
4.5
4.0
3.5
3.0
2.5
2.0
1.5
1.0
0.5
0.0
f1 (ppm)

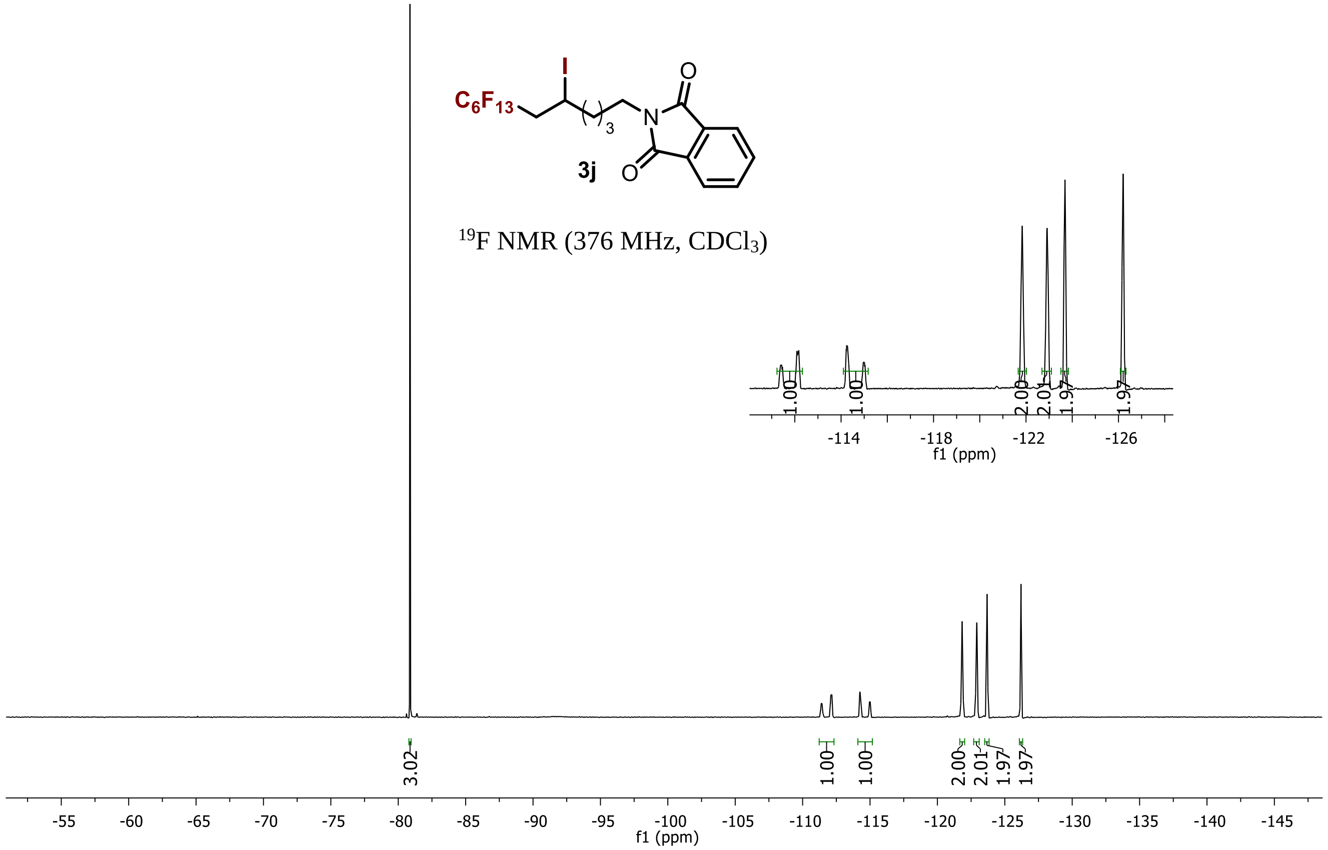

C6F13
I
O
N
3
3j
O
19F NMR (376 MHz, CDCl3)
1.00
1.00
2.00
2.01
1.97
1.97
-114
-118
-122
-126
f1 (ppm)
3.02
1.00
1.00
2.00
2.01
1.97
1.97
-55
-60
-65
-70
-75
-80
-85
-90
-95
-100
-105
-110
-115
-120
-125
-130
-135
-140
-145
f1 (ppm)

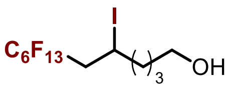

3k

^{1}H NMR (400 MHz, $CDCl_3$)

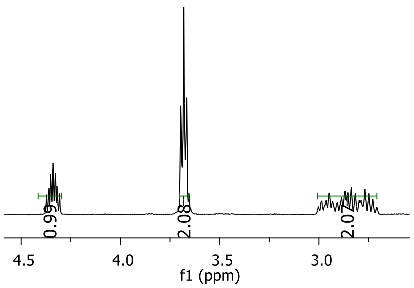

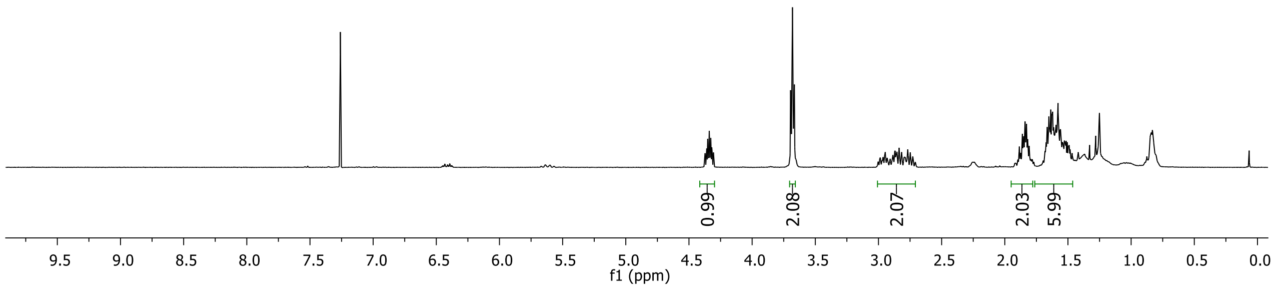

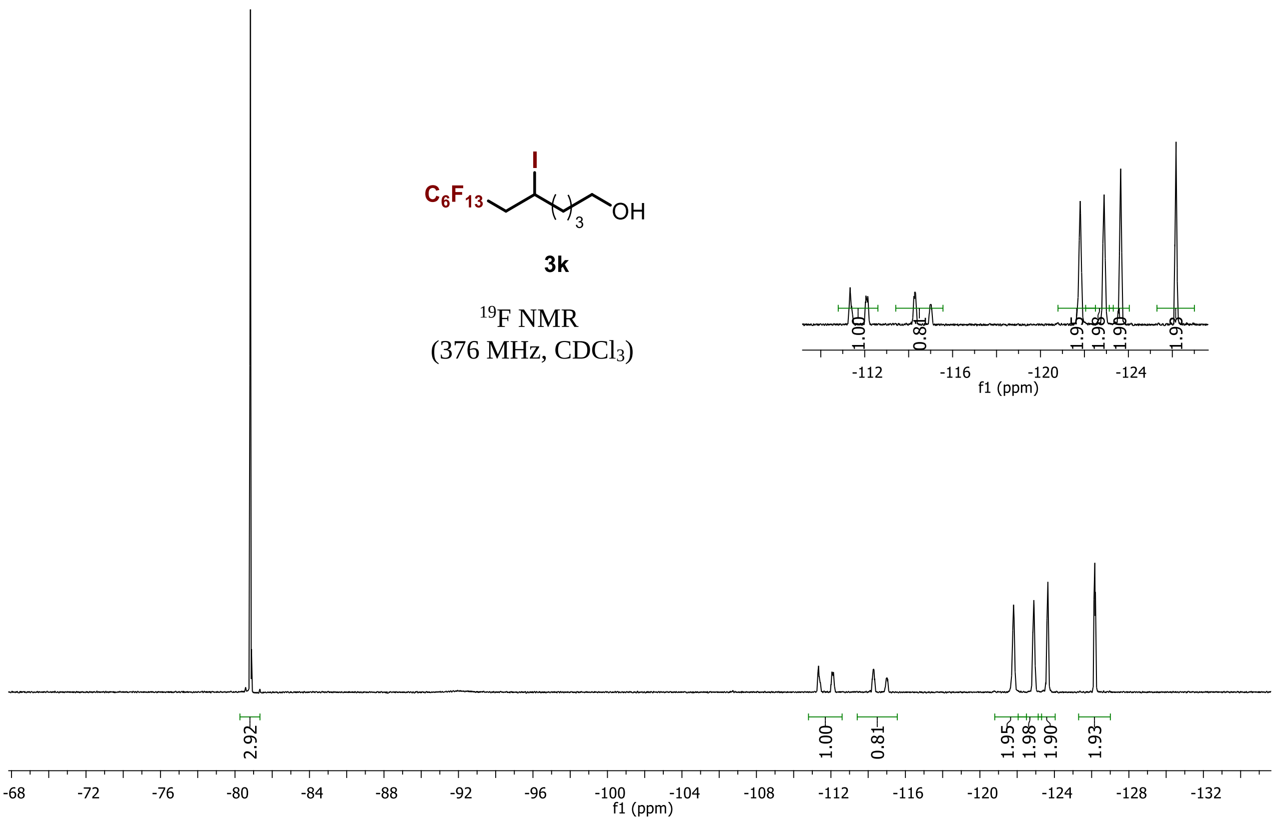

C_6F_{13}
I
3
OH
3k
^{19}F NMR
(376 MHz, $CDCl_3$)
2.92
1.00
0.81
1.95
1.98
1.90
1.93
-68
-72
-76
-80
-84
-88
-92
-96
-100
-104
-108
-112
-116
-120
-124
-128
-132
f1 (ppm)

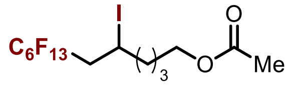

3l

^1H NMR (400 MHz, $CDCl_3$)

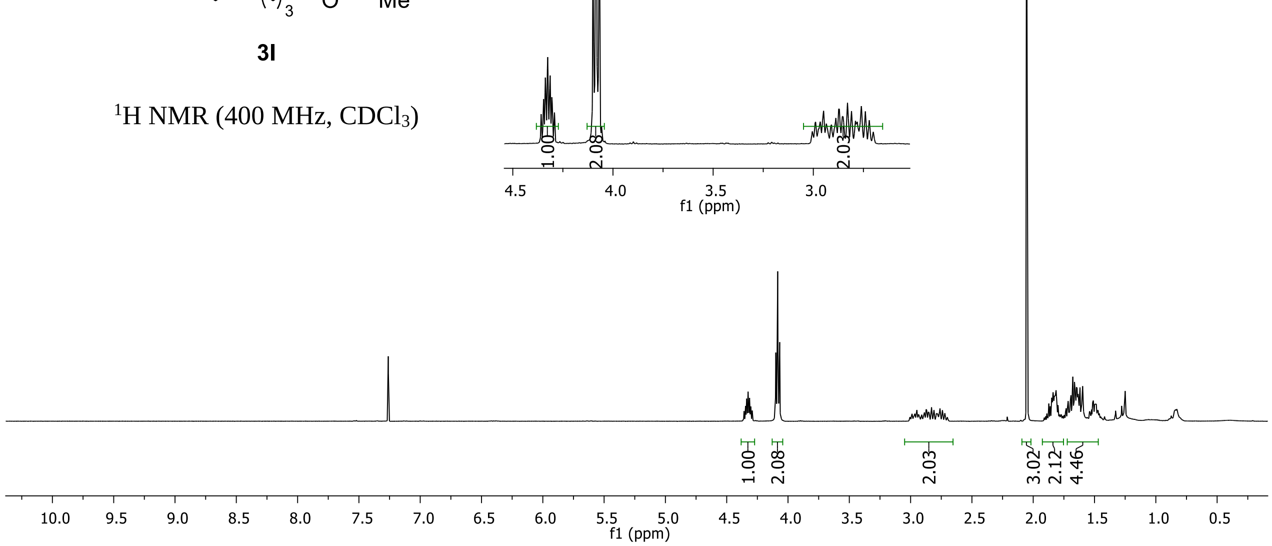

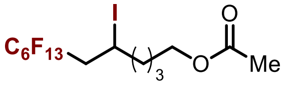

3l

^{19}F NMR (376 MHz, $CDCl_3$)

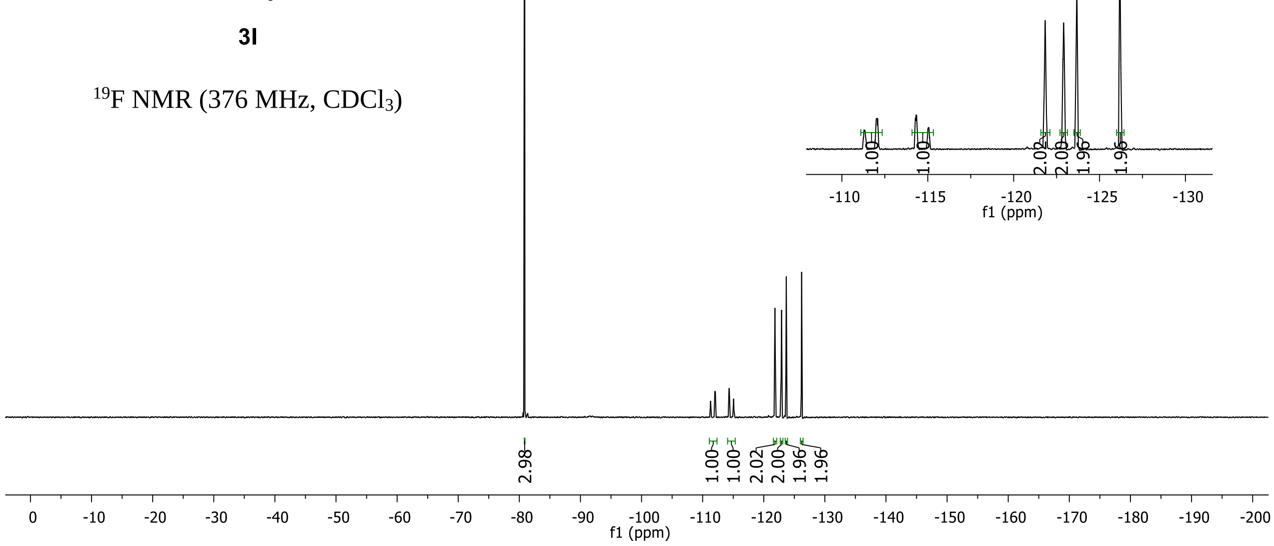

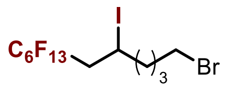

3m

^{1}H NMR (400 MHz, $CDCl_3$)

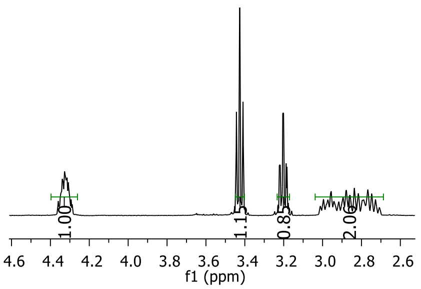

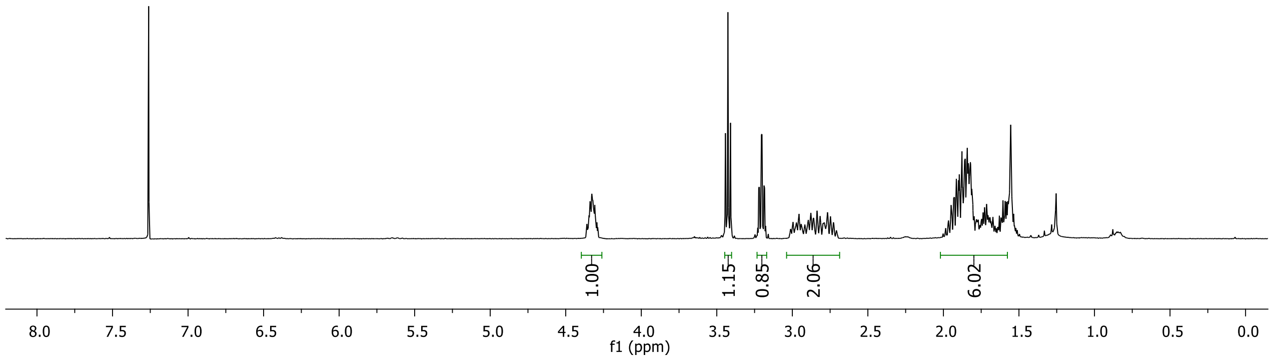

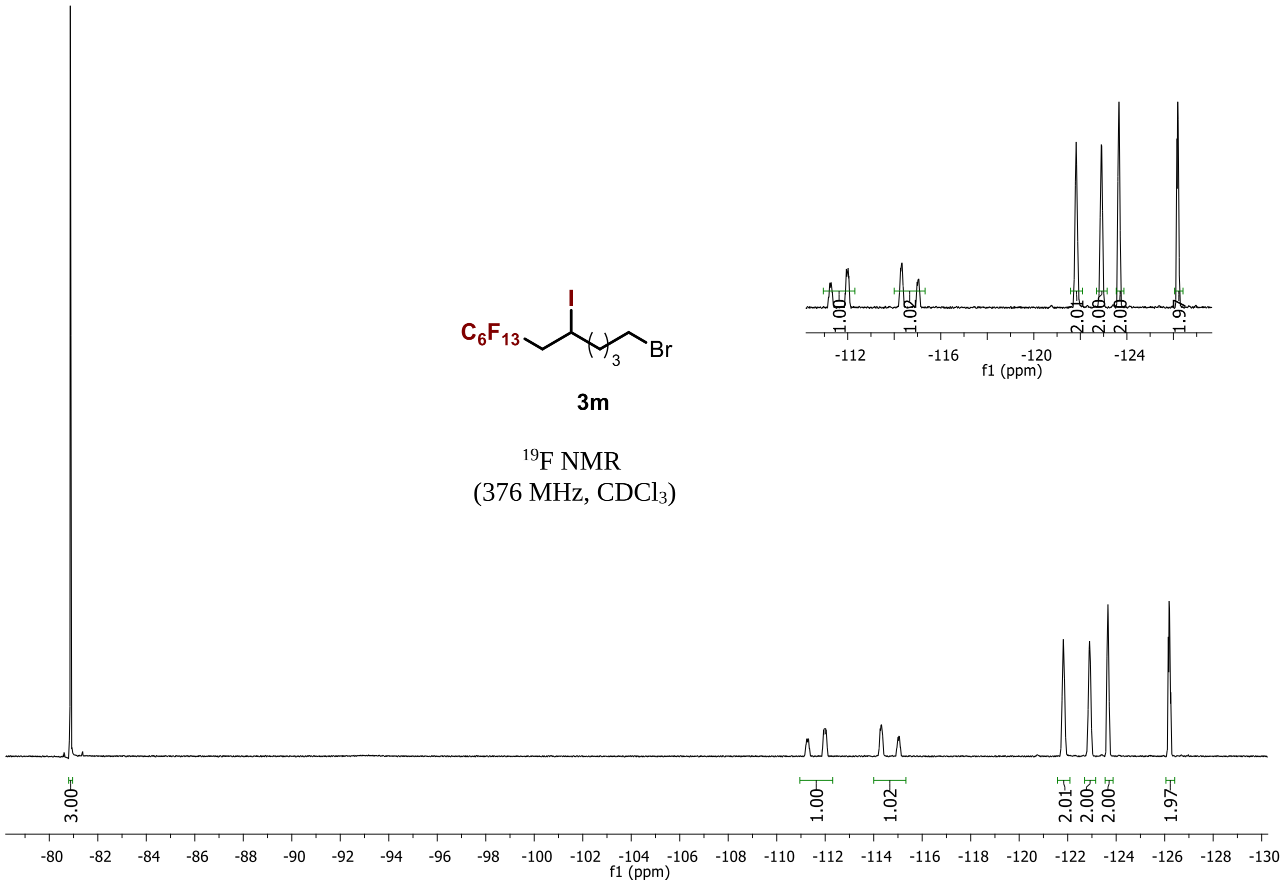

3m

^{19}F NMR
(376 MHz, $CDCl_3$)

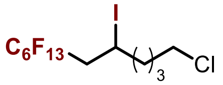

3n

^{1}H NMR (400 MHz, $CDCl_3$)

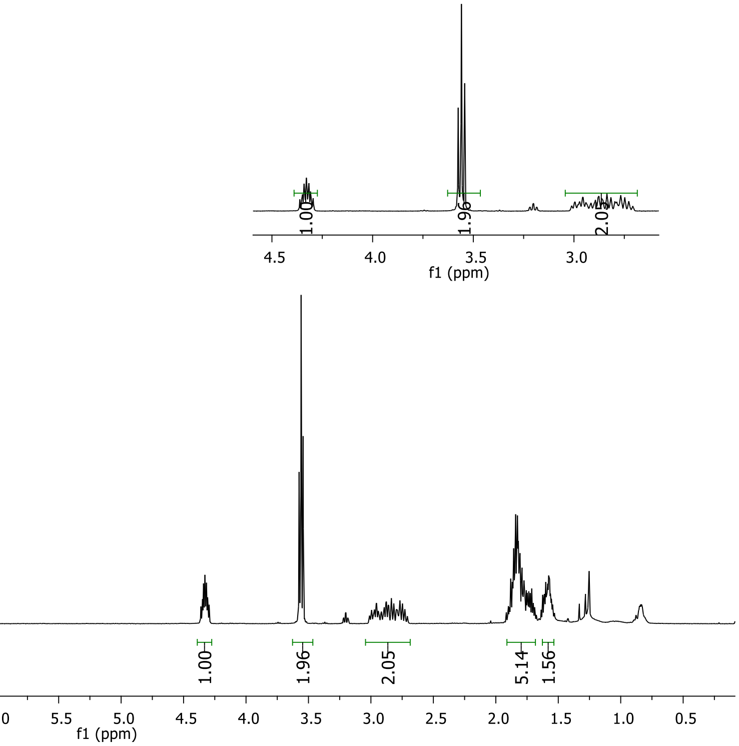

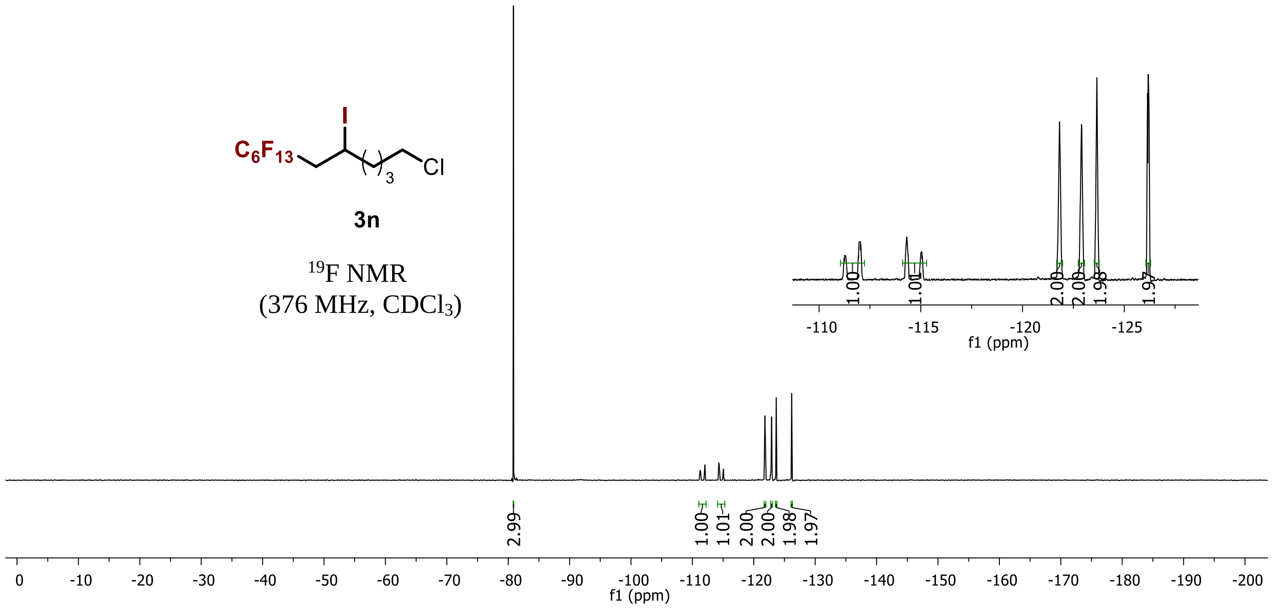

C6F13
I
3
Cl
3n
19F NMR
(376 MHz, CDCl3)
2.99
1.00
1.01
2.00
2.00
1.98
1.97
0
-10
-20
-30
-40
-50
-60
-70
-80
-90
-100
-110
-120
-130
-140
-150
-160
-170
-180
-190
-200
f1 (ppm)
-110
-115
-120
-125
f1 (ppm)

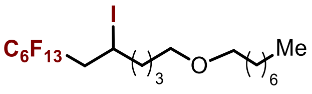

3o

^{1}H NMR (400 MHz, $CDCl_3$)

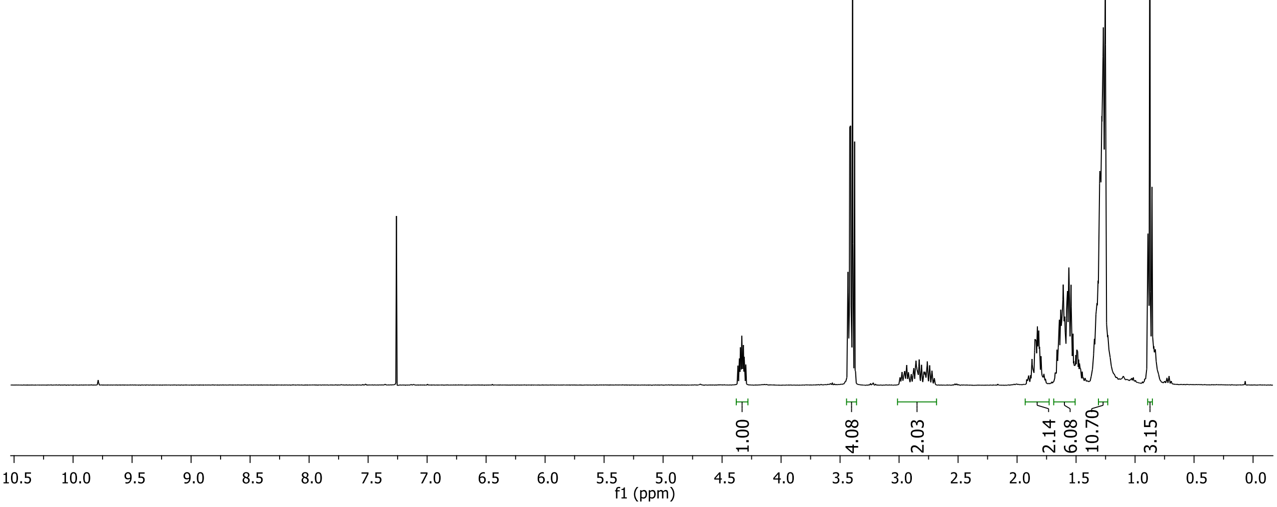

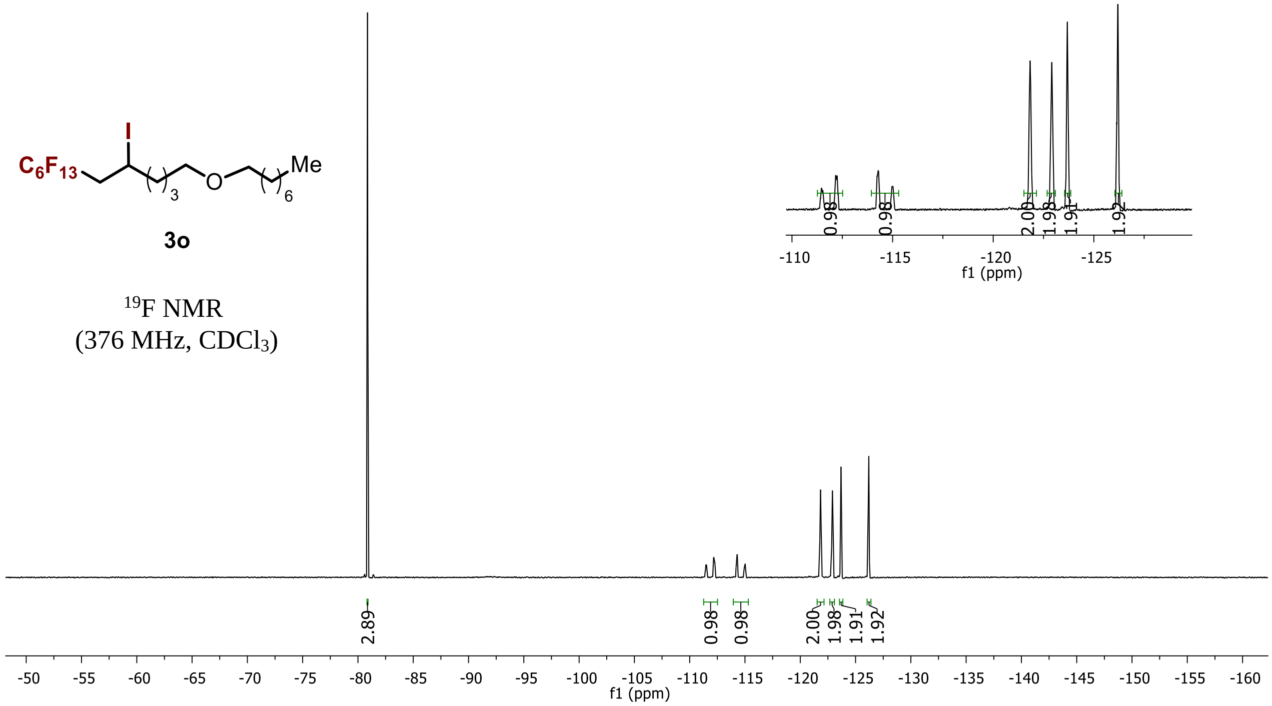

3o

^{19}F NMR
(376 MHz, $CDCl_3$)

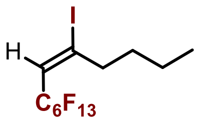

3p

^{1}H NMR (400 MHz, $CDCl_3$)

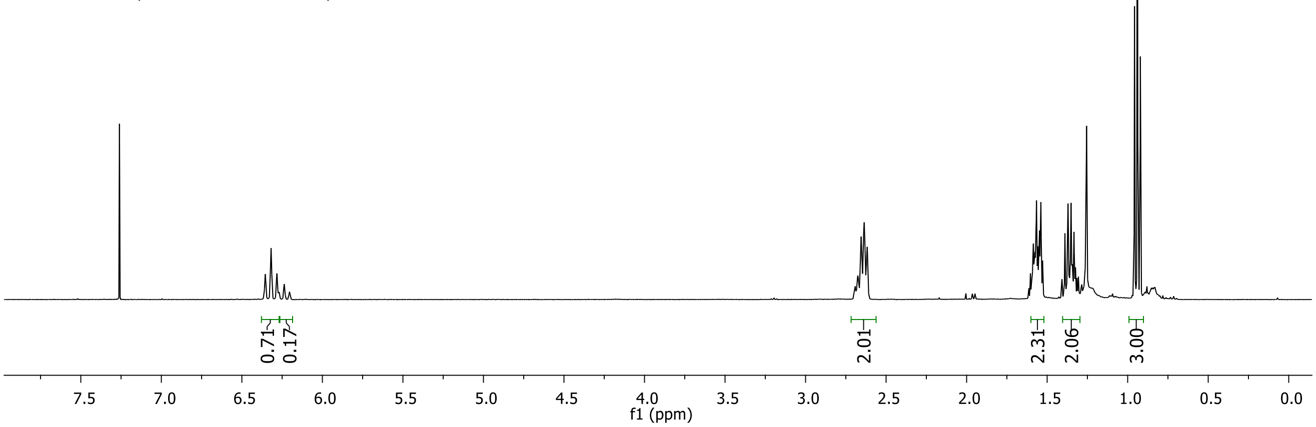

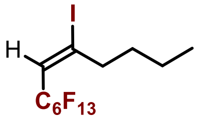

3p

^{19}F NMR
(376 MHz, $CDCl_3$)

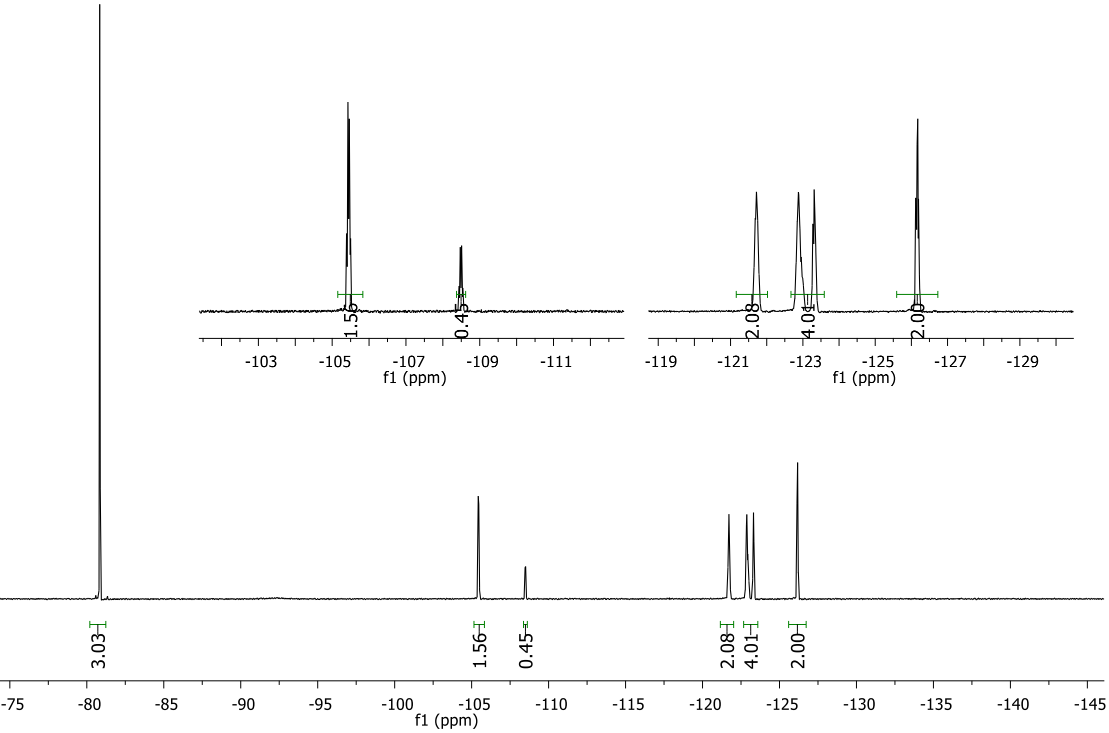

^{19}F NMR surface relaxivities

Further insights into the relaxation behaviour can be drawn by calculation of surface relaxivities. In order to do this, we consider the biphasic fast-exchange model. According to this model, fluids in contact with solid surfaces exhibit NMR relaxation times that can be very different from those of the same liquid as bulk, which can be described according to:

$$\frac{1}{T_{1,2}} = \frac{1}{T_{1,2,bulk}} + \rho_{1,2}\frac{S}{V} \qquad \text{Equation (S1)}$$

The first term is the contribution of the bulk, the second term is the contribution of the surface, whereby $\rho_{1,2}$ are the relaxivities associated to $T_{1,2}$, and $\frac{S}{V}$ is the surface-to-volume ratio. Surface relaxivities characterise the ability of the surface to facilitate relaxation and are related to the adsorbate/adsorbent affinity. Assuming cylindrical pores, the expression can be written in terms of pore diameter as:

$$\frac{1}{T_{1,2}} = \frac{1}{T_{1,2,bulk}} + \rho_{1,2}\frac{4}{d} \qquad \text{Equation (S2)}$$

Usually the bulk term is much larger than the surface term, hence the approximation:

$$\frac{1}{T_{1,2}} \approx \rho_{1,2}\frac{4}{d} \qquad \text{Equation (S3)}$$

Hence, by knowing the typical value of pore diameter d of the solid material it is possible to calculate ρ_1 and ρ_2. The ratio $\rho_1/\rho_2 \approx T_1/T_2$.

Values of ρ_1 and $\rho_2$are reported in Table S2, together with single values of T_1 and T_2. It can be observed that *am*-CN has by far the highest ρ_1 and ρ_2 values among all materials. Differences in ρ_2 across all samples are much more evident compared to ρ_1. In particular, it can be seen that the most active samples, *am*-CN and *red*-CN, have values of ρ_2 which exceeds by far the least active samples, *ox*-CN and *g*-CN. At this point it is worth noting that whilst T_1 is more representative of the overall pore structure, T_2 is more affected by the local surface chemistry (refer to Gladden and Mitchell, *Measuring adsorption, diffusion and flow in chemical engineering: applications of magnetic resonance to porous media*). The results strongly suggest then that the different preparation conditions of the solids affect significantly the local surface chemistry of the final CN photocatalyst.

^{1}H NMR relaxation studies of DMF

Additional information into other key surface interactions within the materials studied here were gathered by ^{1}H NMR T_1/T_2 relaxation measurements using the reaction solvent, dimethylformamide (DMF), as probe molecules. Figure S5 contains a typical data set, which shows the ^{1}H NMR spectrum of DMF adsorbed within the *ox*-CN catalyst and the corresponding T_2 CPMG signal decay. Values of T_1, T_2 and T_1/T_2 values for DMF imbibed within the different photocatalysts are summarised in Table S2. A graphical representation of the measured T_1/T_2 values can be seen in Figure S6.

By looking at the data obtained, it is possible to identify a clear trend in T_1/T_2 ratio value for DMF imbibed within the pores of the various photocatalysts, which reflects a trend in strength of solvent/catalyst surface interactions:

T_1/T_2 [*ox*-CN] > T_1/T_2 [*g*-CN] > T_1/T_2 [*red*-CN] > T_1/T_2 [*am*-CN]

It is interesting to note that the observed trend is inverse to that of activity. This implies that catalysts showing a high affinity for the solvent, that is, a high T_1/T_2 ratio, are those showing the lowest catalytic activity. This result suggests that solvent inhibition play an important in affecting catalyst activity, similarly to what has previously been showing in other heterogeneous catalytic reactions(*51*) meaning that DMF should have as little interaction as possible with the catalyst surface, so that it can be easily displaced by the reagent and therefore preventing the blocking of catalaytically active sites.

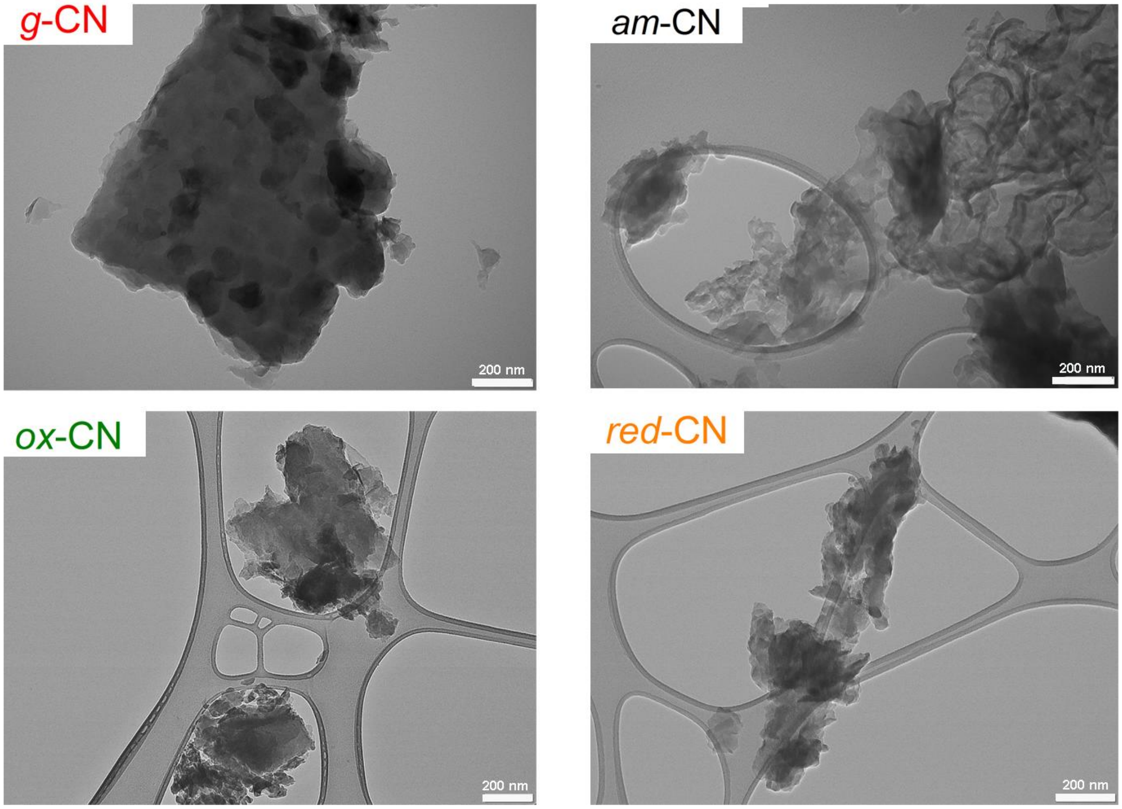

Fig. S1.
Representative TEM images of the CN samples.

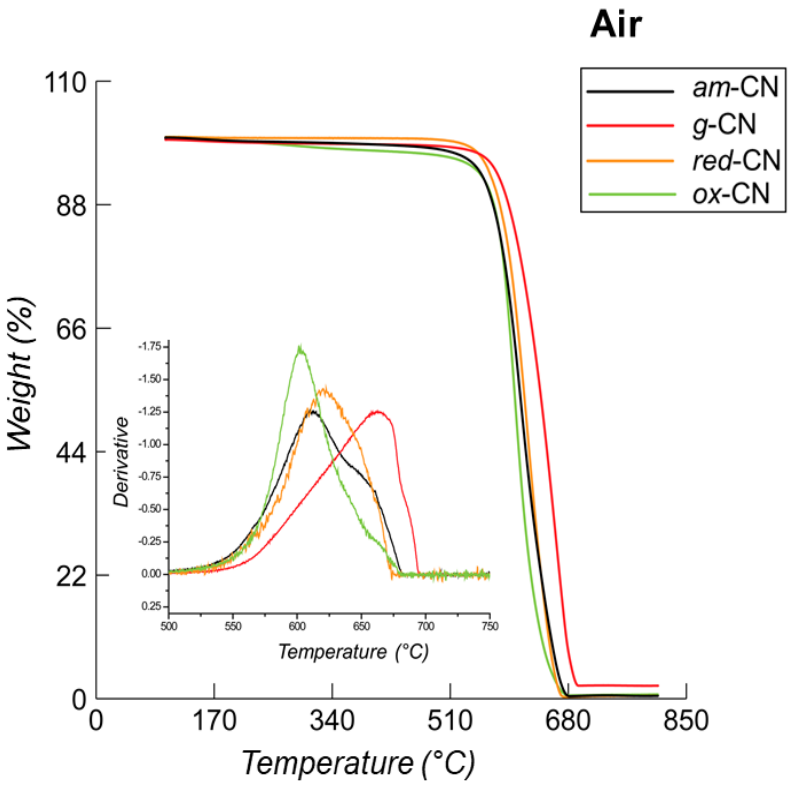

Fig. S2.
TGA analysis of the four materials under air. The combustion temperature onset of all the modified CN is reduced by ~50 °C as compared to the *g*-CN, as better observed in the figure inset, where the weight derivative is shown.

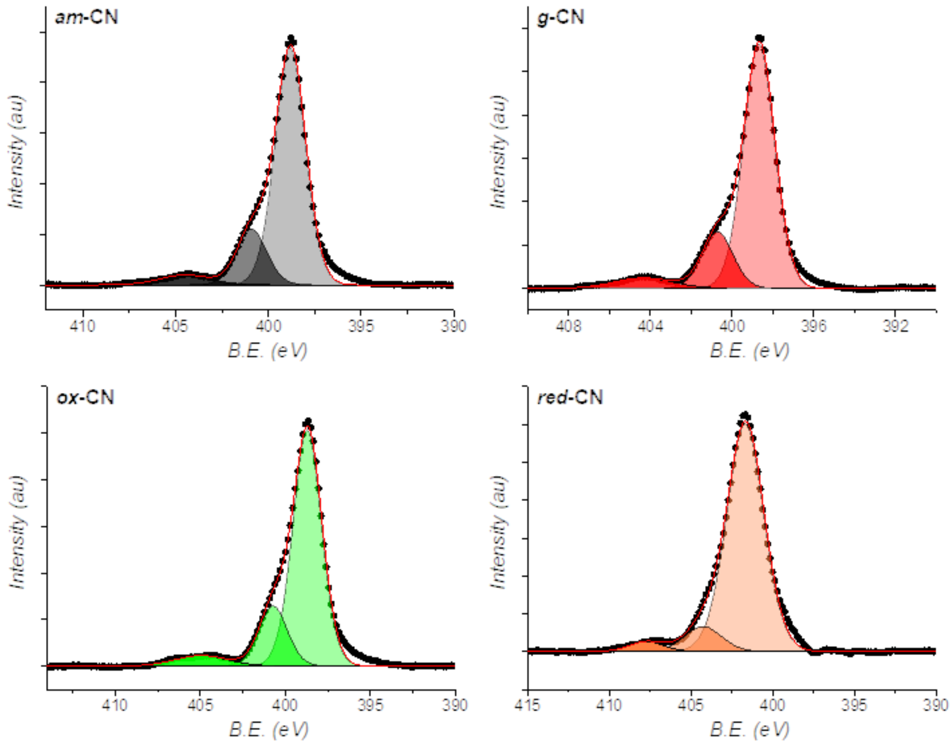

Fig. S3.
XPS showing the deconvoluted N1s core level spectra.

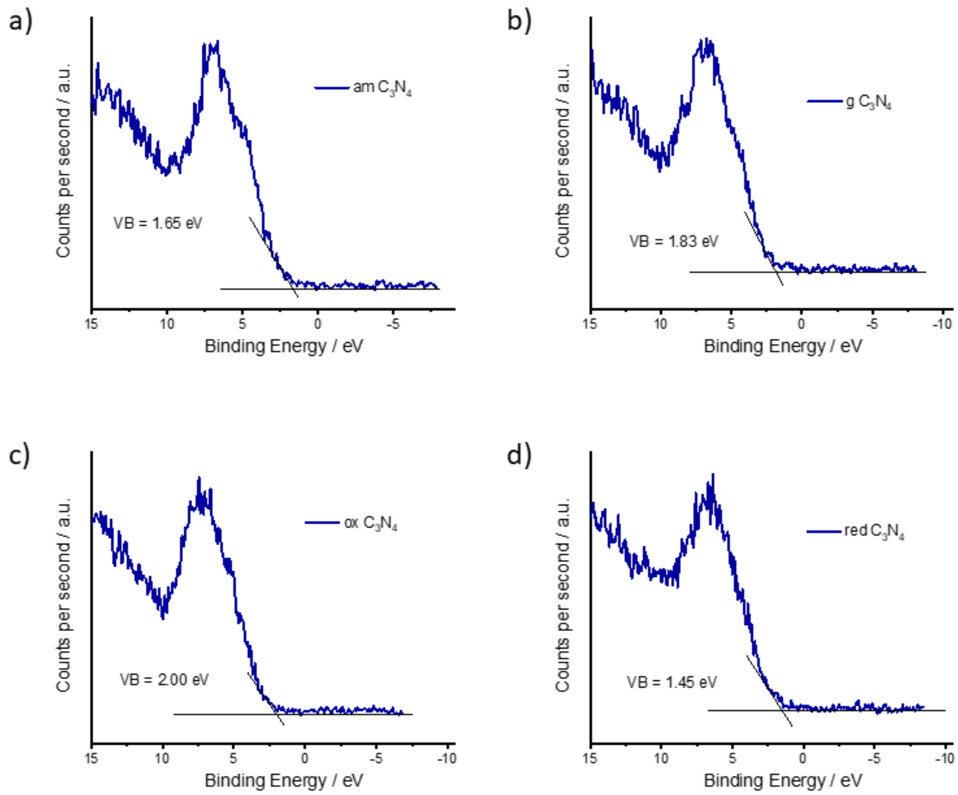

Fig. S4.
XPS of the four samples in the low binding energy range. The VB is calculated from the intercept of the onset of the first peak with the baseline.

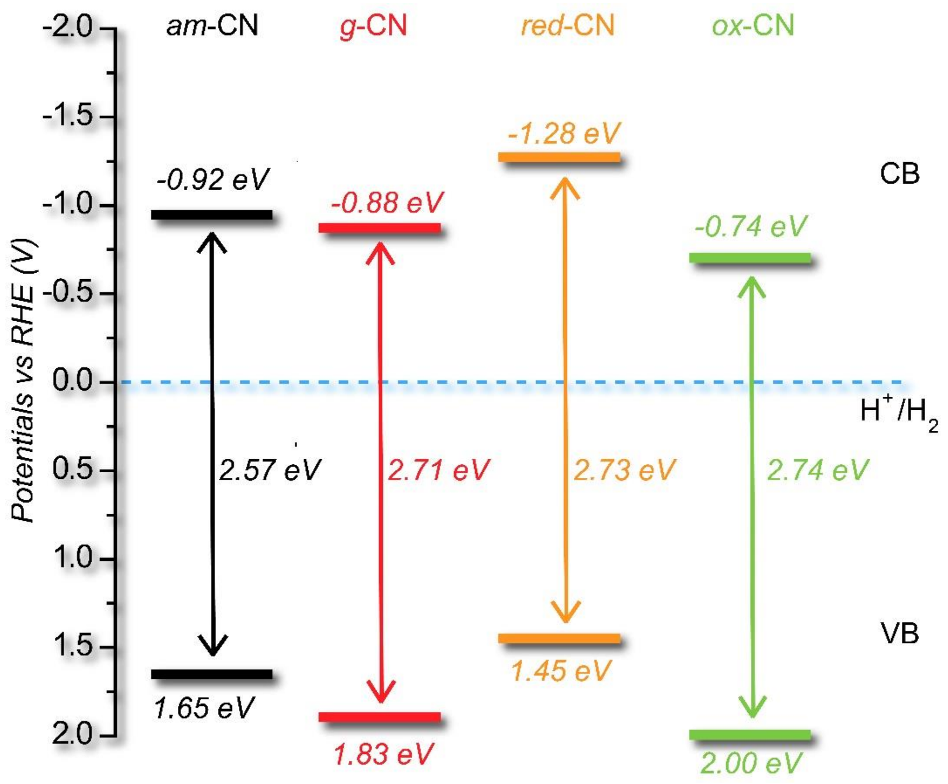

Fig. S5.
Schematic representation of the VB and CB positions for the four materials with energy values in eV. RHE stands for Reversible Hydrogen Electrode.

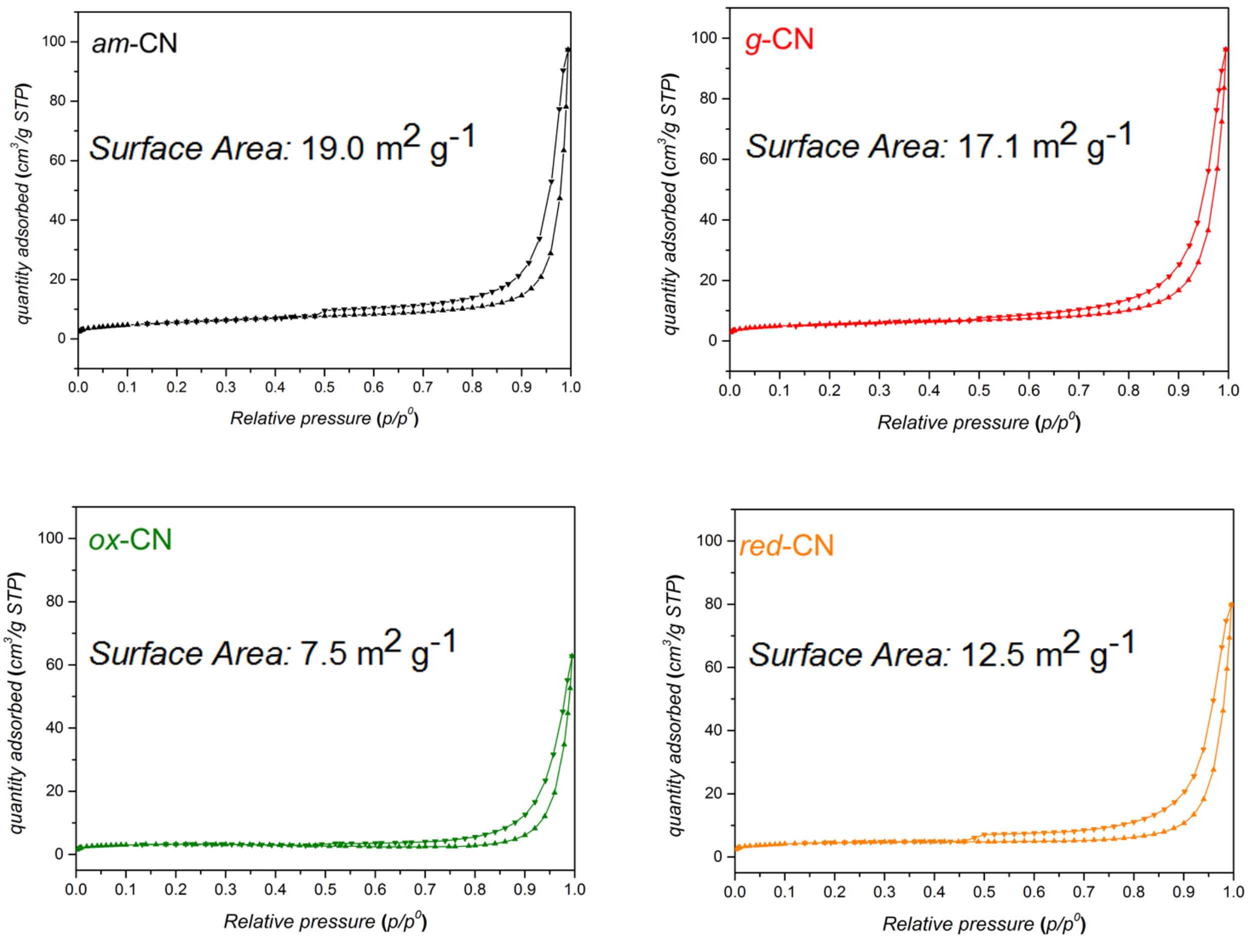

Fig. S6.
N_2 physisorption isotherms of the four materials, with report of their specific surface area.

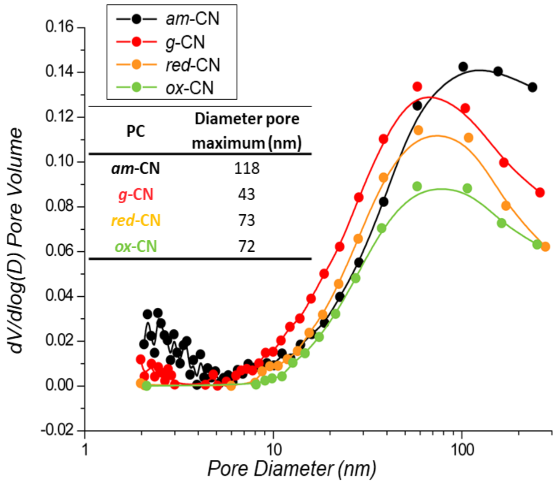

Fig. S7.
Pore size distribution (inset reporting the maximum of the curve).

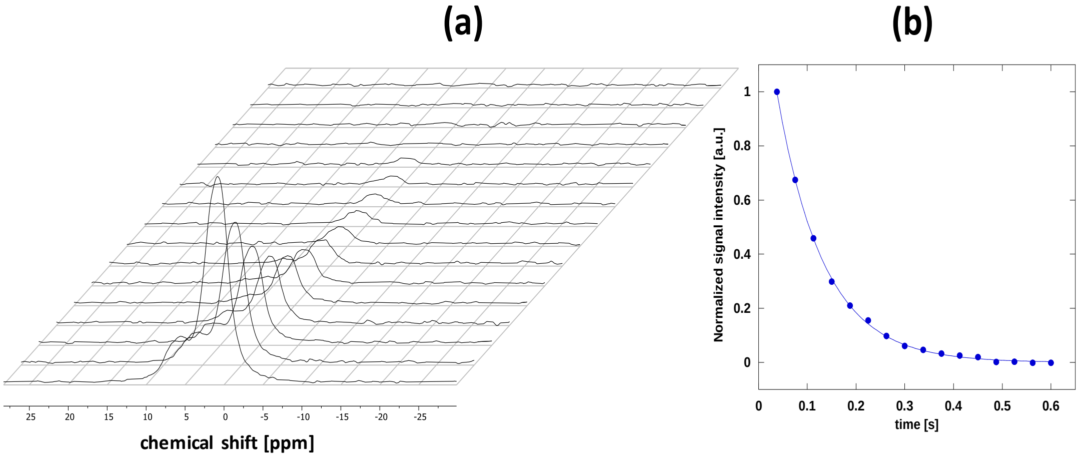

Fig. S8.
^{1}H NMR T_1/T_2 relaxation measurements with DMF. (a) T_2 CPMG spectra of DMF in *ox*-CN and (b) the corresponding T_2 CPMG decay plot for the whole spectrum.

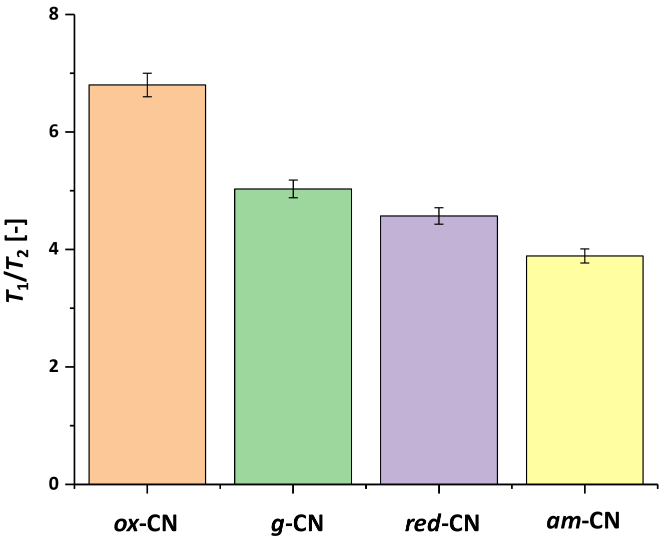

Fig. S9.
T_1/T_2 ratio of DMF imbibed within the various CN-based photocatalytic materials used in this work.

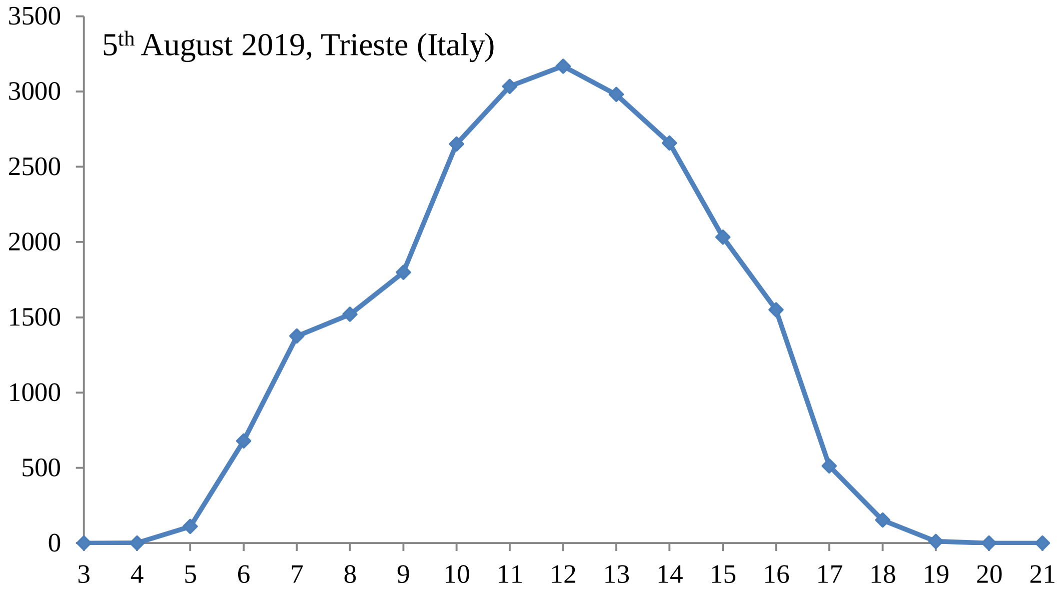

Fig S10.
Outdoor experiment: solar irradiation over time the 5th of August 2019, in Trieste

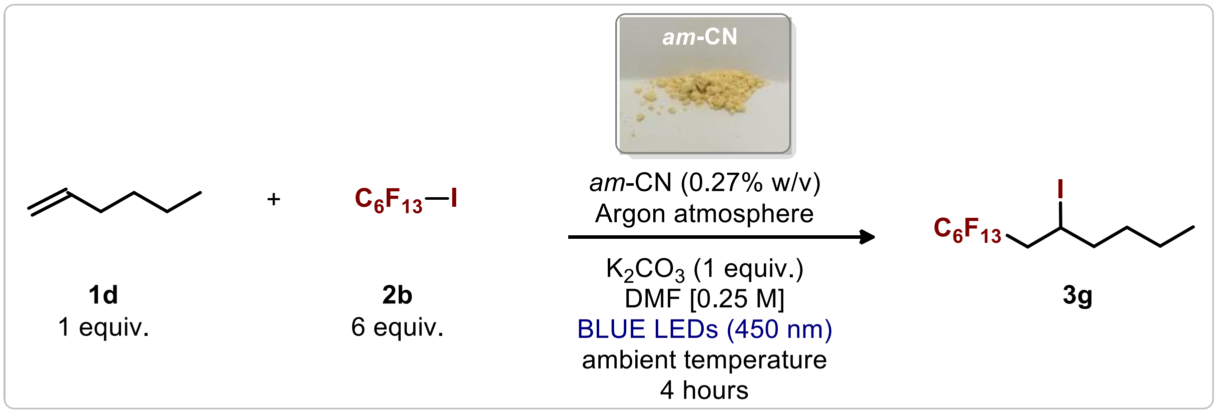

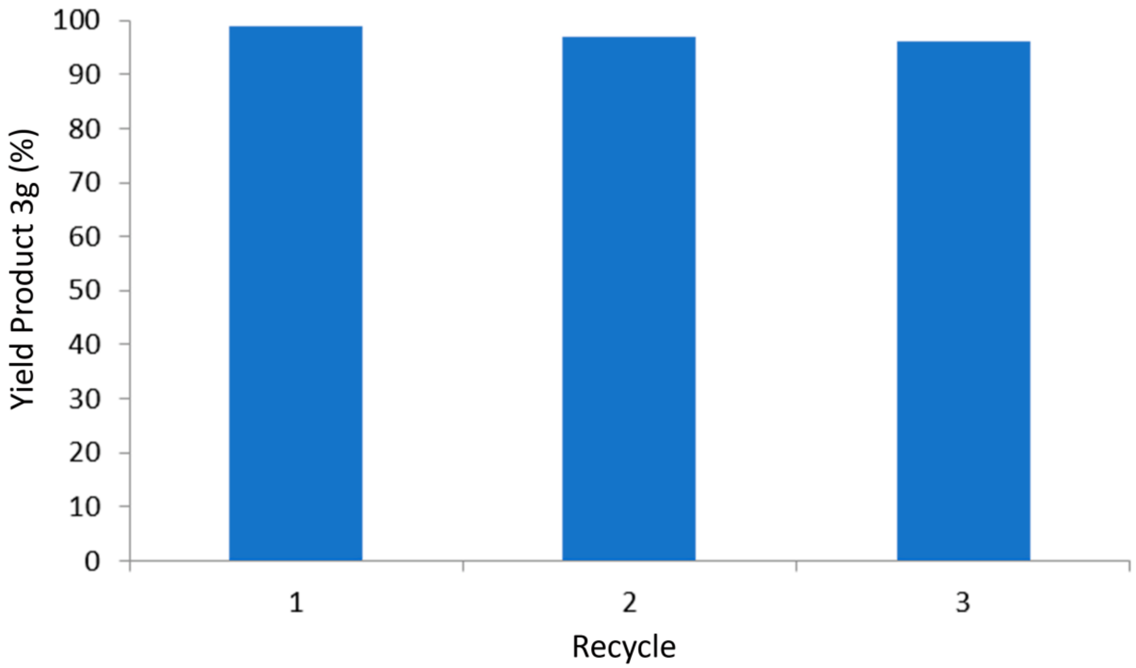

Fig S11.
Recycling tests for reaction producing **3g** with *am*-CN. Photo credit: Francesco Longobardo, University of Trieste, Italy.

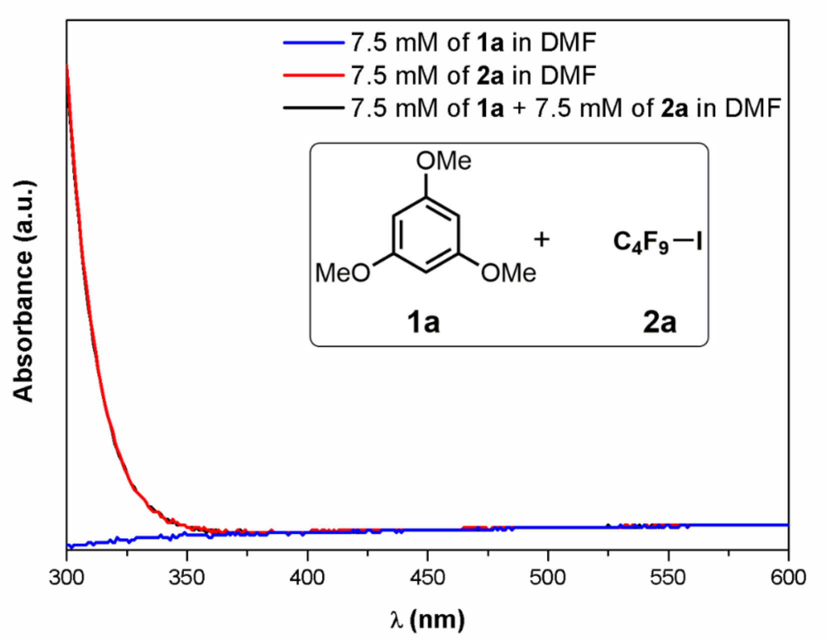

Fig. S12.
Uv-vis spectra of trimethoxybenzene (**1a**), perfluorobutyl iodide **(2a**) and their combination (optical length 1 cm).

Table S1. XPS analysis.

<table>
<tr><td>Sample</td><td colspan="2">C / at%</td><td>N / at%</td><td>O / at%</td></tr>
<tr><td>am-CN</td><td colspan="2">43.13</td><td>56.87</td><td>-</td></tr>
<tr><td>g-CN</td><td colspan="2">42.33</td><td>57.67</td><td>-</td></tr>
<tr><td>ox-CN</td><td colspan="2">41.94</td><td>52.71</td><td>5.35</td></tr>
<tr><td>red-CN</td><td colspan="2">45.00</td><td>55.00</td><td>-</td></tr>
<tr><td colspan="5">Components derived from fitting analysis.</td></tr>
<tr><td>Sample</td><td>Core level</td><td>Component</td><td>Binding Energy / eV</td><td>Area / %</td></tr>
<tr><td rowspan="7">am-CN</td><td rowspan="4">C1S</td><td>C-C</td><td>284.80</td><td>7.20</td></tr>
<tr><td>C-N</td><td>286.22</td><td>8.29</td></tr>
<tr><td>C=N-C</td><td>288.23</td><td>80.80</td></tr>
<tr><td>pi-pi*</td><td>293.69</td><td>3.70</td></tr>
<tr><td rowspan="3">N1S</td><td>C=N-C</td><td>398.80</td><td>76.50</td></tr>
<tr><td>N-(C)3</td><td>400.87</td><td>18.05</td></tr>
<tr><td>N-N[b]</td><td>404.49</td><td>5.45</td></tr>
<tr><td rowspan="7">g-CN</td><td rowspan="4">C1S</td><td>C-C</td><td>284.80</td><td>4.16</td></tr>
<tr><td>C-N</td><td>286.11</td><td>6.20</td></tr>
<tr><td>C=N-C</td><td>288.11</td><td>86.11</td></tr>
<tr><td>pi-pi*</td><td>293.59</td><td>3.53</td></tr>
<tr><td rowspan="3">N1S</td><td>C=N-C</td><td>398.65</td><td>75.98</td></tr>
<tr><td>N-(C)3</td><td>400.69</td><td>17.60</td></tr>
<tr><td>N-N[b]</td><td>404.32</td><td>6.43</td></tr>
<tr><td rowspan="7">ox-CN</td><td rowspan="4">C1S</td><td>C-C</td><td>284.80</td><td>6.78</td></tr>
<tr><td>C-N</td><td>286.04</td><td>7.26</td></tr>
<tr><td>C=N-C</td><td>288.12</td><td>82.70</td></tr>
<tr><td>pi-pi*</td><td>293.50</td><td>3.27</td></tr>
<tr><td rowspan="3">N1S</td><td>C=N-C</td><td>398.67</td><td>74.6</td></tr>
<tr><td>N-(C)3</td><td>400.68</td><td>19.06</td></tr>
<tr><td>N-N[b]</td><td>404.93</td><td>6.34</td></tr>
<tr><td rowspan="7">red-CN</td><td rowspan="4">C1S</td><td>C-C</td><td>284.80</td><td>5.28</td></tr>
<tr><td>C-N</td><td>286.36</td><td>13.52</td></tr>
<tr><td>C=N-C</td><td>288.14</td><td>76.89</td></tr>
<tr><td>pi-pi*</td><td>293.79</td><td>4.31</td></tr>
<tr><td rowspan="3">N1S</td><td>C=N-C</td><td>398.59</td><td>86.76</td></tr>
<tr><td>N-(C)3</td><td>401.18</td><td>9.59</td></tr>
<tr><td>N-N</td><td>404.66</td><td>3.65</td></tr>
</table>

Table S2. Elemental analysis of the four CN materials.

Sample	C (%)	N (%)	H (%)
am-CN	34.52	59.85	1.640
g-CN	34.67	61.36	1.720
red-CN	34.42	60.66	1.740
ox-CN	33.96	59.87	1.780

Table S3. Rates of production (per gram of catalyst and per square meter of catalyst) for reaction generating product **3a**.

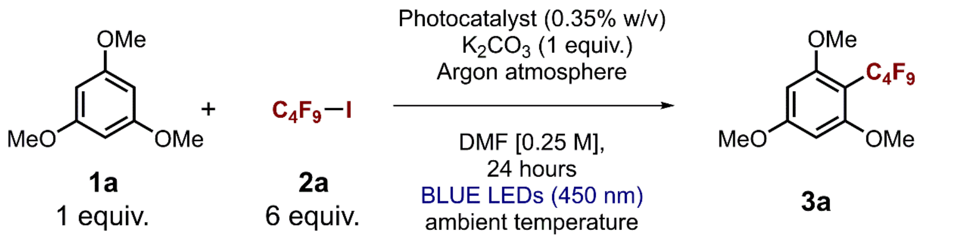

Entry	Photocatalyst	*Yield* **3a**	*Rate of Production* ($mmol_{3a}$ g^{-1} h^{-1})	($mmol_{3a}$ m^{-2} h^{-1})
1	*g*-CN	20%	0.46	0.027
2	*ox*-CN	10%	0.23	0.030
3	*red*-CN	90%	2.08	0.166
4	*am*-CN	>99%	2.32	0.122

Table S4. Values of ^{19}F NMR T_1, T_2, T_1/T_2 and surface relaxivities, ρ_1 and ρ_2, of different moieties of perfluorohexyl iodide within different photocatalysts.

Sample	NMR peak	T_1 [ms]	T_2 [ms]	T_1/T_2 [-]	ρ_1 [nm/s]	ρ_2 [nm/s]
g-CN	CF_2I	1124 ± 33	323 ± 9	3.48 ± 0.17	9.35 ± 0.28	32.55 ± 0.98
	$(CF_2)_4$	1190 ± 35	417 ± 12	2.86 ± 0.14	8.82 ± 0.26	25.20 ± 0.75
	CF_3	1163 ± 34	455 ± 12	2.56 ± 0.13	9.01 ± 0.27	23.10 ± 0.69
ox-CN	CF_2I	1136 ± 33	286 ± 8	3.98 ± 0.18	15.84 ± 0.47	63.00 ± 1.89
	$(CF_2)_4$	1250 ± 37	385 ± 11	3.25 ± 0.16	14.40 ± 0.43	46.80 ± 1.40
	CF_3	1235± 36	435 ± 13	2.84 ± 0.14	14.58 ± 0.44	41.40 ± 1.24
red-CN	CF_2I	1149 ± 34	147 ± 4	7.82 ± 0.39	16.10 ± 0.48	124.10 ± 3.72
	$(CF_2)_4$	1149 ± 34	196 ± 5	5.86 ± 0.25	15.88 ± 0.47	93.08 ± 2.79
	CF_3	1176 ± 35	204 ± 5	5.76 ± 0.29	15.51 ± 0.46	89.43 ± 2.68
am-CN	CF_2I	1042 ± 30	128 ± 3	8.13 ± 0.40	28.32 ± 0.85	230.10 ± 6.90
	$(CF_2)_4$	1010 ± 29	159 ± 4	6.36 ± 0.32	29.21 ± 0.87	185.85 ± 5.55
	CF_3	1053 ± 31	164 ± 4	6.42 ± 0.33	28.03 ± 0.84	179.95 ± 5.93

Table S5. Values of ^{1}H NMR T_1, T_2 and T_1/T_2 of the DMF solvent within different photocatalysts.

Sample	T_1 [ms]	T_2 [ms]	T_1/T_2 [-]
g-C_3N_4	2133 ± 64	424 ± 13	5.03 ± 0.15
ox-C_3N_4	653 ± 20	96 ± 3	6.80 ± 0.20
red-C_3N_4	1722 ± 52	377 ± 11	4.57 ± 0.14
am-C_3N_4	1929 ± 58	496 ± 15	3.89 ± 0.12

* Relative errors are in the range 3-5%; for bulk DMF $T_1 = T_2 = 3501$ ms.

Table S6. Comparison of the photocatalytic activity with different LED wavelengths for the production of **3a** with the four catalysts.

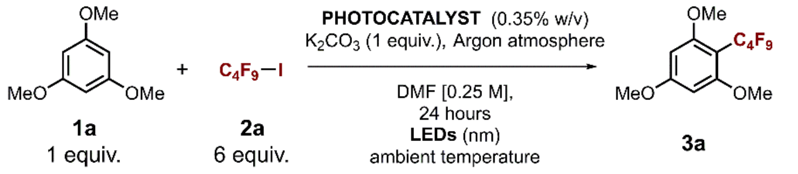

Entry	*Photocatalyst*	LEDs (nm)	*Yield*
1	*g*-CN	**450**	20%
2	*g*-CN	**395**	17%
3	*ox*-CN	**450**	10%
4	*ox*-CN	**395**	13%
5	*red*-CN	**450**	90%
6	*red*-CN	**395**	87%
7	*am*-CN	**450**	>99%
8	*am*-CN	**395**	98%

Table S7. Comparison between the activity of photocatalyst *am*-CN with other reported photocatalysts for production of compound **3k**. Photo credit: Francesco Longobardo, University of Trieste, Italy.

$$\textit{Rate of Production}\ [\mathrm{mmol\ h^{-1}\ g^{-1}}] = \frac{\text{Yield of Product } \mathbf{3k}\ [\mathrm{mmol}]}{\text{Reaction Time [hours] x Weight of Photocatalyst [g]}}$$

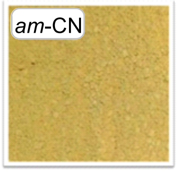

16.79 mmol h^{-1} g^{-1}

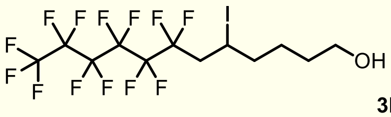

3k

Selected Photocatalysts

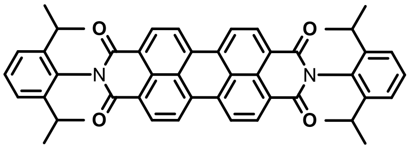

693 mmol h^{-1} g^{-1}

ChemPhotoChem **2019**, *3*, 193-197

Ru[(bpy)$_3$]Cl$_2$

260 mmol h^{-1} g^{-1}

J. Am. Chem. Soc. **2012,** *134***,** 8875-8884

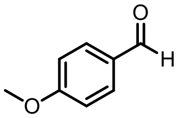

1.45 mmol h^{-1} g^{-1}

Angew. Chem. Int. Ed. **2014**, *53*, 12064-12068

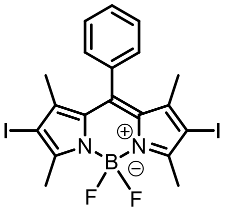

2.92 mmol h^{-1} g^{-1}

Chem. Commun. **2017**, *53,* 1591-1594

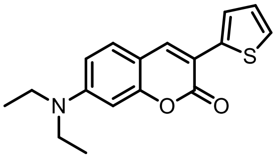

1.70 mmol h^{-1} g^{-1}

Chem. Commun. **2018**, *54*, 10044-10047

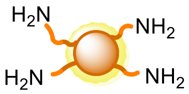

amine-rich N-doped carbon nanodots (NCNDs)

0.97 mmol h^{-1} g^{-1}

Chem. Eur. J. **2019**, *25*, 16032-16036

Table S8. Limitation of the reported photochemical transformation using catalyst *am*-CN.

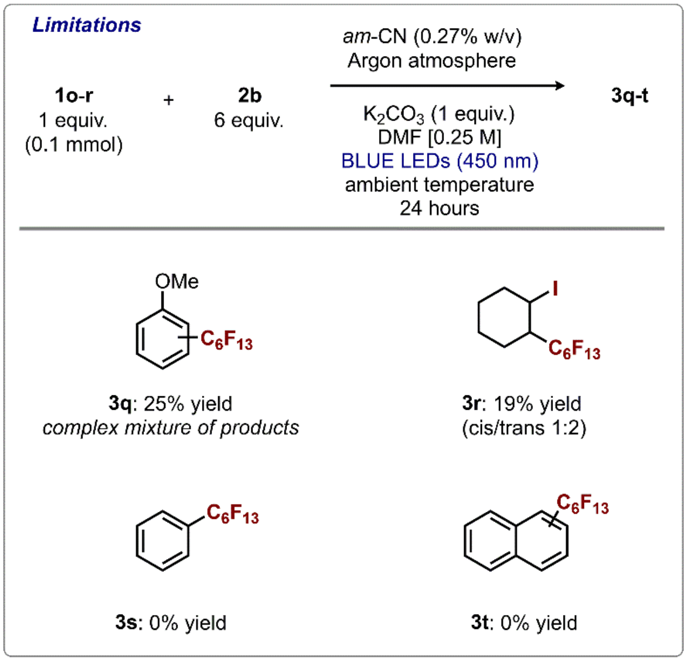

Table S9. Photocatalytic tests with different radical precursors with higher redox potential.

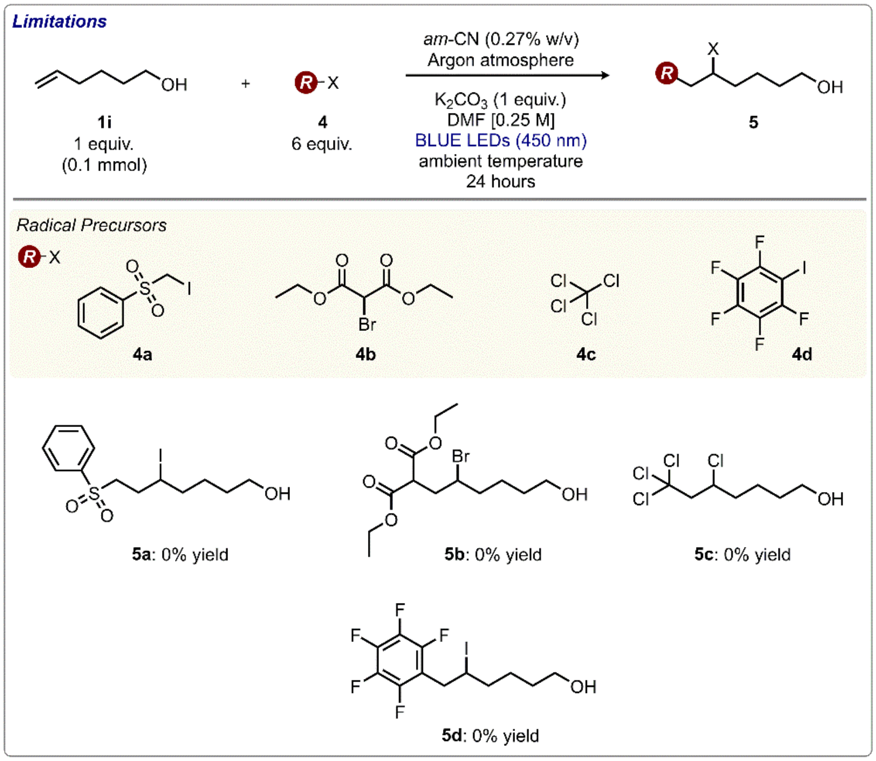

REFERENCES AND NOTES

1. S. Cao, J. Low, J. Yu, M. Jaroniec, Polymeric photocatalysts based on graphitic carbon nitride. *Adv. Mater.* **27**, 2150–2176 (2015).

2. J. Liu, H. Wang, M. Antonietti, Graphitic carbon nitride "reloaded": Emerging applications beyond (photo)catalysis. *Chem. Soc. Rev.* **45**, 2308–2326 (2016).

3. J. Zhang, Y. Chen, X. Wang, Two-dimensional covalent carbon nitride nanosheets: Synthesis, functionalization, and applications. *Energ. Environ. Sci.* **8**, 3092–3108 (2015).

4. Y. Wang, X. Wang, M. Antonietti, Polymeric graphitic carbon nitride as a heterogeneous organocatalyst: From photochemistry to multipurpose catalysis to sustainable chemistry. *Angew. Chem. Int. Ed.* **51**, 68–89 (2012).

5. Y. Zhang, A. Thomas, M. Antonietti, X. Wang, Activation of carbon nitride solids by protonation: Morphology changes, enhanced ionic conductivity, and photoconduction experiments. *J. Am. Chem. Soc.* **131**, 50–51 (2009).

6. G. Liu, P. Niu, C. Sun, S. C. Smith, Z. Chen, G. Q. Lu, H.-M. Cheng, Unique electronic structure induced high photoreactivity of sulfur-doped graphitic C_3N_4. *J. Am. Chem. Soc.* **132**, 11642–11648 (2010).

7. Y. Wang, Y. Di, M. Antonietti, H. Li, X. Chen, X. Wang, Excellent visible-light photocatalysis of fluorinated polymeric carbon nitride solids. *Chem. Mater.* **22**, 5119–5121 (2010).

8. H. Pan, Y.-W. Zhang, V. B. Shenoy, H. Gao, Ab initio study on a novel photocatalyst: Functionalized graphitic carbon nitride nanotube. *ACS Catal.* **1**, 99–104 (2011).

9. X. Wang, X. Chen, A. Thomas, X. Fu, M. Antonietti, Metal-containing carbon nitride compounds: A new functional organic–metal hybrid material. *Adv. Mater.* **21**, 1609–1612 (2009).

10. X. Ye, Y. Cui, X. Qiu, X. Wang, Selective oxidation of benzene to phenol by Fe-CN/TS-1 catalysts under visible light irradiation. *Appl. Catal. B Environ.* **152-153**, 383–389 (2014).

11. X. Chen, J. Zhang, X. Fu, M. Antonietti, X. Wang, Fe-g-C_3N_4-catalyzed oxidation of benzene to phenol using hydrogen peroxide and visible light. *J. Am. Chem. Soc.* **131**, 11658–11659 (2009).

12. L. Möhlmann, M. Baar, J. Rieß, M. Antonietti, X. Wang, S. Blechert, Carbon nitride-catalyzed photoredox C—C bond formation with *N*-aryltetrahydroisoquinolines. *Adv. Synth. Catal.* **354**, 1909–1913 (2012).

13. C. Cavedon, A. Madani, P. H. Seeberger, B. Pieber, Semiheterogeneous dual nickel/photocatalytic (thio)etherification using carbon nitrides. *Org. Lett.* **21**, 5331–5334 (2019).

14. B. Pieber, J. A. Malik, C. Cavedon, S. Gisbertz, A. Savateev, D. Cruz, T. Heil, G. Zhang, P. H. Seeberger, Semi-heterogeneous dual nickel/photocatalysis using carbon nitrides: Esterification of carboxylic acids with aryl halides. *Angew. Chem. Int. Ed.* **58**, 9575–9580 (2019).

15. B. Pieber, M. Shalom, M. Antonietti, P. H. Seeberger, K. Gilmore, Continuous heterogeneous photocatalysis in serial micro-batch reactors. *Angew. Chem. Int. Ed.* **57**, 9976–9979 (2018).

16. I. Ghosh, J. Khamrai, A. Savateev, N. Shlapakov, M. Antonietti, B. König, Organic semiconductor photocatalyst can bifunctionalize arenes and heteroarenes. *Science* **365**, 360–366 (2019).

17. K. Müller, C. Faeh, F. Diederich, Fluorine in pharmaceuticals: Looking beyond intuition. *Science* **317**, 1881–1886 (2007).

18. Y. Zhou, J. Wang, Z. Gu, S. Wang, W. Zhu, J. L. Aceña, V. A. Soloshonok, K. Izawa, H. Liu, Next generation of fluorine-containing pharmaceuticals, compounds currently in phase II–III clinical trials of major pharmaceutical companies: New structural trends and therapeutic areas. *Chem. Rev.* **116**, 422–518 (2016).

19. R. Berger, G. Resnati, P. Metrangolo, E. Weber, J. Hulliger, Organic fluorine compounds: A great opportunity for enhanced materials properties. *Chem. Soc. Rev.* **40**, 3496–3508 (2011).

20. C.-J. Wallentin, J. D. Nguyen, P. Finkbeiner, C. R. J. Stephenson, Visible light-mediated atom transfer radical addition via oxidative and reductive quenching of photocatalysts. *J. Am. Chem. Soc.* **134**, 8875–8884 (2012).

21. S. Barata-Vallejo, M. V. Cooke, A. Postigo, Radical fluoroalkylation reactions. *ACS Catal.* **8**, 7287–7307 (2018).

22. D. A. Nagib, D. W. C. MacMillan, Trifluoromethylation of arenes and heteroarenes by means of photoredox catalysis. *Nature* **480**, 224–228 (2011).

23. G. Magagnano, A. Gualandi, M. Marchini, L. Mengozzi, P. Ceroni, P. G. Cozzi, Photocatalytic ATRA reaction promoted by iodo-Bodipy and sodium ascorbate. *Chem. Commun.* **53**, 1591–1594 (2017).

24. E. Arceo, E. Montroni, P. Melchiorre, Photo-organocatalysis of atom-transfer radical additions to alkenes. *Angew. Chem. Int. Ed.* **53**, 12064–12068 (2014).

25. T. Yajima, M. Ikegami, Metal-free visible-light radical iodoperfluoroalkylation of terminal alkenes and alkynes. *Eur. J. Org. Chem.* **2017**, 2126–2129 (2017).

26. W. R. Dolbier, Structure, reactivity, and chemistry of fluoroalkyl radicals. *Chem. Rev.* **96**, 1557–1584 (1996).

27. P. Niu, L.-C. Yin, Y.-Q. Yang, G. Liu, H.-M. Cheng, Increasing the visible light absorption of graphitic carbon nitride (melon) photocatalysts by homogeneous self-modification with nitrogen vacancies. *Adv. Mater.* **26**, 8046–8052 (2014).

28. M. Melchionna, M. Prato, Functionalizing carbon nanotubes: An indispensible step towards applications. *ECS J. Solid State Sci. Technol.* **2**, M3040–M3045 (2013).

29. Z. Lu, G. Chen, S. Siahrostami, Z. Chen, K. Liu, J. Xie, L. Liao, T. Wu, D. Lin, Y. Liu, T. F. Jaramillo, J. K. Nørskov, Y. Cui, High-efficiency oxygen reduction to hydrogen peroxide catalysed by oxidized carbon materials. *Nat. Catal.* **1**, 156–162 (2018).

30. M. Melchionna, P. Fornasiero, M. Prato, The rise of hydrogen peroxide as the main product by metal-free catalysis in oxygen reductions. *Adv. Mater.* **31**, 1802920 (2019).

31. Y. Kang, Y. Yang, L.-C. Yin, X. Kang, G. Liu, H.-M. Cheng, An amorphous carbon nitride photocatalyst with greatly extended visible-light-responsive range for photocatalytic hydrogen generation. *Adv. Mater.* **27**, 4572–4577 (2015).

32. T. Sano, S. Tsutsui, K. Koike, T. Hirakawa, Y. Teramoto, N. Negishi, K. Takeuchi, Activation of graphitic carbon nitride (g-C_3N_4) by alkaline hydrothermal treatment for photocatalytic NO oxidation in gas phase. *J. Mater. Chem. A* **1**, 6489–6496 (2013).

33. F. Fina, S. K. Callear, G. M. Carins, J. T. S. Irvine, Structural investigation of graphitic carbon nitride via XRD and neutron diffraction. *Chem. Mater.* **27**, 2612–2618 (2015).

34. P. F. McMillan, V. Lees, E. Quirico, G. Montagnac, A. Sella, B. Reynard, P. Simon, E. Bailey, M. Deifallah, F. Corà, Graphitic carbon nitride $C_6N_9H_3{\cdot}HCl$: Characterisation by UV and near-IR FT Raman spectroscopy. *J. Solid State Chem.* **182**, 2670–2677 (2009).

35. P. V. Zinin, L.-C. Ming, S. K. Sharma, V. N. Khabashesku, X. Liu, S. Hong, S. Endo, T. Acosta, Ultraviolet and near-infrared Raman spectroscopy of graphitic C_3N_4 phase. *Chem. Phys. Lett.* **472**, 69–73 (2009).

36. B. Jürgens, E. Irran, J. Senker, P. Kroll, H. Müller, W. Schnick, Melem (2,5,8-Triamino-tri-*s*-triazine), an important intermediate during condensation of melamine rings to graphitic carbon nitride: Synthesis, structure determination by X-ray powder diffractometry, solid-state NMR, and theoretical studies. *J. Am. Chem. Soc.* **125**, 10288–10300 (2003).

37. J. Fang, H. Fan, M. Li, C. Long, Nitrogen self-doped graphitic carbon nitride as efficient visible light photocatalyst for hydrogen evolution. *J. Mater. Chem. A* **3**, 13819–13826 (2015).

38. L. Wang, A. Ambrosi, M. Pumera, "Metal-free" catalytic oxygen reduction reaction on heteroatom-doped graphene is caused by trace metal impurities. *Angew. Chem. Int. Ed.* **52**, 13818–13821 (2013).

39. Y. Liao, S. Zhu, Z. Chen, X. Lou, D. Zhang, A facile method of activating graphitic carbon nitride for enhanced photocatalytic activity. *Phys. Chem. Chem. Phys.* **17**, 27826–27832 (2015).

40. H. Yan, Y. Chen, S. Xu, Synthesis of graphitic carbon nitride by directly heating sulfuric acid treated melamine for enhanced photocatalytic H_2 production from water under visible light. *Int. J. Hydrogen Energy* **37**, 125–133 (2012).

41. K. Schwinghammer, B. Tuffy, M. B. Mesch, E. Wirnhier, C. Martineau, F. Taulelle, W. Schnick, J. Senker, B. V. Lotsch, Triazine-based carbon nitrides for visible-light-driven hydrogen evolution. *Angew. Chem. Int. Ed.* **52**, 2435–2439 (2013).

42. A. Savateev, I. Ghosh, B. König, M. Antonietti, Photoredox catalytic organic transformations using heterogeneous carbon nitrides. *Angew. Chem. Int. Ed.* **57**, 15936–15947 (2018).

43. H. Yan, Soft-templating synthesis of mesoporous graphitic carbon nitride with enhanced photocatalytic H_2 evolution under visible light. *Chem. Commun.* **48**, 3430–3432 (2012).

44. M. Melchionna, P. Fornasiero, Updates on the roadmap for photocatalysis. *ACS Catal.* **10**, 5493–5501 (2020).

45. C. Rosso, G. Filippini, M. Prato, Use of nitrogen-doped carbon nanodots for the photocatalytic fluoroalkylation of organic compounds. *Chem. A Eur. J.* **25**, 16032–16036 (2019).

46. L. Buzzetti, G. E. M. Crisenza, P. Melchiorre, Mechanistic studies in photocatalysis. *Angew. Chem. Int. Ed.* **58**, 3730–3747 (2019).

47. C. D'Agostino, J. Mitchell, M. D. Mantle, L. F. Gladden, Interpretation of NMR relaxation as a tool for characterising the adsorption strength of liquids inside porous materials. *Chem. A Eur. J.* **20**, 13009–13015 (2014).

48. N. Robinson, C. Robertson, L. F. Gladden, S. J. Jenkins, C. D'Agostino, Direct correlation between adsorption energetics and nuclear spin relaxation in a liquid-saturated catalyst material. *ChemPhysChem* **19**, 2472–2479 (2018).

49. N. Robinson, L. F. Gladden, C. D'Agostino, Exploring catalyst passivation with NMR relaxation. *Faraday Discuss.* **204**, 439–452 (2017).

50. P. Politzer, P. Lane, M. C. Concha, Y. Ma, J. S. Murray, An overview of halogen bonding. *J. Mol. Model.* **13**, 305–311 (2007).

51. C. D'Agostino, M. R. Feaviour, G. L. Brett, J. Mitchell, A. P. E. York, G. J. Hutchings, M. D. Mantle, L. F. Gladden, Solvent inhibition in the liquid-phase catalytic oxidation of 1,4-butanediol: Understanding the catalyst behaviour from NMR relaxation time measurements. *Catal. Sci. Technol.* **6**, 7896–7901 (2016).

52. W. R. Bowman, J. M. D. Storey, Synthesis using aromatic homolytic substitution—Recent advances. *Chem. Soc. Rev.* **36**, 1803–1822 (2007).

53. E. Fukushima, S. W. Roeder, *Experimental pulse NMR*, Addison-Weslkey, Reading, US, 1981.

54. C. Rosso, G. Filippini, P. G. Cozzi, A. Gualandi, M. Prato, Highly performing iodoperfluoroalkylation of alkenes triggered by the photochemical activity of perylene diimides. *ChemPhotoChem* **3**, 193–197 (2019).

55. Y. Wang, J. Wang, G.-X. Li, G. He, G. Chen, Halogen-bond-promoted photoactivation of perfluoroalkyl iodides: A photochemical protocol for perfluoroalkylation reactions. *Org. Lett.* **19**, 1442–1445 (2017).

56. L. Cui, Y. Matusaki, N. Tada, T. Miura, B. Uno, A. Itoh, Metal-free direct C—H perfluoroalkylation of arenes and heteroarenes using a photoredox organocatalyst. *Adv. Synth. Catal.* **355**, 2203–2207 (2013).

57. C.-Y. He, J.-W. Gu, X. Zhang, Visible-light-mediated direct perfluoroalkylation and trifluoromethylation of free anilines. *Tetrahedron Lett.* **58**, 3939–3941 (2017).

58. G. Filippini, M. Nappi, P. Melchiorre, Photochemical direct perfluoroalkylation of phenols. *Tetrahedron* **71**, 4535–4542 (2015).

59. K. Aikawa, Y. Nakamura, Y. Yokota, W. Toya, K. Mikami, Stable but reactive perfluoroalkylzinc reagents: Application in ligand-free copper-catalyzed perfluoroalkylation of aryl iodides. *Chem. A Eur. J.* **21**, 96–100 (2015).
